# M1-like macrophages regulate T cell infiltration in colorectal cancer through P2X4 receptor

**DOI:** 10.1016/j.isci.2025.113517

**Published:** 2025-09-05

**Authors:** Kun Zhou, Xintian Zhang, Yu Liang, Han Yao, Yichao Hou, Xingming Zhang, Leilei Du, Wenfeng Wang, Jianhua Wang, Xiangjun Meng

**Affiliations:** 1 Shanghai Key Laboratory of Gut Microecology and Associated Major Diseases Research, Digestive Disease Research and Clinical Translation Center, Department of Gastroenterology, Shanghai Ninth People's Hospital, Shanghai Jiao Tong University School of Medicine, Shanghai 200011, China; 2 Cancer Institute, Shanghai Urological Cancer Institute, Fudan University Shanghai Cancer Center, Fudan University, Shanghai 200032, China

**Keywords:** Microenvironment, Immunology, Cell biology, Cancer

## Abstract

Although tumor-associated macrophages (TAMs) play a critical immunomodulatory role in colorectal cancer (CRC), the mechanisms underlying their polarization remain unclear. This study identifies the P2X4 receptor (P2X4R) as a crucial mediator of M1-like polarization. During TAM induction in a controlled *in vitro* system using CRC cell-conditioned medium, we observed P2X4R-mediated calcium influx and subsequent mitochondrial dysfunction through immunofluorescence and mitochondrial assays. This dysfunction led to mitochondrial DNA release and subsequent activation of the cGAS-STING-IFNB1 pathway, driving M1-like polarization of TAMs. Flow cytometry demonstrated that P2X4R-expressing TAMs not only enhanced CD8^+^ T cell survival and cytotoxicity *in vitro* but also augmented T cell responses in a syngeneic CRC mouse model. Clinically, reduced P2X4 expression in CRC tissues correlated with poorer prognosis. In conclusion, these findings identify the P2X4R as a key regulator of M1-like TAM polarization, representing a promising target to reprogram TAMs and suppress CRC progression.

## Highlights

• P2X4 promotes M1-like macrophage polarization via mtDNA release and cGAS-STING activation
• P2X4^+^ TAMs enhance CD8^+^ T cell survival and cytotoxicity in CRC models
• Low P2X4 expression in CRC correlates with poor patient prognosis

## Graphical abstract

**Figure:**
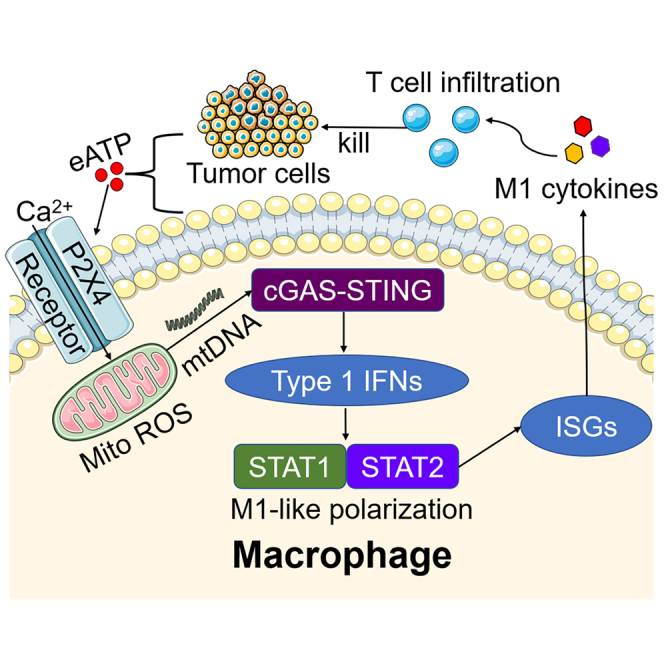

## Introduction

Colorectal cancer (CRC) remains a leading cause of cancer-related mortality globally, with the tumor microenvironment (TME) playing a pivotal role in its progression and therapeutic resistance.^1^ Tumor-associated macrophages (TAMs), as central components of the TME, display functional plasticity that influences cancer outcomes.^2^ In contrast to many other tumor types, where high TAM infiltration correlates with poor prognosis, CRC shows a paradoxical association between TAM abundance and improved survival, indicating subtype-specific immune modulation.^3^

TAMs can exhibit phenotypes ranging from pro-inflammatory (M1-like) to immunosuppressive (M2-like) states, influenced by dynamic environmental cues. Promoting M1-like polarization of TAMs has emerged as a promising strategy to enhance anti-tumor immunity.^2^ However, mechanisms governing this polarization, especially in CRC, remain inadequately defined.

Among potential modulators, purinergic signaling via extracellular ATP (eATP) and its receptors has garnered attention for regulating immune responses.^4^ The P2 receptor family, particularly ionotropic P2X receptors, mediates calcium influx upon ATP binding and influence macrophage function. While P2X7 has been extensively studied in inflammasome activation,^5^ the role of P2X4, another ATP-gated ion channel highly expressed in macrophages, is gaining recognition for its immunomodulatory potential. P2X4 activation can promote macrophage cytokine release, chemotaxis, and inflammatory responses.^6^ In the context of sepsis, the P2X4 receptor has been shown to augment the immune response and antibacterial activity of macrophages stimulated by eATP.^7^ In a mouse model of endometriosis, oral administration of a P2X4 antagonist significantly reduced macrophage infiltration and downregulated local inflammatory mediators, such as IL-33 and COX-2.^8^ Additionally, Li et al. reported that blocking P2X4 attenuates liver fibrosis by suppressing classical inflammatory signaling pathways in macrophages.^9^ These findings highlight the receptor’s role in macrophage polarization and immune regulation.

The role of P2X4 in cancer has also garnered increasing interest. However, previous studies have mainly focused on the effects of P2X4 expression on tumor cell behavior.^10^^,^^11^ Recently, Yang et al. reported that silencing P2X4 in TAMs significantly reduced the secretion of IL-1β and IL-18, thereby diminishing their pro-invasive effects on glioma cells,^12^ suggesting that P2X4-mediated purinergic signaling plays a critical role in regulating TAM function and tumor immunity.

Despite these insights, the role of P2X4 in regulating TAM polarization in CRC and its impact on T cell responses remains unclear. This study investigates whether P2X4 signaling promotes M1-like TAM polarization in CRC and how this influences cytotoxic T cell infiltration and function. Our findings provide new insight into purinergic regulation within the tumor microenvironment and support the potential of targeting P2X4 in CRC immunotherapy.

## Results

### M1-like polarization of CRC cell-associated macrophages depends on activating the cGAS-STING pathway

**Figure 1:**
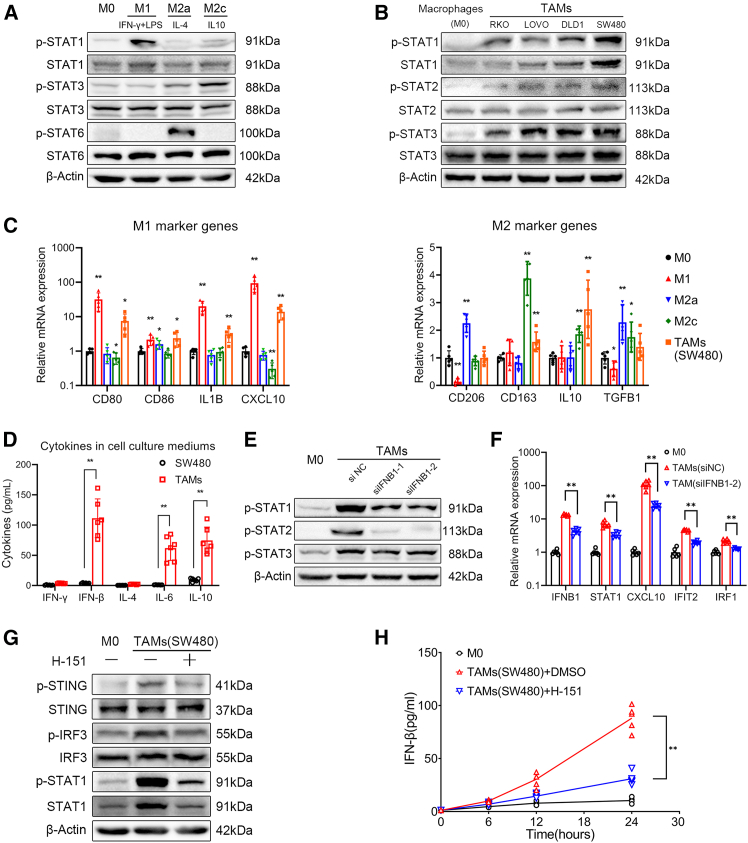
Polarization characteristics and cGAS-STING signaling in CRC cell-associated macrophages (A) THP-1-derived macrophages polarization were induced by IFN-γ + LPS (M1), IL-4 (M2a), or IL-10 (M2c). (B) THP-1-derived macrophages were induced into TAMs by conditioned medium from CRC cell lines RKO, LOVO, DLD1, and SW480. phosphorylation levels of STATs proteins were assessed via western blot after 48 h. (C) Expression of macrophage marker genes were compared with M0 group by qPCR (∗*p* < 0.05, ∗∗*p* < 0.01, Kruskal-Wallis test). (D) ELISA assay was performed to compare the cytokine secretion in 24 h between SW480 and TAMs induced by SW480-conditioned medium (∗∗*p* < 0.01, Student’s t test). (E) Post-transfection with siIFNB1 or control siRNA, THP-1-derived macrophages were induced into TAMs and phosphorylation levels of STATs proteins were visualized via western blot. (F) Changes in mRNA levels of IFNB1 and ISGs were assessed via qPCR (∗∗*p* < 0.01, Kruskal-Wallis test). (G) During TAM induction, 10 μmol/L H-151 was added to block STING, the phosphorylation of proteins was assessed via western blot. (H) IFN-β secretion was quantified by ELISA (∗∗*p* < 0.01, One-way ANOVA). All data are presented as mean ± SD. Data points represent independent biological replicates. Western blots images shown are representative of 3 independent experiments.

The polarization of TAMs determines their pro-tumorigenic or anti-tumorigenic functions within TME.^2^ The STAT protein family, which mediates extracellular-to-intracellular signaling, is regarded as a crucial connection in the process of the macrophage polarization. Activation of the STAT1 pathway is characteristic of M1-type polarization, whereas activation of STAT3 or STAT6 pathways is more prevalent in M2-type polarization^13^ (Figure 1A). Previous studies showed that conditioned medium from CRC cells can induce the differentiation of THP-1 cells into a population of macrophages exhibiting characteristics typical of TAMs.^14^ Upon incubation of THP-1-derived macrophages in the conditioned medium from various CRC cell lines, differential levels of phosphorylation in STAT1, STAT2, and STAT3 proteins were observed (Figure 1B). Comparative analysis of M1 and M2 marker gene expression among M1, M2, and CRC cell-conditioned medium induced macrophages revealed a notable upregulation of both M1 marker genes (e.g., IL1B, CXCL10, and CD80) and M2 marker genes (e.g., IL10 and CD163) in macrophages treated with the supernatant from the CRC cell line SW480 (Figure 1C). This suggests that, similar to TAMs *in vivo*, the macrophages induced by conditioned medium from CRC cells (Hereafter referred to as CRC cell-associated macrophages) exhibit both M1-like and M2-like phenotypes and can serve as an *in vitro* model of TAMs.^2^

From where did the cytokines that induced M1-like polarization in macrophages associated with colorectal cancer (CRC) cells originate? Upon comparing the levels of cytokines associated with STATs protein phosphorylation in the supernatant of the CRC cell line SW480 and TAMs induced by SW480-conditioned medium, cytokine secretion was significantly higher in TAMs, with the STAT1/STAT2 activator IFN-β being one of them. (Figure 1D). The knockdown of IFNB1 in THP-1 derived macrophages via small interfering RNA (siRNA) led to a significant inhibition of the phosphorylation of STAT1 and STAT2 proteins. (Figure 1E). The expression of interferon-stimulated genes (ISGs),^15^ including STAT1, CXCL10, IRF1, and IFIT2, which are highly expressed in TAMs, was also significantly downregulated (Figure 1F). These findings indicated that the IFN-β autocrine in CRC cell-associated macrophages is associated with activation of the STAT1 pathway and M1-like polarization.

The cGAS-STING pathway represents one of the crucial pathways for the upregulation of IFNB1 expression. Western blot analysis showed phosphorylation of cGAS-STING pathway-related proteins such as STING, IRF-3 and STAT1 in TAMs induced by SW480-conditioned medium. This phosphorylation could be effectively inhibited by the STING blocker H-151 (Figure 1G). Furthermore, H-151 also significantly reduced the secretion of IFN-β in TAMs (Figure 1H), suggesting that the cGAS-STING pathway is activated during the polarization of CRC cell-associated macrophages.

### P2X4 receptor regulates cGAS-STING pathway activation in CRC cell-associated macrophages

**Figure 2:**
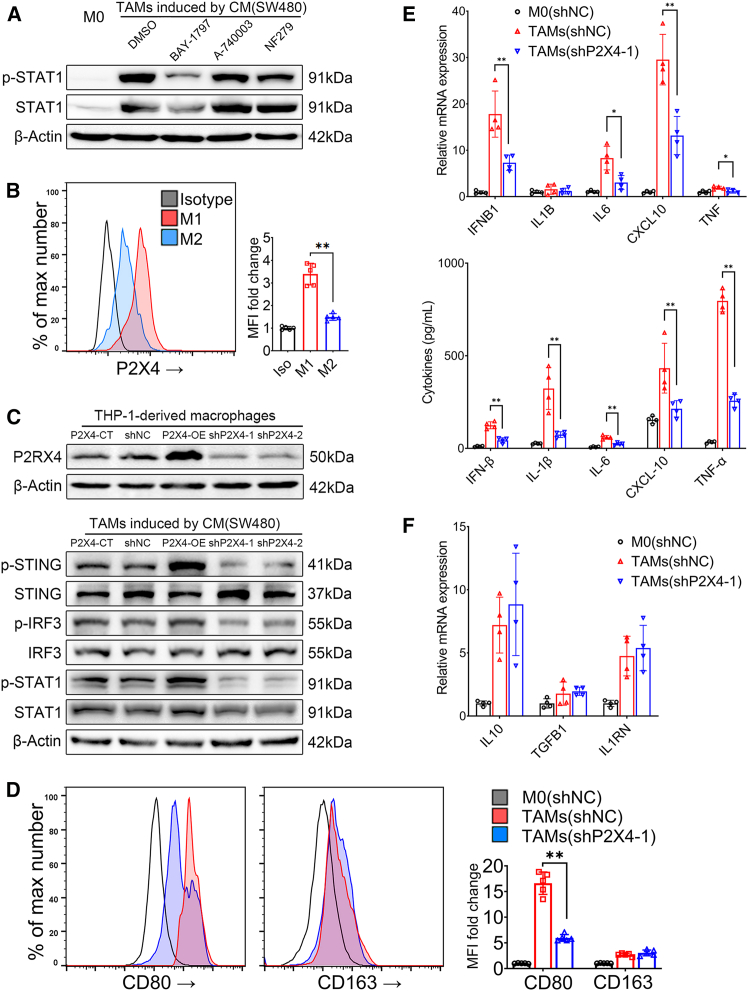
Effect of P2X4 receptors in TAMs on the cGAS-STING pathway and mtDNA release (A) PBMC-derived macrophages were induced into TAMs by SW480-conditioned medium with the addition of 20 μmol/L BAY-1797 (P2X4 blocker), 20 μmol/L A-740003 (P2X7 blocker), and 20 μmol/L NF279 (P2X1 blocker), respectively. An equal final concentration of DMSO (0.05%) was used including vehicle controls. The STAT1 phosphorylation were observed by western blot. (B) P2X4 receptor expression in M1/M2 macrophages was observed by cytometry. Median fluorescence intensity (MFI) was compared (∗∗*p* < 0.01, One-way ANOVA). (C) Lentiviral transfection was performed to construct P2X4 overexpression or knockdown stable cell lines of THP-1 cells, the expression of P2X4 protein was verified. The differences in protein phosphorylation were compared among TAMs derived from each stable cell line by western blotting. (D) The surface markers of macrophage (CD80-APC, CD163-PE) were compared by cytometry (∗∗*p* < 0.01, One-way ANOVA). (E) The mRNA levels of M1 marker genes and cytokine secretion levels were compared via qPCR and ELISA (∗∗*p* < 0.01, Kruskal-Wallis test). (F) The mRNA levels of M2 marker genes were compared via qPCR (Kruskal-Wallis test). All data are presented as mean ± SD. Data points represent independent biological replicates. Western blots images shown are representative of 3 independent experiments.

Purinergic receptors are responsible for mediating extracellular nucleotide signaling, and the expression profiles of their subtypes are correlated with the immune function of macrophages.^4^ The mechanism through which the P2X receptor family regulates TAMs polarization remains unclear. Human peripheral blood mononuclear cells (PBMCs) were differentiated into macrophages, followed by the induction of M1 or M2 polarization (Figures S1A–S1C). It was found that the P2X1, P2X4, and P2X7 receptors are highly expressed on macrophages (Figures S1D and S1E). Blocking the P2X4 receptor significantly reduced both the expression and phosphorylation levels of STAT1 protein when intervening with different P2X receptor inhibitors (P2X4 blocker BAY-1797, P2X7 blocker A-740003, P2X1 blocker NF279) in the induction of macrophages into TAMs by SW480-conditioned medium (Figure 2A). Additionally, the expression of the P2X4 receptor in M1 type macrophages is observed to be higher compared to that in M2 type (Figure 2B), suggesting a potential association between the P2X4 receptor and M1-like polarization of TAMs.

To observe the role of the P2X4 receptor in the polarization of TAMs, stable cell lines of THP-1 cells overexpressing P2X4 (P2X4-OE) and knockdown of P2X4 (shP2X4), along with their respective control (P2X4-CT, shNC), were constructed (Figure 2C). Macrophages from each group were induced into TAMs by SW480-conditioned medium. Western blot analysis showed that the phosphorylation levels of STING, IRF3, and STAT1 proteins were significantly lower in the shP2X4 group compared to the shNC control group. Conversely, these phosphorylation levels were elevated in the P2X4-OE group compared to the P2X4-CT control group (Figure 2C). Simultaneously, in comparison with the shNC control group, the expression of M1-macrophage surface marker CD80 and M1-related cytokines such as IFNB1, CXCL10, TNF, and IL-6 in the shP2X4 group was downregulated (Figures 2D and 2E). Meanwhile, the expression levels of M2 genes showed no significant change (Fig. F). These findings indicate the key role of the P2X4 receptor in activating the cGAS-STING pathway and promoting the expression of M1-related genes in TAMs. However, the upstream signals mediating P2X4-dependent STING activation remain unclear.

### P2X4 receptor activates the mtDNA-cGAS-STING axis via calcium influx and cytosolic mtDNA release in macrophages

**Figure 3:**
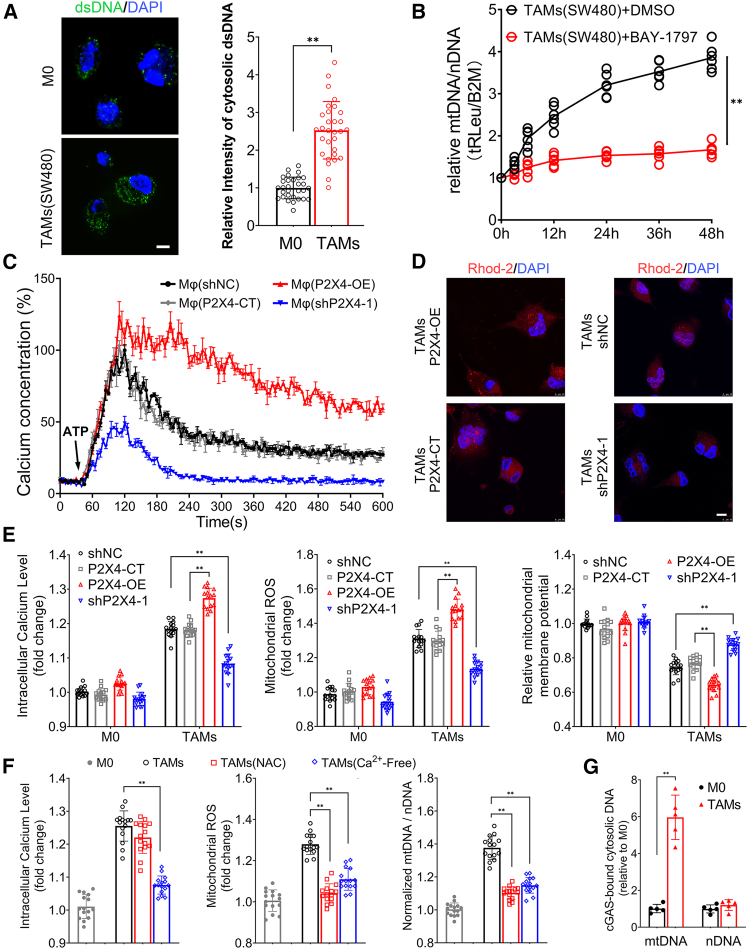
Mechanism of mitochondrial calcium overload by P2X4 receptor-mediated calcium influx in TAMs (A) TAMs were induced from THP-1-derived macrophages. Cytosolic dsDNA was detected using an anti-dsDNA antibody with Alexa Fluor 488 (green), and nuclei were counterstained with DAPI (blue). Images were acquired by confocal microscopy (scale bars, 10 μm). Representative image shown (*N* = 3 independent experiments, 10 images were analyzed per experiment). The boxplot shows the signal intensity (∗∗*p* < 0.01, Student’s t test). (B) BAY-1797 was added during induction of TAMs. The trends of mtDNA/nDNA changes in the cytoplasm of cells were compared by detecting reference genes via qPCR (∗∗*p* < 0.01, Student’s t test). (C) After incubation THP-1-derived macrophages from P2X4-OE, shP2X4, and control groups with the Ca^2+^ probe Rhod-2, the magnitude of Ca^2+^ concentration fluctuations in response to 100 μM eATP stimulation was measured on a fluorometric plate reader. (D) The THP-1 stable cell lines were induced into TAMs and Ca^2+^ fluorescence (scale bars, 10 μm) was observed under a confocal microscope (*N* = 3 independent experiments). (E) THP-1-derived macrophages (5 biological samples per group) were loaded with Rhod-2 probe, mitoSOX probe or JC-1 probe, respectively. After 3 h of induction by adding SW480-conditioned medium, intracellular Ca^2+^ concentration, mitochondrial ROS levels and mitochondrial membrane potential changes were detected. All experiments were independently performed three times. (F) During induction of TAM, simultaneous NAC intervention was set up or calcium-free SW480-conditioned medium was used. Intracellular Ca^2+^ concentration and mitochondrial ROS levels were detected. Differences in the mtDNA/nDNA ratio in the cytoplasm were compared (∗*p* < 0.05, ∗∗*p* < 0.01, One-way ANOVA). (G) After TAM induction from THP-1-derived macrophages stably overexpressing FLAG-cGAS, cGAS ChIP was performed and mtDNA/nDNA enrichment was compared by detecting reference genes via qPCR (∗∗*p* < 0.01, Student’s t test). All data are presented as mean ± SD. Data points represent independent biological replicates.

Apart from pathogen-derived double-stranded DNA (dsDNA), certain pathological conditions can lead to the release of damaged cellular nuclear DNA (nDNA) or mitochondrial DNA (mtDNA) into the cytoplasm, activating the cGAS-STING pathway.^16^ To directly visualize cytosolic DNA accumulation in TAMs, we performed immunofluorescence staining using a dsDNA-specific antibody. Following TAM induction, macrophages exhibited a markedly increased cytosolic dsDNA signal (Figure 3A). To explore the source of cytosolic DNA responsible for activating cGAS downstream of P2X4, we examined which type of DNA was preferentially released. Both mtDNA and nDNA were detected in the cytosol of macrophages using primers targeting specific reference genes. Interestingly, the ratio of mtDNA to nDNA increased markedly during TAM induction by SW480-conditioned medium. This elevation was significantly attenuated by the P2X4 antagonist BAY-1797, suggesting that P2X4 receptor activity selectively promotes mtDNA release in TAMs (Figure 3B).

The P2X4 receptor, a member of the ligand-gated ion channel family, regulates calcium influx in macrophages by sensing eATP signals.^6^ Using a calcium ion fluorescent probe, it was observed that the fluctuations in calcium ion concentration in P2X4-OE macrophages were elevated in response to eATP stimulation, whereas diminished responses were observed in shP2X4 macrophages (Figure 3C). Induction of THP-1 stable cell lines into TAMs revealed that the red fluorescence of calcium ions in the cytoplasm and mitochondria was enhanced in P2X4-OE TAMs, whereas it was markedly attenuated in shP2X4 TAMs (Figure 3D).

These observations prompted us to investigate whether P2X4-mediated calcium influx leads to mitochondrial dysfunction and subsequent mtDNA release. Indeed, further experiments revealed that P2X4-OE TAMs exhibited increased intracellular calcium levels, elevated mitochondrial ROS production, and reduced mitochondrial membrane potential, while shP2X4 TAMs showed the opposite pattern (Figure 3E). To validate the hypothesis that calcium overload triggered by P2X4 activation induces mitochondrial ROS production and membrane damage, leading to the cytosolic release of mtDNA, we applied the ROS scavenger N-acetyl-cysteine (NAC) or substitution with calcium-free CRC cell conditioned medium during TAM induction, Both interventions effectively suppressed mitochondrial ROS levels and mtDNA leakage (Figure 3F).

Given that cytosolic mtDNA is a well-established activator of cGAS,^17^ its release upon P2X4 activation may be responsible for initiating the cGAS-STING response observed in TAMs induced by SW480-conditioned medium. To confirm this, we generated a THP-1 cell line stably overexpressing FLAG-tagged cGAS. Following TAM induction, cGAS chromatin immunoprecipitation (ChIP) analysis revealed a significant enrichment of cGAS-bound mtDNA, whereas nuclear DNA binding remained unchanged (Figure 3G). These findings indicate that cytosolic mtDNA accumulation, driven by P2X4 activation, serves as the principal trigger of cGAS-STING pathway activation in TAMs.

### P2X4-dependent M1-like phenotype of TAMs enhance CD8^+^ T cell responses

**Figure 4:**
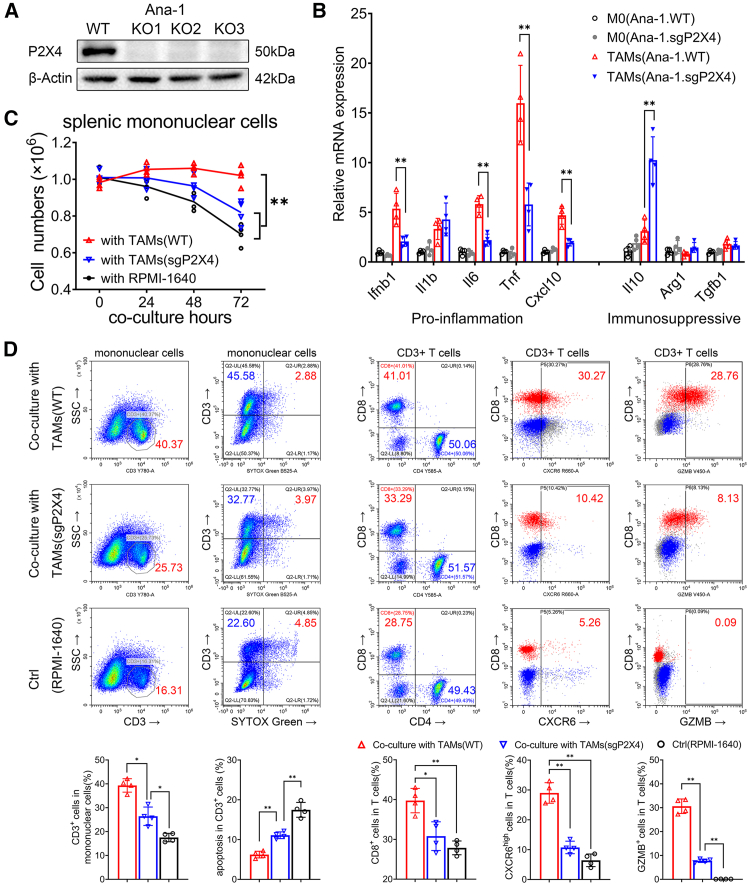
Effects of P2X4 knockout on the polarization of TAMs and T cell regulation (A) P2X4 knockout Ana-1 cell lines were constructed. western blot detected P2X4 expression in three of the monoclonal cell lines. Western blots shown are representative of 3 independent experiments. (B) Wild type or P2X4-K.O. Ana-1 cells were induced into TAMs by MC38 conditioned medium. The mRNA expression of M1/M2 cytokines were compared by qPCR 48 h later (∗*p* < 0.05, ∗∗*p* < 0.01, One-way ANOVA). (C) Suspension mononuclear cells isolated from the spleen of C57BL/6J mice were grouped to be co-cultured with TAMs (sgP2X4) or TAMs (WT). Living cells were counted using a cell viability analyzer at time points (∗∗*p* < 0.01, Student’s t test). (D) After 72 h, the suspension cells were harvested and stained with CD3 (PE-Cy7), CD4 (PE), CD8 (FITC), and CXCR6 (APC) for flow cytometry. Another set of cells were fixed for GzmB (BV421) staining after stimulated with a combination of 50 ng/mL PMA, 1 μg/mL ionomycin, and 5 μg/mL brefeldin A for 4 h. Data were compared by histogram (∗*p* < 0.05, ∗∗*p* < 0.01, Student’s t test). All data are presented as mean ± SD. Data points represent independent biological replicates.

The Ana-1 cell line, originating from C57BL/6 mice, represents a mononuclear macrophage lineage. Utilizing CRISPR double nickase plasmids, we successfully established a P2X4 gene knockout monoclonal cell line (Figure 4A). Subsequently, the Ana-1 macrophages were induced to transition into TAMs through exposure to conditioned medium derived from homologous colon cancer cells, MC38, of C57BL/6 mice. qPCR analysis revealed that TAMs originating from the knockout strain (Ana-1.sgP2X4) demonstrated significantly reduced expression of pro-inflammatory genes compared to their wild-type strain (Ana-1.WT). Conversely, the expression of immunosuppressive genes remained unaffected or was elevated (Figure 4B). These findings implicate that the deletion of the P2X4 gene hinders the development of M1-like phenotypic traits in TAMs. Subsequently, our interest shifted toward examining the impact of M1-like TAMs on the survival and activation of T cells.

To explore the distinct impact of various TAM group on T cells, splenic mononuclear cells (mainly lymphocytes) from C57BL/6J mice were co-cultured with TAMs derived from Ana-1.WT or Ana-1.sgP2X4 for a duration of 72 h. Since T cells cultivated in a laboratory setting necessitate supplementary stimulatory elements for sustained viability, we included a control group in which splenic mononuclear cells were cultured alone in RPMI-1640 medium. As expected, these cells exhibited progressive spontaneous apoptosis over time. In contrast, co-culture with TAMs significantly improved lymphocyte survival, as demonstrated by living cell counting and SYTOX Green staining (Figures 4C and 4D). Flow cytometry analysis further indicated a notably elevated proportion of CD3^+^ T cells among lymphocytes co-cultured with TAMs compared to those cultured in isolation. This enhancement was particularly pronounced within the CD8-positive subpopulation, whereas no significant alteration was observed in the CD4-positive subpopulation (Figure 4D). These findings imply a substantial improvement in the survival of T cells, specifically CD8^+^ T cells, within the co-culture group. However, upon the knockout of the P2X4 receptor in TAMs, the beneficial effect on T cell survival was diminished.

CXCR6 is predominantly expressed on cytotoxic T lymphocytes and plays a key role in supporting their aggregation and survival within the tumor microenvironment, thereby enhancing antitumor immunity.^18^ Interestingly, we observed a significant upregulation of CXCR6 expression on T cells co-cultured with TAMs, particularly among CD8^+^ T cells (red populations) compared to CD4^+^ T cells (blue populations) (Figure 4D). In line with this, CD8^+^ T cells within the co-culture group exhibited the highest level of granzyme B (GzmB) expression when stimulated by a combination of PMA and ionomycin. This finding verifies that M1-like TAMs promote the cytotoxic activity of CD8^+^ T cells. However, this effect was diminished in P2X4-deficient TAMs (Figure 4D). Similar results were also observed in co-cultures with purified splenic T cells, providing further evidence that the functional modulation of T cells is directly mediated by TAMs (Figure S3).

### Influence of P2X4 receptor on T cell infiltration in syngeneic CRC tumors

To evaluate the impact of TAM-expressed P2X4 receptor on T cell infiltration within TME, we established a subcutaneous syngeneic CRC model utilizing C57BL/6J mice, which were divided into three groups. The first group was co-inoculated with MC38 CRC cells and Ana-1.WT macrophages. The second group received a combination of MC38 cells and Ana-1.sgP2X4 cells. Lastly, the third group was inoculated with MC38 cells alone, serving as a control group.

**Figure 5:**
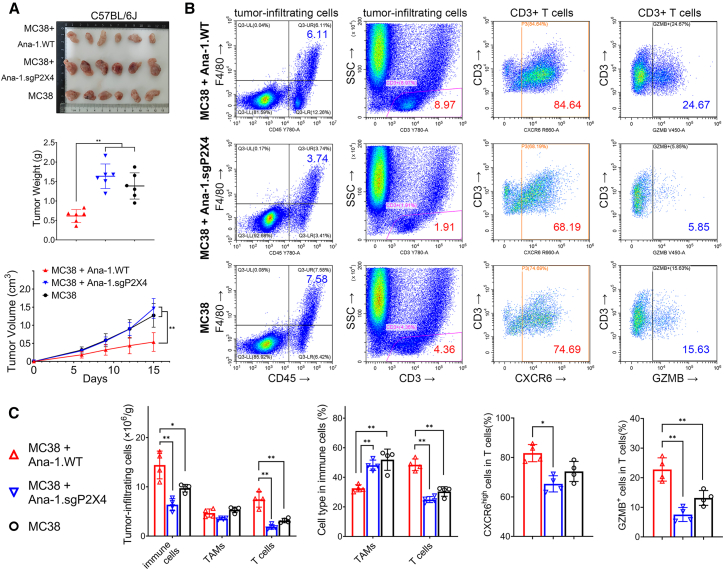
Effect of P2X4 knockout macrophages on T cell infiltration in MC38 tumors in mice (A) 6-week C57BL/6J female mice were divided into 3 groups and inoculated as described. Tumor volume and weight were compared (∗*p* < 0.05, ∗∗*p* < 0.01, Student’s t test). (B) Subcutaneous tumors in each group (4 per group, randomly selected) were digested into single-cell suspensions and stained with antibodies against CD45 (PE-Cy7), F4/80 (AF647), CD80 (PE-CF594), and CD86 (PE) for macrophages, or with antibodies against CD3 (PE-Cy7), CD4 (PE), CD8 (FITC), CXCR6 (APC) and GzmB (BV421) for T cells before flow cytometry. (C) The total number of tumor infiltrating immune cells was measured and the proportions of TAMs and T cells in each group were compared by histogram. (∗*p* < 0.05, ∗∗*p* < 0.01, Student’s t test). All data are presented as mean ± SD. Data points represent independent biological replicates.

Upon comparing the subcutaneous tumor growth curves and final tumor weight across the three groups, it was observed that the tumors in the first group (MC38 + Ana-1.WT) were notably smaller than those in the other two groups. No significant difference was detected between the second group (MC38 + Ana-1.sgP2X4) and the third group (MC38 cells alone) (Figure 5A). This suggests that the wild-type macrophages Ana-1 exhibit a substantial inhibitory effect on the growth of MC38 tumors in mice. However, this inhibitory effect diminishes upon the knockout of the P2X4 gene.

Cells with a density lower than 1.081, mainly lymphocytes, monocytes, and certain stromal cells, were separated through gradient centrifugation for subsequent flow cytometry analysis (Figures 5B and S3A). The results indicated that the first group (MC38 + Ana-1.WT) exhibited the highest infiltration of total immune cells (CD45-positive) (Figure 5C). Specifically, this group demonstrated a significantly higher proportion of infiltrating CD3^+^ T cells compared to the other two groups (Figures 5B and 5C). Similarly, the presence of M1-like TAMs was also notably elevated in this group (Figure S3B). Although not statistically significant, there was a trend toward a higher proportion of CD8^+^ T cells (Figure S3B). These findings imply that the co-inoculated Ana-1 macrophages possess the capability to augment T cell infiltration in a manner dependent on the P2X4 receptor.

To assess the degree of T cell activation, we compared the CXCR6 receptor across all groups. Our findings revealed that the first group exhibited the highest proportion of T cells with elevated CXCR6 expression. Notably, the knockout of P2X4 in macrophages led to a substantial decrease in the proportion of these effector-like T cells. A similar trend was evident in the GzmB-positive T cells across all groups (Figures 5B and 5C).

These findings suggest that co-inoculation of macrophages expressing the P2X4 receptor has the capability to promote increased T cell infiltration within MC38 tumors, thereby impeding tumor progression. This underscores the significance of the P2X4 receptor in facilitating macrophage polarization toward an M1-like TAM phenotype and activating anti-tumor immune responses. Furthermore, comparative analyses conducted in BALB/c-nu mice co-inoculated with MC38 along with either Ana-1.WT or Ana-1.sgP2X4 cells revealed no appreciable difference in tumor growth rates. This observation implies that the immunomodulatory effects exerted by the macrophage P2X4 receptor are contingent upon the presence and function of T cells (Figure S3C).

### Low expression of the P2X4 receptor in colorectal cancer correlates with poor clinical outcome

**Figure 6:**
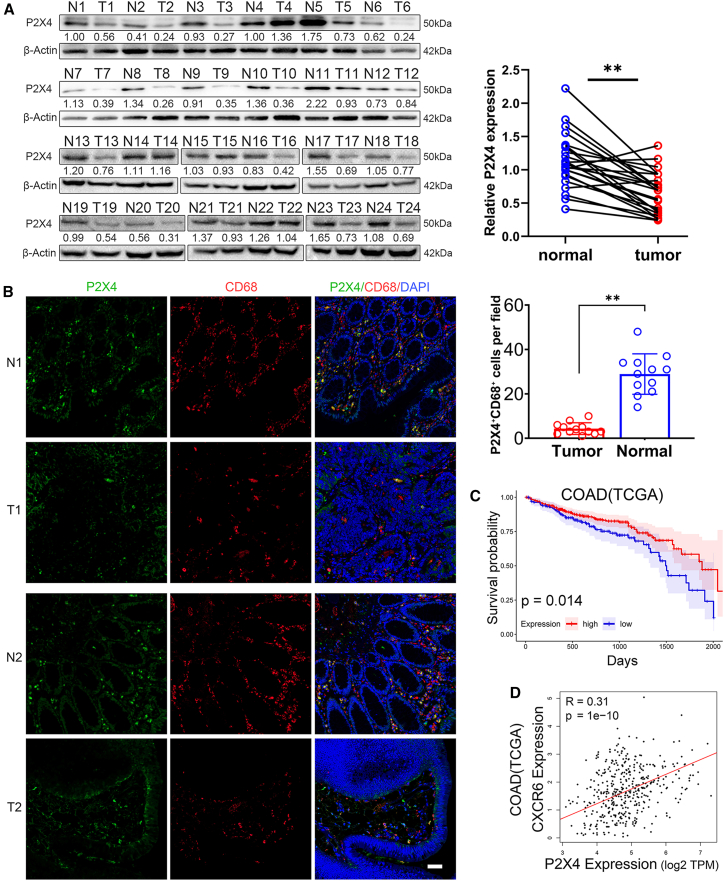
Characterization of P2X4 receptor expression and prognosis in CRC (A) P2X4 protein expression in tumor tissue (T) and adjacent normal tissue (N) from 24 CRC patients was detected by western blot. Relative P2X4 expression were quantified by densitometry and compared in a paired dot plot (∗∗*p* < 0.01, paired Student’s t test). (B) Immunofluorescence staining (P2X4-AF488, CD68-AF594) was performed and observed by confocal microscopy (scale bars, 50 μm). Representative images shown from paired CRC and adjacent tissues (*n* = 12 patients). Boxplot below indicates the number of P2X4^+^CD68^+^ cells per field of view (∗∗*p* < 0.01, Student’s t test). (C) 457 COAD patients in the TCGA database were divided into P2X4 high and low expression groups using FPKM = 3.67 as a cut-off, and overall survival was compared (*p* = 0.014, Log rank test). (D) Correlation between P2X4 and CXCR6 expression in 623 CRC patients from TCGA database (R = 0.31, *p* < 0.01, Spearman Analysis). Data are presented as mean ± SD. Each dot represents one individual patient sample.

To verify the correlation between P2X4 receptor expression and the prognosis of colorectal cancer, we conducted an examination of P2X4 protein expression using clinical samples obtained from 24 patients diagnosed with colorectal cancer. Our findings revealed that in the majority of patients, the expression level of P2X4 was notably reduced in tumor tissues compared to adjacent normal tissues (Figure 6A). Additionally, immunofluorescence analysis confirmed that the number of cells expressing the P2X4 receptor was significantly diminished in tumor tissues relative to adjacent tissues. A majority of the cells exhibiting high expression of the P2X4 receptor were positive for CD68, a marker specific to macrophages (Figure 6B). These results suggest that P2X4 receptor is highly expressed on macrophages within colorectal tissues, and its expression level in tumor tissues is decreased compared to adjacent normal tissues.

Upon analyzing gene expression data from 457 colon adenocarcinoma (COAD) patients and 166 rectal adenocarcinoma (READ) patients within the TCGA cancer genome database, as well as 4 clinical studies on CRC from the GEO database, it was evident that P2X4 gene expression is notably downregulated in tumor tissues compared to normal tissues (Figure S4A). Furthermore, survival curve analysis conducted among COAD patients from TCGA revealed a significant disparity in the five-year survival rates between the P2X4 low-expression and high-expression groups. Specifically, the low-expression group exhibited a five-year survival rate of 51%, which was considerably lower than the 68% observed in the high-expression group (*p* = 0.014) (Figure 6C). These findings suggest that reduced expression of P2X4 in CRC is correlated with an unfavorable prognosis.

The expression of P2X4 exhibited a correlation with the infiltration of M1-type macrophage and T cell in the TCGA CRC dataset (Figures S4B and S4C). More significantly, P2X4 demonstrated a notable association with CXCR6 (Figure 6D), a marker that is distinctly overexpressed on effector-like T cells. This finding underscores the interplay between P2X4 and T cell infiltration within the microenvironment of CRC.

## Discussion

TAMs not only reshape the TME by secreting immunosuppressive factors, resulting in T cell exhaustion and immune evasion of tumor cells but also have the potential to trigger anti-tumor immunity in specific circumstances. Indeed, the beneficial effects exerted by M1-like TAMs have garnered significant attention in various studies. Martin et al. discovered that lung cancer patients harboring M1-like TAMs exhibited greater T cell infiltration and a more favorable prognosis, a mechanism attributed to the recruitment of T cells via CXCL9.^19^ Tkach et al. observed that extracellular vesicles (EVs) released by triple-negative breast cancer (TNBC) cells induced a pro-inflammatory phenotype in certain macrophages, thereby enhancing T cell infiltration and prolonging patient survival.^20^ These observations highlight the pivotal role played by TAMs in reshaping the microenvironment of tumors.

In our study, co-culture experiments revealed that TAMs significantly increased the survival of splenic lymphocytes and augmented the proportion of effector-like T cells. This supportive effect of TAMs on T cells is likely driven by P2X4-dependent M1-like polarization, accompanied by the secretion of chemokines such as CXCL9, CXCL10, and CXCL16, as well as enhanced antigen-presenting functions. Furthermore, in a syngeneic CRC mouse model, we observed enhanced T cell infiltration into MC38 tumors mediated by wild-type Ana-1 macrophages, corroborating the mechanisms through which M1-like TAMs enhance adaptive immunity. Recent studies have highlighted the activation of the CXCL16/CXCR6 axis as a pivotal element in T cell-mediated tumor immunity,^21^ which is consistent with the increased proportion of CXCR6-high T cells we detected in interactions with M1-like TAMs.

The polarization of macrophages within tumor tissues exhibits remarkable complexity. In this study, we uncovered that, under the stimulus provided by CRC cells, macrophages can undergo M1-like polarization through the autocrine secretion of IFN-β *in vitro*. Well-documented pathways responsible for activating IFN-β expression encompass TLR/TRIF, TLR/MYD88, RIGI/MD5, and the cGAS-STING pathway. The latter pathway serves as a pivotal element of the innate immune response, triggered by the presence of cytoplasmic double-stranded DNA (dsDNA) to initiate protective measures against microbial invaders and cellular damage. Scholarly works indicate that the cGAS-STING pathway has the capacity to modulate diverse immune cell populations residing within the TME, including macrophages, DCs, and T cells.^22^ However, the source of dsDNA that interacts with cGAS can vary. Frequently, genomic instability in tumor cells gives rise to the formation of micronuclei harboring fragmented DNA. When these micronuclei rupture, they release genomic DNA into the cytoplasm, which has the potential to activate the cGAS-STING pathway. This particular mechanism has been substantiated through *in vitro* experiments^23^ and radiotherapy-based animal models.^24^

In addition to the genomic DNA derived from tumor cells acting as a ligand, our study provides the first account of how macrophages, under the induction of tumor cells, can trigger the cGAS-STING pathway through the release of mtDNA. This finding holds profound importance in examining the intricacies of innate immune activation within the tumor microenvironment. Prior investigations have documented that particular pathways (such as TNF/TNFR1^25^ or IL-1β/IL-1R^26^) are capable of inducing mitochondrial depolarization in macrophages, leading to the release of mtDNA into the cytoplasm and subsequent activation of the cGAS-STING pathway. Nevertheless, the precise mechanisms responsible for the release of mtDNA from mitochondria remain to be further elucidated.

Purinergic receptors, which bind to ligands (eATP and adenosine, etc.) secreted via exocytosis,^5^^,^^27^ play a pivotal role in various cellular physiological functions. Our findings reveal that the calcium influx mediated by the purinergic receptor P2X4 is a critical step for the release of mtDNA in TAMs. Under the influence of tumor cells, activation of the P2X4 receptor leads to elevated intracellular calcium levels, resulting in mitochondrial dysfunction and structural damage.^28^ This, in turn, initiates the release of mtDNA into the cytoplasm, thereby activating the cGAS-STING pathway. eATP, the natural ligand for the P2X4 receptor, which is secreted by stimulated cells as well as released from dead cells, is abundant in both tumor and inflammatory environments. In the present *in vitro* study, it is postulated that eATP may originate from tumor cells or, more likely, from autocrine secretion by macrophages induced by CRC cell-conditioned medium. However, further experiments are required to confirm this hypothesis.

Although the immunoregulatory role of purinergic signaling has been widely investigated in the context of cancer, comparatively fewer studies have addressed the function of P2X4R. In certain tumor cells overexpressing P2X4, such as those found in breast cancer^10^ and prostate cancer,^11^ P2X4 is believed to be associated with the invasive capabilities of tumor cells and epithelial mesenchymal transition. Additionally, it has been suggested that P2X4 may enhance the immunogenic cell death of tumor cells.^29^ However, these studies primarily addressed P2X4R function in tumor cells, with limited insight into its immunomodulatory roles in the TME, particularly in TAM biology.

Our study highlights a distinct facet that in colorectal tissue, P2X4 receptors are predominantly expressed on macrophages and play an integral role in M1-like polarization and immune function of TAMs within the microenvironment. These findings emphasize the tumor-type-specific nature of P2X4 signaling and suggest a unique immunoregulatory role for P2X4 in the CRC microenvironment. Supporting this, both our tissue analysis and TCGA data show reduced P2X4 expression in CRC tumors, which may impair cGAS-STING pathway activation in TAMs and weaken antitumor immunity. Targeted activation of P2X4 on macrophages could help reprogram TAMs toward a pro-inflammatory phenotype and reshape the immunosuppressive TME.

Collectively, our findings provide mechanistic insight into the role of P2X4 in shaping TAM function and offer a rationale for its development as an immunotherapeutic target in colorectal cancer.

### Limitations of the study

However, several limitations in this study should be noted. First, although the study employed both *in vitro* and *in vivo* models, it may not fully replicate the complexity of the tumor microenvironment (TME) in human CRC, stromal fibroblasts, endothelial cells, and other immune cells are also critical components of the TME and may interact with TAMs through broadly expressed purinergic signaling pathways.^4^ For instance, P2X4 mediates the release of prostaglandin E2 (PGE2) from macrophages, which subsequently acts on endothelial cells to increase vascular permeability and indirectly modulate immune cell migration.^30^ In addition, our previous research demonstrated that cross-talk between cancer-associated fibroblasts (CAFs) and TAMs shapes macrophage immune function, underscoring the need for further investigation into the role of P2X4 in mediating these interactions.^31^ Given the dynamic interplay among TME components, the long-term consequences of modulating P2X4 signaling require more extensive *in vivo* validation, particularly in spontaneous or orthotopic CRC models. Besides, in order to reflect the characteristics of the CRC patient population, we did not impose any restrictions based on sex and age when selecting participants. However, further investigation into the influence of these factors on the study outcomes is necessary. Notably, only female mice were used in the animal experiments, and thus the potential impact of sex on macrophage polarization and T cell responses should be taken into account when interpreting these results.

Second, while these findings highlight the therapeutic potential of targeting P2X4 in CRC, several key challenges for clinical application remain. P2X4 is also expressed in non-immune cells, such as neurons, endothelial, and epithelial tissues, raising concerns about off-target effects. Systemic activation may disrupt tissue homeostasis or induce undesired inflammation.^32^ Future strategies may require selective delivery approaches, such as TAM-specific nanoparticles or bispecific antibodies, to achieve localized and safe modulation of P2X4 activity.^33^

## Resource availability

### Lead contact

Further information and requests for reagents should be directed to and would be fulfilled by the lead contact, Xiangjun Meng (meng_xiangjun@yahoo.com).

### Materials availability

This study did not generate new unique reagents.

### Data and code availability

• Additional data used in this study can be accessed via NCBI GEO Database under accession number GSE106582, GSE21510, GSE44076 and GSE28000.
• This study does not report original code.
• Any additional information required to reanalyze the data reported in this paper is available from the lead contact upon request.

## Acknowledgments

This work was supported by Interdisciplinary Program of 10.13039/501100004921Shanghai Jiao Tong University (YG2024ZD11), 10.13039/501100001809National Natural Science Foundation of China (grant no. 82102949, 82203245, and 32071377), Shanghai Sailing Program (20YF1424400 and 22YF1423100).

## Author contributions

X.M. and J.W. designed the study. K.Z., X.Z., Y.L., and L.D. performed the experiments. Y.H. supervised. H.Y. and W.W. analyzed the DATA. K.Z. and X.Z. wrote the article.

## Declaration of interests

The authors declare that they have no conflict of interest.

## STAR★Methods

### Key resources table

**Table:** 

REAGENT or RESOURCE	SOURCE	IDENTIFIER
**Antibodies**		

APC anti-human CD80	Biolegend (America)	Cat# 305220; RRID: AB_2076147
BV605 anti-human CD206	BD (America)	Cat# 569177; RRID: AB_3684843
PE anti-human CD 163	BD (America)	Cat# 556018; RRID: AB_396296
FITC anti-human CD 68	BD (America)	Cat# 562117; RRID: AB_10896283
APC anti-m/h CD11b	Biolegend (America)	Cat# 101211; RRID: AB_312794
FITC anti-human P2X4	alomone labs (Israeli)	Cat# APR024-F
PE-Cy7 anti-mouse CD45	BD (America)	Cat# 552848; RRID: AB_394489
AF647 anti-mouse F4/80	BD (America)	Cat# 565854; RRID: AB_2744474
PE-CF594 anti-mouse CD80	BD (America)	Cat# 562504; RRID: AB_2737630
PE anti-mouse CD86	BioLegend (America)	Cat# 105007; RRID: AB_313150
PE-Cy7 anti-mouse CD3e	Biolegend (America)	Cat# 100320; RRID: AB_312685
PE anti-mouse CD4	Biolegend (America)	Cat# 100512; RRID: AB_312715
FITC anti-mouse CD8a	BD (America)	Cat# 553031; RRID: AB_394569
APC anti-mouse CXCR6	Biolegend (America)	Cat# 151106; RRID: AB_2572143
BV421anti-h/m GZMB	BioLegend (America)	Cat# 396414; RRID: AB_2810603
AF594 anti-mouse IgG	Cell Signaling Technology (America)	Cat# 8890S
AF488 anti-rabbit IgG	Cell Signaling Technology (America)	Cat# 4412S
AF488 anti-mouse IgG	Cell Signaling Technology (America)	Cat# 4408S
β-Actin Mouse mAb	Cell Signaling Technology (America)	Cat# 3700S; RRID: AB_2242334
α-Tubulin Mouse mAb	Proteintech (China)	Cat# 66031–1
P2X1 Rabbit pAb	Absin Biotech (China)	Cat# abs102884
P2X4 Rabbit pAb	Absin Biotech (China)	Cat# abs117349
P2X5 Rabbit pAb	Absin Biotech (China)	Cat# abs121706
P2X7 Rabbit pAb	Absin Biotech (China)	Cat# abs133974
CD68 Mouse mAb	ZSGB-Bio (China)	Cat# ZM-0060
dsDNA Mouse mAb	Sigma-Aldrich (America)	Cat# MABE1134
STAT1 Rabbit mAb	Abclonal Biotech (China)	Cat# A19563; RRID: AB_2862669
p-STAT1-Y701 Rabbit mAb	Abclonal Biotech (China)	Cat# AP0054; RRID: AB_2863803
STAT2 Rabbit pAb	Abclonal Biotech (China)	Cat# A14995; RRID: AB_2761878
p-STAT2-Y690 Rabbit pAb	Abclonal Biotech (China)	Cat# AP0284; RRID: AB_2771564
STAT3 Rabbit mAb	Abclonal Biotech (China)	Cat# A22434
p-STAT3-Y705 Rabbit mAb	Abclonal Biotech (China)	Cat# AP0705; RRID: AB_2863810
STAT6 Rabbit pAb	Abcam (England)	Cat# ab32520; RRID: AB_778113
p-STAT6 Rabbit pAb	Abcam (England)	Cat# ab28829; RRID: AB_778116
IRF3 Rabbit pAb	Abclonal Biotech (China)	Cat# A2172; RRID: AB_2764190
*p*-IRF3 Rabbit pAb	Abclonal Biotech (China)	Cat# AP0995; RRID: AB_2863887
STING Rabbit mAb	Abclonal Biotech (China)	Cat# A21051; RRID: AB_3083450
p-STING Rabbit mAb	Abclonal Biotech (China)	Cat# AP1369; RRID: AB_3675875

**Biological samples**		

peripheral blood of healthy human donors	The Ninth People’s Hospital of Shanghai	N/A
tissues samples from CRC patients	The Ninth People’s Hospital of Shanghai	N/A

**Chemicals, peptides, and recombinant proteins**		

NAC	YEASEN Biotech (China)	Cat# 50303ES05
BAY-1797	MedChemExpress (America)	Cat# HY-130605
A-740003	MedChemExpress (America)	Cat# HY-50697
NF279	MedChemExpress (America)	Cat# HY-D0976
H-151	MedChemExpress (America)	Cat# HY-112693
ATP	YEASEN Biotech (China)	Cat# 60605ES03
LPS	Sigma-Aldrich (America)	Cat# L8643
PMA	MedChemExpress (America)	Cat# HY-18739
human M-CSF	Proteintech (China)	Cat# HZ-1192
human IFN-γ	MedChemExpress (America)	Cat# HY-P70610
human IFN-β	MedChemExpress (America)	Cat# HY-P7024
human IL-4	PeproTech (America)	Cat# AF-200-04
human IL-10	PeproTech (America)	Cat# AF-200-10

**Critical commercial assays**		

Lipofectamine 3000	Invitrogen (America)	Cat# L3000-015
Lipofectamine RNAiMAX	Invitrogen (America)	Cat# 13778–075
HISTOPAQUE	Sigma-Aldrich (America)	Cat# 10771
Mouse Lymphocyte Separation Medium	Dakewe Biotech (China)	Cat# DY7211011
Mouse Tumor Cell Isolation Kit	Miltenyi (Germany)	Cat# 130-096-730
Mouse CD3^+^ T cell Isolation Kit	Selleck (America)	Cat# B90021
Anti-FLAG M2 Magnetic Beads	Sigma-Aldrich (America)	Cat# M8823
FLAG peptide	Sigma-Aldrich (America)	Cat# F4799
Fixation Buffer	BioLegend (America)	Cat# 420801
Permeabilization Wash Buffer	BioLegend (America)	Cat# 421002
FastPure EndoFree Plasmid Maxi Kit	Vazyme Biotech (China)	Cat# DC202
TIANamp Genomic DNA Kit	TIANGEN Biotech (China)	Cat# DP304
MolPure Cell RNA Kit	YEASEN Biotech (China)	Cat# 19231ES50
One-step RT-gDNA digestion SuperMix	YEASEN Biotech (China)	Cat# 11142ES60
qPCR SYBR Green Master Mix	YEASEN Biotech (China)	Cat# 11201ES08
Cellular mitochondria isolation kit	Beyotime Biotech (China)	Cat# C3601
Rhod-2/AM Calcium fluorescent probe	YEASEN Biotech (China)	Cat# 40776ES50
MitoSOX Mitochondrial Superoxide Indicator	YEASEN Biotech (China)	Cat# 40778ES50
JC-1 MitoMP Detection Kit	DOJINDO (Japan)	Cat# MT09
YF594 EdU Imaging Kit	YEASEN Biotech (China)	Cat# 40276ES76
Human Interferon Beta ELISA Kit	Abclona Biotech (China)	Cat# RK01630
Human CXCL10 ELISA Kit	Abclona Biotech (China)	Cat# RK00054
Human Cytokine 12-Plex Kit	Abclonal Biotech (China)	Cat# RK04296
Human CD14 MicroBeads	Miltenyi (Germany)	Cat# 130-050-201
DAPI-containing antifade mounting medium	Beyotime Biotech (China)	Cat# P0131

**Deposited data**		

TCGA colorectal cancer data	TCGA Data Portal	https://portal.gdc.cancer.gov
GEO expression profiles (GSE106582, GSE21510, GSE44076, GSE28000)	NCBI GEO Database	https://www.ncbi.nlm.nih.gov/geo/
Immune infiltration analysis	TIMER Database	https://timer.cistrome.org

**Experimental models: Cell lines**		

Human acute monocyte leukemia cell line THP-1	Cell Bank of the Chinese Academy of Sciences	SCSP-567
Human embryonic kidney epithelial cell line HEK293T	Cell Bank of the Chinese Academy of Sciences	SCSP-502
Human colon adenocarcinoma cell line SW480	Cell Bank of the Chinese Academy of Sciences	SCSP-5033
Human colon adenocarcinoma cell line DLD-1	Cell Bank of the Chinese Academy of Sciences	SCSP-5241
Human colon adenocarcinoma cell line RKO	Cell Bank of the Chinese Academy of Sciences	SCSP-5236
Human colon adenocarcinoma cell line LoVo	Cell Bank of the Chinese Academy of Sciences	SCSP-514
Murine macrophage cell line Ana-1 (C57BL/6)	Cell Bank of the Chinese Academy of Sciences	GNM 2
Murine colon carcinoma cell line MC38 (C57BL/6)	Cell Bank of the Chinese Academy of Sciences	SCSP-5431

**Experimental models: Organisms/strains**		

Mouse: C57BL/6J	Shanghai Youshulife Technology Co.	IMSR_JAX:000664
Mouse: BALB/c-nu	Shanghai Youshulife Technology Co.	IMSR_CRL:194

**Oligonucleotides**		

siIFNB1-1: GACCAUAGUCAGAGUGGAAdTdT	This paper	N/A
siIFNB1-2: AGACAGUCCUGGAAGAAAAdTdT	This paper	N/A
shP2X4-1: GGAATATCCTTCCCAACAT	This paper	N/A
shP2X4-2: GTACTACAGAGACCTGGCT	This paper	N/A
Primers: See Table S1 in Supplementary Information	This paper	N/A

**Recombinant DNA**		

P2X4 CRISPR Plasmid (m)	SANTA CRUZ	Cat# sc-422092-NIC
Plasmid: P2X4	Shanghai Generay Biotechnology	N/A
Plasmid: FLAG-cGAS	Shanghai Generay Biotechnology	N/A

**Software and algorithms**		

IBM SPSS Statistics 27	IBM Corp., USA	https://www.ibm.com/products/spss-statistics
FlowJo v10	BD Biosciences	https://www.flowjo.com/
GraphPad Prism 8	GraphPad Software	https://www.graphpad.com/scientific-software/prism/
ImageJ	National Institutes of Health (NIH)	https://imagej.nih.gov/ij/
BioTek Gen5	Agilent Technologies	https://www.agilent.com/en/product/

### Experimental model and study participant details

#### Human tissue samples

A total of 24 frozen tumor tissues and matched adjacent normal tissues were analyzed from the Gastrointestinal Tumor Sample Bank of the Ninth People’s Hospital Affiliated to Shanghai Jiao Tong University School of Medicine. The samples were randomly selected from CRC patients who underwent surgical resection between 2019 and 2024 in the Ninth People’s Hospital. The patients had a mean age of 62 ± 17 years and included 14 males (58.3%) and 10 females (41.7%). The specimens had a diameter of no less than 20 mm. Written informed consent was obtained from each patient, and the study was approved by the Ethics Committee of the Ninth People’s Hospital (approval no. SH9H-2019-T169-1). All experiments conformed to the relevant regulatory standards.

#### Animal models

For syngeneic tumor models, six-week-old female C57BL/6J or BALB/c-nu mice (Shanghai Youshulife Technology Co.) were divided into different treatment groups. 2 × 10^6^ MC38 cells with or without 1×10^6^ Ana-1 cells were subcutaneously injected into the right axilla. Tumor volume was monitored every 3 days. After 15 days, the mice were euthanized and the tumors were harvested. Mice were housed in SPF conditions and all animal procedures were approved by the IACUC (approval no. Ys-m202402001). All institutional and national guidelines for the care and use of laboratory animals were followed.

#### Cells

All cell lines, obtained from the Cell Bank of the Chinese Academy of Sciences (Shanghai, China), were tested for mycoplasma contamination and accompanied by authentication reports. PBMCs were isolated from healthy human donors using Human Blood Lymphocyte Isolation Solution and enriched for CD14^+^ cells via magnetic sorting. Mouse splenic lymphocytes were isolated from male C57BL/6J mice using Mouse Lymphocyte Isolation Solution, and CD3^+^ T cells were purified by magnetic negative selection. PBMC, THP-1 monocytes and human CRC cell lines were maintained in RPMI-1640 medium supplemented with 10% fetal bovine serum (FBS) and antibiotics. HEK293T, Ana-1 mononuclear macrophages, MC38 murine CRC cell line and murine lymphocytes were maintained in DMEM with the same supplements. The cells were cultured in a 37°C water-saturated 5% CO2 atmosphere.

In the co-culture experiment, Ana-1 cells were pretreated with MC38-conditioned medium for 48 h to induce TAM-like polarization. Subsequently, TAMs (0.5×10^6^/well) and splenic lymphocytes (2×10^6^/well) were seeded at optimal densities and co-cultured in 6-well plates for 72 h.

### Method details

#### Stable cell line construction

The shP2X4 plasmids, P2X4 OE plasmids, FLAG-cGAS OE plasmids and their negative control were synthesized and purchased from Shanghai Generay Biotechnology. HEK293T cells were transfected with the packaging plasmids psPAX2, pMD2G and the constructed plasmid using the Lipofectamine 3000. Supernatant was harvested and filtered after 48h. THP-1 cells were infected with the concentrated lentivirus in the presence of 6 μg/mL Polybrene. Cells were selected at 48h post-infection with puromycin.

To construct the P2X4 knockout Ana-1 cell line, Ana-1 cells were transfected with a double-incision enzyme plasmid (sc-422092-NIC, SANTA CRUZ) targeting the mouse P2rx4 gene using Lipofectamine 3000. GFP-positive cells were sorted by flow cytometry and spread one by one into 96-well plates at 48h post-infection. Positive cells were screened by incubating with 2 μg/mL puromycin for 3 days. After monoclonal cell expansion, proteins were extracted and successful knockdown monoclonal cell lines were verified by Western blot.

#### siRNA transfection

Cells were transfected with the small interfering RNAs (siRNAs) or their corresponding negative control (NC) that were synthesized and purchased from Shanghai Zorinbio Technology using Lipofectamine RNAiMAX transfection reagent (Thermo Scientific). The sequences were listed in the key resources table.

#### Macrophage polarization

THP-1 cells were treated with 100 nM Phorbol 12-myristate 13-acetate (PMA) for 48 h, or CD14^+^ PBMCs were cultured with 20 ng/mL M-CSF for 6 days to generate M0 macrophages. Next, macrophages were treated either with 10 ng/mL IFN-γ and 100 ng/mL LPS (to polarize toward M1 phenotype) or with 20 ng/mL IL-4/IL-10 (M2a/M2c) for 48 h. Besides, supernatants were collected from CRC cells after 24 h of culture. TAMs were induced by culturing macrophages with filtered CRC-conditioned medium (1:1 with RPMI) for 48 h.

#### Intracellular calcium, mitochondrial superoxide and membrane potential detection

Cells in 96-well plates were washed with HBSS, and loaded with 5 μmol/L of the working solution (Rhod2/AM, MitoSOX Red or JC-1) and incubate in a 37°C in the dark for 30 min. After treatment, observe the intracellular calcium fluorescence under a laser confocal microscope (IX73, Olympus), or measure the fluorescence intensity (Rhod-2/AM at 549/578 nm, MitoSOX at 510/580 nm, JC-1 at 561/590 nm for red and 488/530 nm for green) using a fluorometric plate reader (SYNERGY H1, BioTek).

#### Cytosolic DNA detection

To detect the ratio of mtDNA to nDNA released into macrophage cytoplasm. Cells were lysed with a cell homogenizer and then centrifuged at 12,000 g, 4°C for 10 min. The supernatant was collected. Extraction of DNA in the cytosol was performed using the Genomic DNA Kit. nuclear DNA primers (B2M) and mtDNA primers (tRLeu) were used in the qPCR measurement of the cytosolic DNA abundance.

#### FLAG-cGAS ChIP

THP-1-derived macrophages stably overexpressing FLAG-tagged cGAS were induced into TAMs using SW480-conditioned medium. After 48 h of induction, cells were crosslinked in 1% formaldehyde for 10 min, quenched with glycine, and snap-frozen. Cells were lysed in SDS lysis buffer, followed by sonication and centrifugation to remove cell debris. The diluted supernatant was incubated with anti-FLAG M2 magnetic beads at 4°C overnight with gentle rotation. Beads were washed sequentially with low salt, high salt, LiCl, and TE buffers. Bound complexes were eluted using 150 μg/mL 1× FLAG peptide and reversed by incubating at 65°C overnight. DNA was purified and analyzed by qPCR using primers specific for mitochondrial DNA (tRLeu) and nuclear DNA (B2M).

#### Western blot analysis

Protein samples were extracted from cells or tissues with RIPA Lysis Buffer as directed. After protein quantification by the BCA assay, equal amounts of protein were mixed with loading buffer and analyzed by SDS-PAGE. Proteins were transferred to PVDF membranes and incubated with primary and secondary antibodies. Immunoblots were revealed using the chemiluminescence reagent and visualized using a luminescence imaging system (Tanon 5200).

#### Cytokine detection in culture supernatants

Cell culture supernatants from SW480 and macrophages were collected at each time point described, centrifuged for 10 min at 1000×g and kept at −20°C for cytokine measurements. The secretion level of IFN-β and CXCL-10 were measured using the Human IFNB or CXCL10 ELISA Kit. For secretion levels of other cytokines (IL-1β, IL-4, IL-6, IL-10, TNF-α and IFN-γ), the ABplex Human Cytokine 12-Plex Assay Kitwas used, following manufacturers’ instructions.

#### Reverse transcription and quantitative-PCR

Total RNA from cells was isolated using the MolPure Cell RNA Kit. RNA was reversed transcribed using the RT-gDNA digestion SuperMix for qPCR, following instructions. Equal amounts of cDNA was amplified using the qPCR SYBR Green Master Mix in the Light Cycler 480II (Roche). Primer sequences are listed in Table S1 mRNA expression was normalized to the levels of GAPDH, a housekeeping gene.

#### Flow cytometry

Xenograft tissues were digested using the Tumor Dissociation Kit according to the instructions. Cells were washed and resuspended with PBS containing 1% BSA, then pre-incubated with Fc Receptor Blocking Solution for 20 min. After fixation or not, stain the cells with appropriate fluorescent antibodies for 30 min at 4°C. Flow analysis was performed using a CytoFLEX S cytometer (Beckman). Flowjo V10 software was used for data analysis. All experiments were repeated three times.

#### Immunofluorescence staining

Cells were seeded on poly-L-lysine–coated coverslips in 24-well plates and cultured until ∼30% confluence. After washing with PBS, cells were fixed with 4% paraformaldehyde for 20 min, permeabilized with 0.2% Triton X-100 for 15 min, and blocked in 3% BSA for 30 min at room temperature. Primary antibodies diluted in blocking buffer were incubated overnight at 4°C. After three PBST washes, Alexa Fluor–conjugated secondary antibodies were added and incubated for 1 h at room temperature in the dark. Coverslips were mounted using antifade medium containing DAPI and imaged using a confocal microscope.

#### Immunohistochemistry (IHC) staining

Colorectal cancer and paraneoplastic tissue were fixed in 4% formaldehyde overnight and embedded in paraffin. Sections of 4 μm thickness were used for IHC staining. Samples were baked and de-paraffinized,then boiled for antigen retrieval in 10 mM sodium citrate buffer (pH 6.0) for 15 min. Sections were pre-treated with 0.5% Triton X-100 for 30 min before blocking with 3% bovine serum albumin in PBS for 30 min. Then followed by primary antibody (diluted 1:200) incubation overnight at 4°C and fluorescent secondary antibody (diluted 1:2000) incubation for 60 min at room temperature. After washing, the sections were sealed with a DAPI-containing antifade mounting medium.

#### Clinical database analysis

Clinical datasets were retrieved from TCGA (https://portal.gdc.cancer.gov) and GEO (https://www.ncbi.nlm.nih.gov/geo/) to analyze differences in gene expression and to plot Kaplan-Meier survival curves. Immune correlation analyses were conducted using the TIMER database (https://timer.cistrome.org).

### Quantification and statistical analysis

Statistical analysis was performed using SPSS 27.0. For normal distribution data with homogeneous variance, differences were assessed using unpaired t-tests or one-way ANOVA followed by Bonferroni’s test. For nonparametric test, the Kruskal–Wallis test with Dunn’s multiple comparison test was used. Correlations were analyzed using Spearman’s analysis. Log rank test was used for Kaplan-Meier survival analysis. *p* < 0.05 was considered statistically significant. Each data point represents an individual sample in the figures, with error bars denoting the standard deviations. All of the data are presented as mean ± SD and the statistical details can be found in the figure legends.

#### Ethical approval

All procedures of human study were approved by the Ethics Committee of the Shanghai Ninth People’s Hospital (approval no. SH9H-2019-T169-1). Written informed consent was obtained from all participants. The animal study was approved by the IACUC from Shanghai Youshulife Technology (approval no. Ys-m202402001). All institutional and national guidelines for the care and use of laboratory animals were followed.

### Additional resources

This work is not part of/involves a clinical trial.

## Supplemental information

Document S1. Figures S1–S4 and Tables S1

Data S1. Original Western blot images
