# Supplementary material for: M1-like macrophages regulate T cell infiltration in colorectal cancer through P2X4 receptor
Source: iScience. 2025 Sep 5;28(10):113517. doi: 10.1016/j.isci.2025.113517 (PMC12478113; doi:10.1016/j.isci.2025.113517)
Supplement: Document S1. Figures S1–S4 and Tables S1 [file mmc1.pdf]

iScience, Volume *28*

Supplemental information

M1-like macrophages regulate T cell infiltration in colorectal cancer through P2X4 receptor

Kun Zhou, Xintian Zhang, Yu Liang, Han Yao, Yichao Hou, Xingming Zhang, Leilei Du, Wenfeng Wang, Jianhua Wang, and Xiangjun Meng

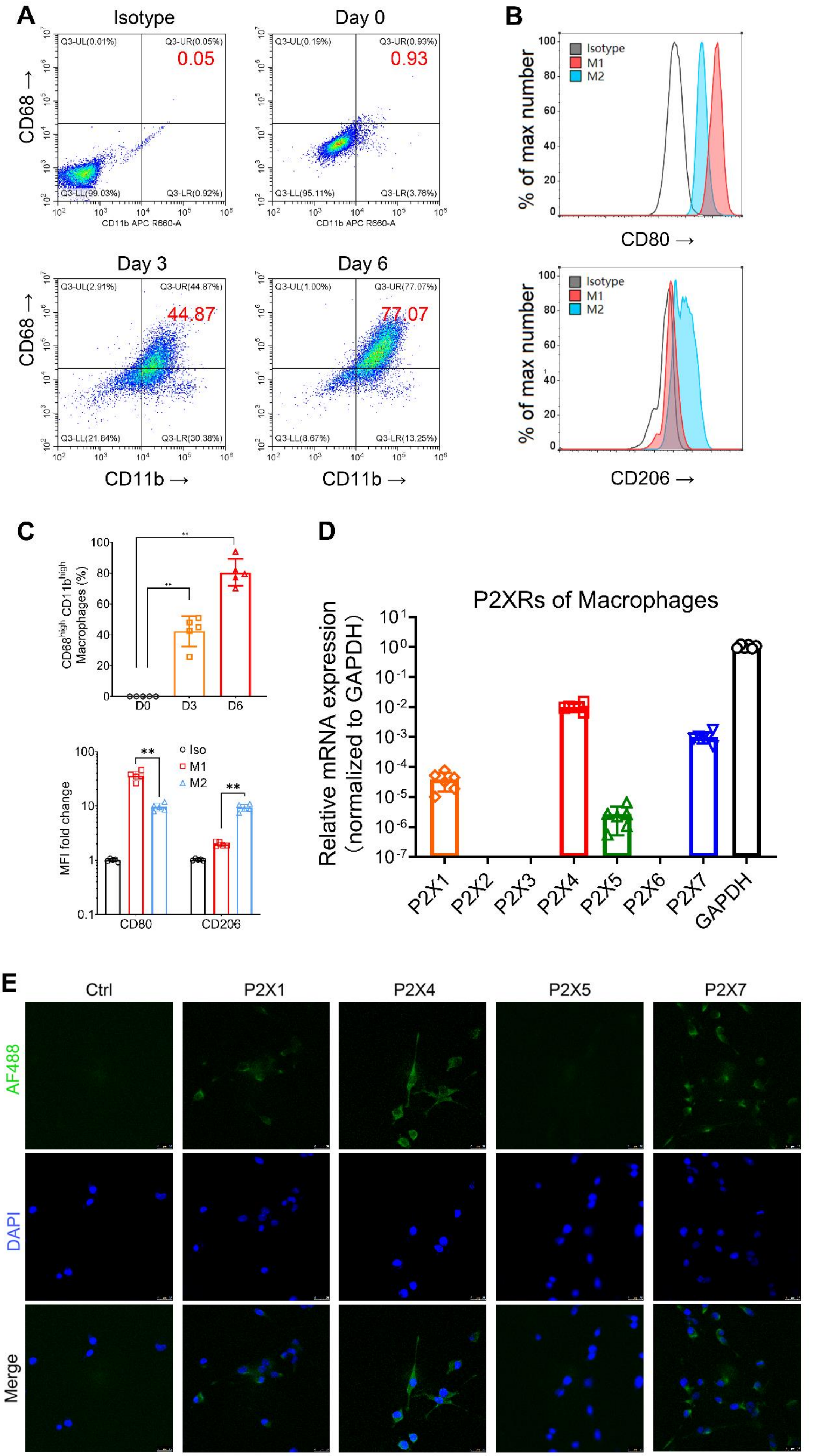
A
Isotype
Day 0
Day 3
Day 6
CD68 →
CD11b →
CD11b APC R660-A
Q3-UL(0.01%)
Q3-UR(0.05%)
0.05
Q3-LL(99.03%)
Q3-LR(0.92%)
Q3-UL(0.19%)
Q3-UR(0.93%)
0.93
Q3-LL(95.11%)
Q3-LR(3.76%)
Q3-UL(2.91%)
Q3-UR(44.87%)
44.87
Q3-LL(21.84%)
Q3-LR(30.38%)
Q3-UL(1.00%)
Q3-UR(77.07%)
77.07
Q3-LL(8.67%)
Q3-LR(13.25%)
B
% of max number
Isotype
M1
M2
CD80 →
CD206 →
C
CD68high CD11bhigh Macrophages (%)
D0
D3
D6
MFI fold change
Iso
M1
M2
CD80
CD206
D
P2XRs of Macrophages
Relative mRNA expression (normalized to GAPDH)
P2X1
P2X2
P2X3
P2X4
P2X5
P2X6
P2X7
GAPDH
E
Ctrl
P2X1
P2X4
P2X5
P2X7
AF488
DAPI
Merge

Figure S1. Introduction and polarization of PBMC-derived macrophages, related to Figures 1. (A) CD14-positive sorted PBMC were induced to differentiate into macrophages using 20 ng/mL M-CSF for 6 days. The expression of macrophage markers (CD68 and CD11b) was observed by flow cytometry. (B) M1/M2 polarization was induced in PBMC-derived macrophages (M1: 10 ng/mL IFN-γ and 100 ng/mL LPS; M2: 20 ng/mL IL-4 for 48h). Differences in the expression of M1/M2 surface markers were compared by flow cytometry. (C) Statistical analysis of A and B. (D) PBMC-derived macrophages were induced with 20ng/mL M-CSF for 6 days. The relative mRNA expression levels of each P2XR subtype (P2X1-7) were detected by qPCR and expressed as $2^{\Delta Ct}$ relative to GAPDH. (E) P2X receptors were labeled with Alexa Fluor 488 (green), and nuclei were counterstained with DAPI. Immunofluorescence images were captured using confocal microscopy. Scale bar: 25μm. Representative image shown (N = 3 independent experiments). All data are presented as mean ± SD. Data points represent independent biological replicates.

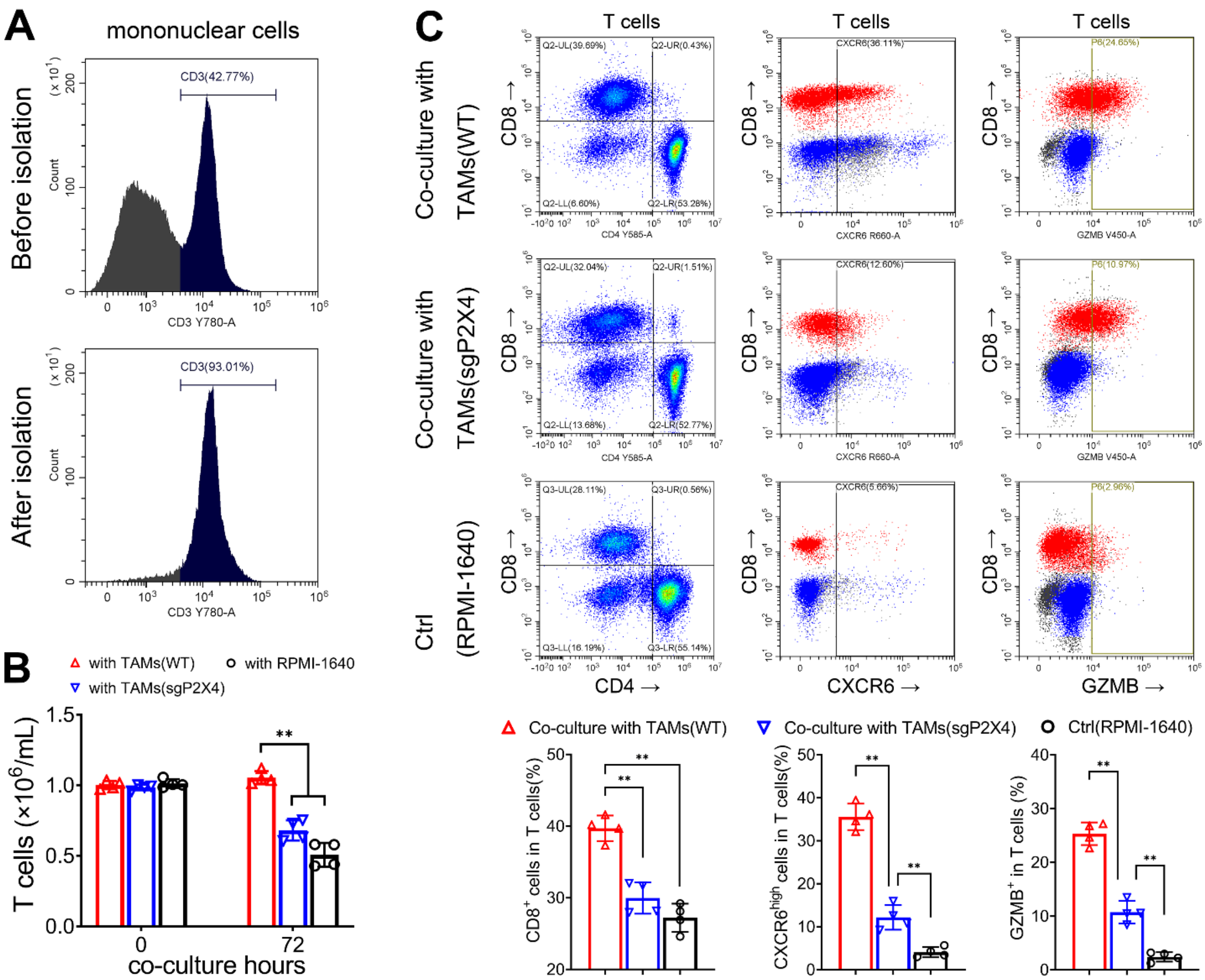

Figure S2. Validation of P2X4-Mediated TAM Regulation of T Cell Function in Purified Splenic T Cells, related to Figures 4. (A) Mouse splenocytes were isolated, and $CD3^+$ T cells were subsequently purified by magnetic negative selection. The $CD3^+$ purity of the isolated cells was verified by flow cytometry (N = 3 independent experiments). (B) Splenic T cells were co-cultured with either TAMs (sgP2X4) or TAMs (WT) at a 4:1 ratio. Viable cells were quantified using a cell viability analyzer after 72 hours of co-culture (** $P < 0.01$, Student's t-test). (C) After co-culture, T cells were harvested and stained with CD3 (PE-Cy7), CD4 (PE), CD8 (FITC) and CXCR6 (APC) for flow cytometry. Another set of cells were fixed for GzmB (BV421) staining. Data were compared by histogram (* $P < 0.05$, ** $P < 0.01$, Student's t-test). All data are presented as mean ± SD. Data points represent independent biological replicates.

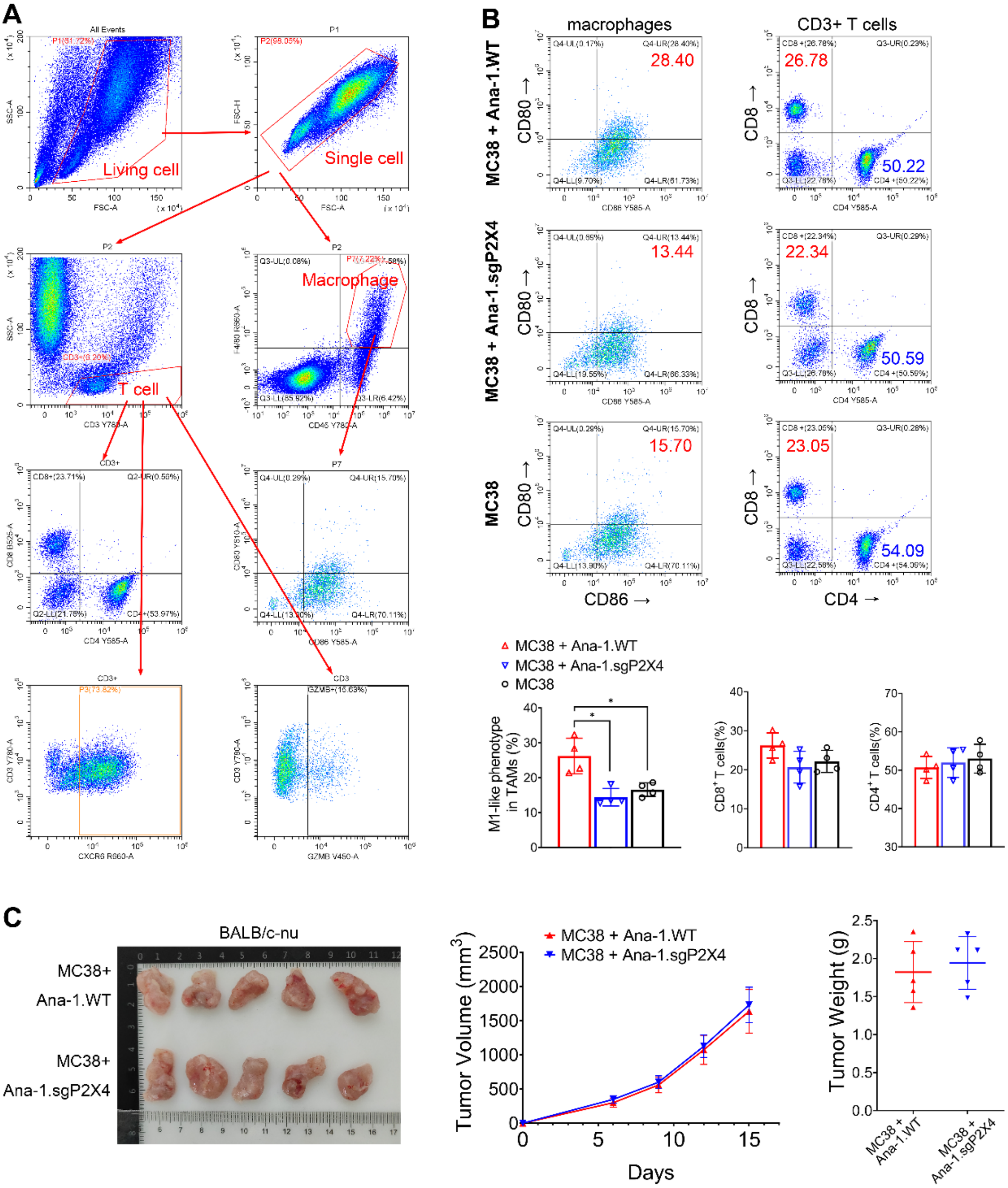

Figure S3. Macrophage and T cell infiltration in MC38 xenografts in mice, related to Figures 5. tumor-infiltrating cells in subcutaneous tumors from each group were isolated and stained refer to Figure5B. (A) Gating strategy for macrophage and T cell in tumor-infiltrating cells. (B) The proportion of subpopulations among macrophages and T cells in each group was observed by flow cytometry. (* P < 0.05, Student's t-test). (C) 6-week BALB/c-nu female mice were divided into 2 groups and inoculated with a mixture of: MC38 cells + Ana-1.WT cells, or MC38 cells + Ana-1.sgP2X4 cells. Tumor volume and weight were compared (Student's t-test). All data are presented as mean ± SD. Data points represent independent biological replicates.

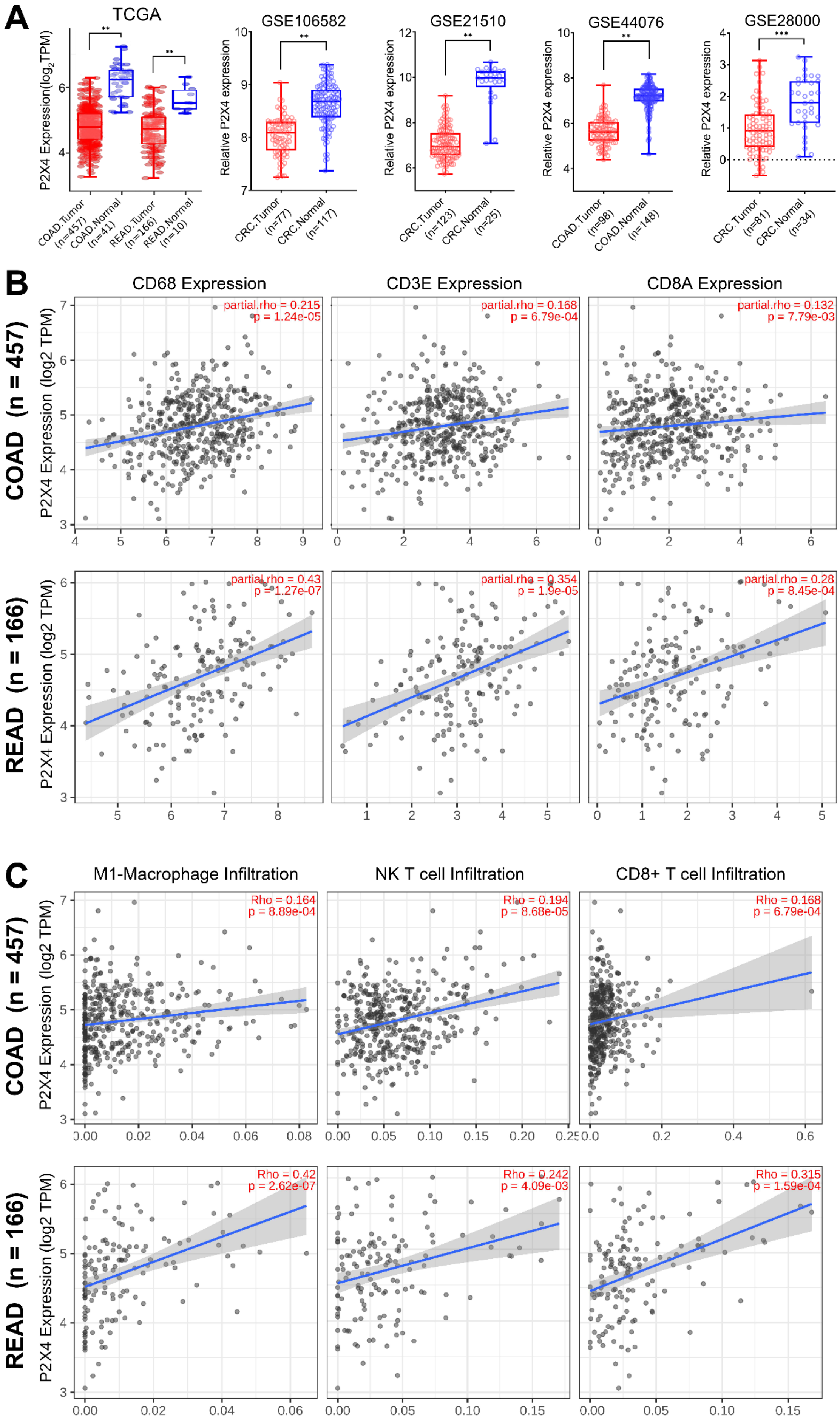

A
TCGA
GSE106582
GSE21510
GSE44076
GSE28000
P2X4 Expression(log2TPM)
Relative P2X4 expression
COAD.Tumor (n=457)
COAD.Normal (n=41)
READ.Tumor (n=166)
READ.Normal (n=10)
CRC.Tumor (n=77)
CRC.Normal (n=117)
CRC.Tumor (n=123)
CRC.Normal (n=25)
COAD.Tumor (n=98)
COAD.Normal (n=148)
CRC.Tumor (n=81)
CRC.Normal (n=34)
B
CD68 Expression
CD3E Expression
CD8A Expression
COAD (n = 457)
READ (n = 166)
P2X4 Expression (log2 TPM)
partial.rho = 0.215
p = 1.24e-05
partial.rho = 0.168
p = 6.79e-04
partial.rho = 0.132
p = 7.79e-03
partial.rho = 0.43
p = 1.27e-07
partial.rho = 0.354
p = 1.9e-05
partial.rho = 0.28
p = 8.45e-04
C
M1-Macrophage Infiltration
NK T cell Infiltration
CD8+ T cell Infiltration
Rho = 0.164
p = 8.89e-04
Rho = 0.194
p = 8.68e-05
Rho = 0.168
p = 6.79e-04
Rho = 0.42
p = 2.62e-07
Rho = 0.242
p = 4.09e-03
Rho = 0.315
p = 1.59e-04

Figure S4. Correlation of P2X4 expression with immune infiltration in CRC, related to Figures 6. (A) Differences in P2X4 gene expression between tumor tissue and adjacent normal tissue were analyzed in CRC samples from the TCGA and the GEO database (** $P < 0.01$, Wilcoxon test). (B) Correlation of P2X4 with macrophage/T cell surface markers in CRC patients from TCGA database (Spearman Analysis). (C) Correlation of P2X4 with macrophage/T cell infiltration levels in CRC patients from TCGA database (Spearman Analysis). All data are presented as mean ± SD. Each dot represents one individual patient sample.

Supplementary Table S1: Primer sequences used in this study

species	Primers	sequences（5' to 3'）
human	GAPDH-f	ATCCCATCACCATCTTCCAGG
	GAPDH-r	CCTTCTCCATGGTGGTGAAGAC
	P2X1-f	GGCTGACTACGTCTTCCCAG
	P2X1-r	GCGCAGTAGCCTTGAGTCT
	P2X2-f	AGCTGGGCTTTATCGTGGAGA
	P2X2-r	TTGGGGTTGCACTCCGATG
	P2X3-f	TTCTTGCACGAGAAGGCTTAC
	P2X3-r	CCATGACTCTGTTGGCGTAGA
	P2X4-f	TGGCGGATTATGTGATACCAGC
	P2X4-r	GTCGCATCTGGAATCTCGGG
	P2X5-f	ACCCTCACTATTCTTTTAGCCGT
	P2X5-r	CAAATCTGAAGTTGTACCCGGAG
	P2X6-f	GTCCGTCCCACTGGCTAAC
	P2X6-r	CTGGCCTGTTTTTACACCGTG
	P2X7-f	GTGCCGAAAACTTCACTGTGC
	P2X7-r	CTGGCAGGATGTTTCTCGTGG
	CD80-f	GGCCCGAGTACAAGAACCG
	CD80-r	TCGTATGTGCCCTCGTCAGAT
	CD86-f	CTGCTCATCTATACACGGTTACC
	CD86-r	GGAAACGTCGTACAGTTCTGTG
	CD206-f	CTACAAGGGATCGGGTTTATGGA
	CD206-r	TTGGCATTGCCTAGTAGCGTA
	CD163-f	TTTGTCAACTTGAGTCCCTTCAC
	CD163-r	TCCCGCTACACTTGTTTTCAC
	IL1B-f	ATGATGGCTTATTACAGTGGCAA
	IL1B-r	GTCGGAGATTCGTAGCTGGA
	CXCL10-f	GTGGCATTCAAGGAGTACCTC
	CXCL10-r	TGATGGCCTTCGATTCTGGATT

	IL6-f	CCTGAACCTTCCAAAGATGGC
	IL6-r	TTCACCAGGCAAGTCTCCTCA
	TNF-f	CCTCTCTCTAATCAGCCCTCTG
	TNF-r	GAGGACCTGGGAGTAGATGAG
	IFNB1-f	GTCACTGTGCCTGGACCATAG
	IFNB1-r	GTTTCGGAGGTAACCTGTAAGTC
	STAT1-f	CAGCTTGACTCAAAATTCCTGGA
	STAT1-r	TGAAGATTACGCTTGCTTTTCCT
	IRF1-f	ATGCCCATCACTCGGATGC
	IRF1-r	CCCTGCTTTGTATCGGCCTG
	IFIT2-f	GACACGGTTAAAGTGTGGAGG
	IFIT2-r	TCCAGACGGTAGCTTGCTATT
	IL10-f	GTTGTTAAAGGAGTCCTTGCTG
	IL10-r	TTCACAGGGAAGAAATCGATGA
	TGFB1-f	CTAATGGTGGAAACCCACAACG
	TGFB1-r	TATCGCCAGGAATTGTTGCTG
	tRLeu-f	CACCCAAGAACAGGGTTTGT
	tRLeu-r	TGGCCATGGGTATGTTGTTA
	B2M-f	TGCTGTCTCCATGTTTGATGTATCT
	B2M-r	TCTCTGCTCCCCACCTCTAAGT
Mouse	Ifnb1-f	CAGCTCCAAGAAAGGACGAAC
	Ifnb1-r	GGCAGTGTAACTCTTCTGCAT
	Il1b-f	GCAACTGTTCCTGAACTCAACT
	Il1b-r	ATCTTTTGGGGTCCGTCAACT
	Cxcl10-f	CCAAGTGCTGCCGTCATTTTC
	Cxcl10-r	GGCTCGCAGGGATGATTTCAA
	Il6-f	TAGTCCTTCCTACCCCAATTTCC
	Il6-r	TTGGTCCTTAGCCACTCCTTC
	Tnf-f	CCTGTAGCCCACGTCGTAG

Tnf-r	GGGAGTAGACAAGGTACAACCC
Il10-f	AGCCTTATCGGAAATGATCCAGT
Il10-r	GGCCTTGTAGACACCTTGGT
Arg1-f	ACAAGACAGGGCTCCTTTCAG
Arg1-r	GGCTTATGGTTACCCTCCCG
Tgfb1-f	CCACCTGCAAGACCATCGAC
Tgfb1-r	CTGGCGAGCCTTAGTTTGGAC
