# Supplementary material for: M1-like macrophages regulate T cell infiltration in colorectal cancer through P2X4 receptor
Source: iScience. 2025 Sep 5;28(10):113517. doi: 10.1016/j.isci.2025.113517 (PMC12478113; doi:10.1016/j.isci.2025.113517)
Supplement: Data S1. Original Western blot images [file mmc2.pdf]

FIG1A
p-STAT1(91kD)

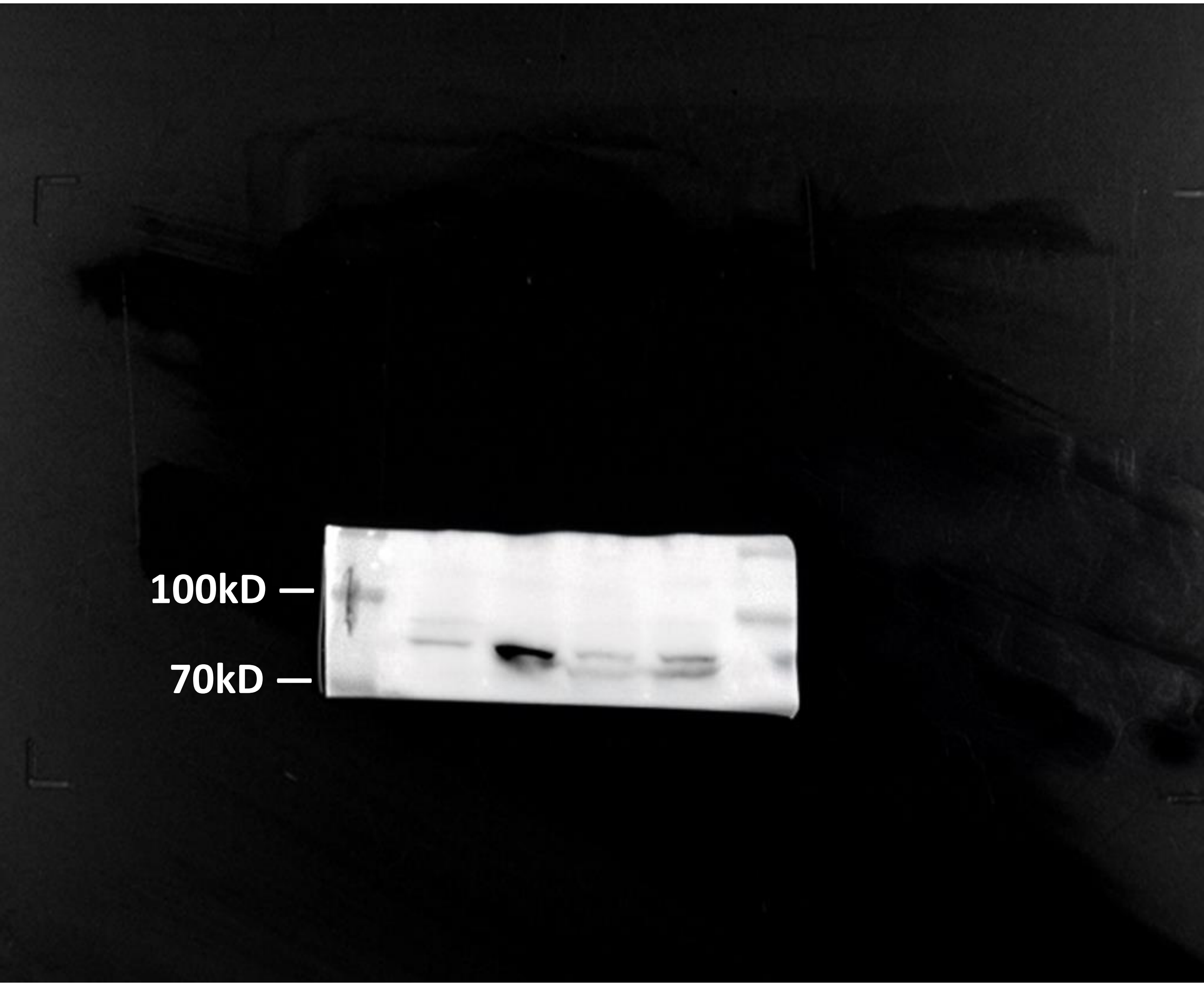

FIG1A
STAT1(91kD)

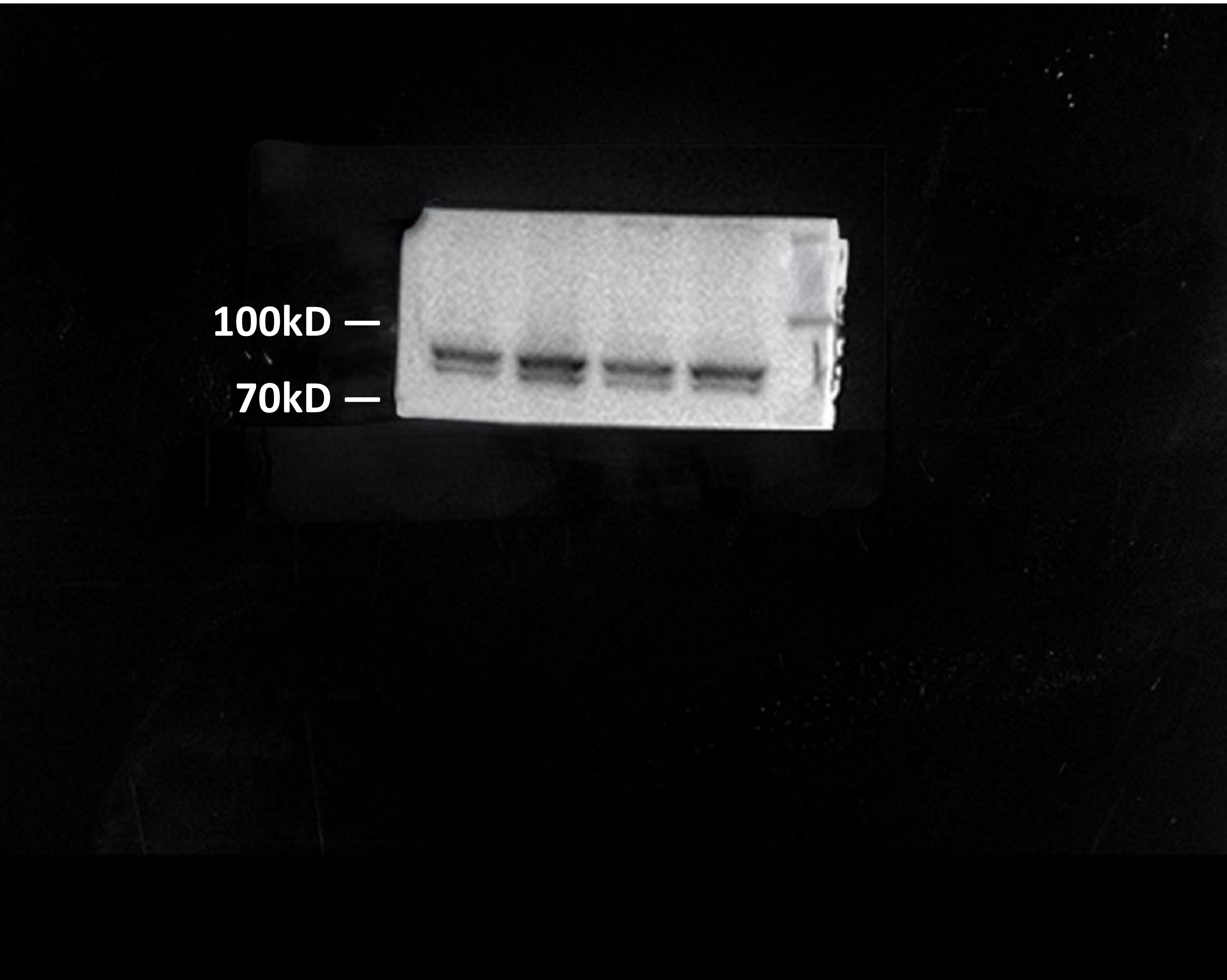

FIG1A
p-STAT3(88kD)

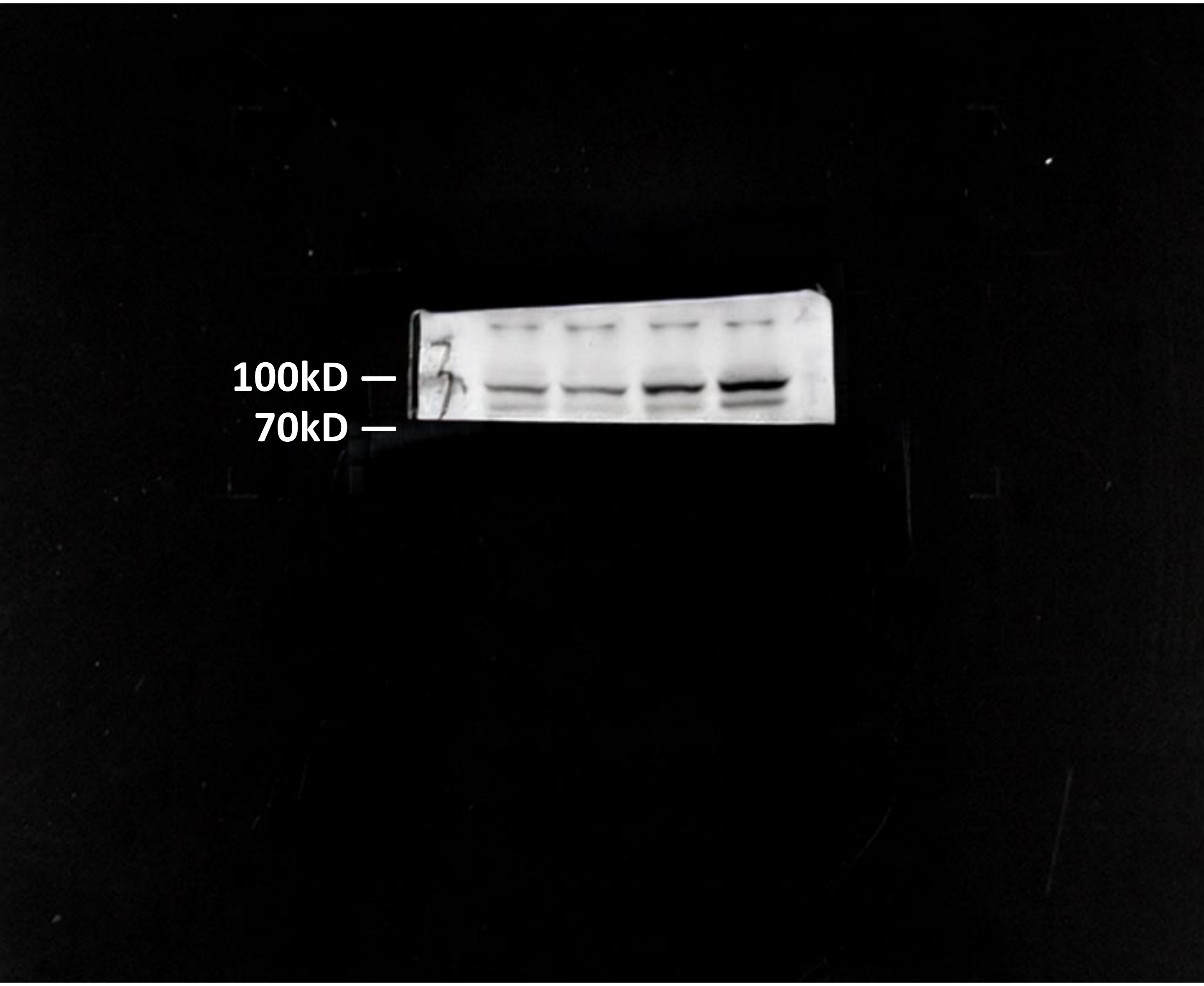

FIG1A
p-STAT6(100kD)

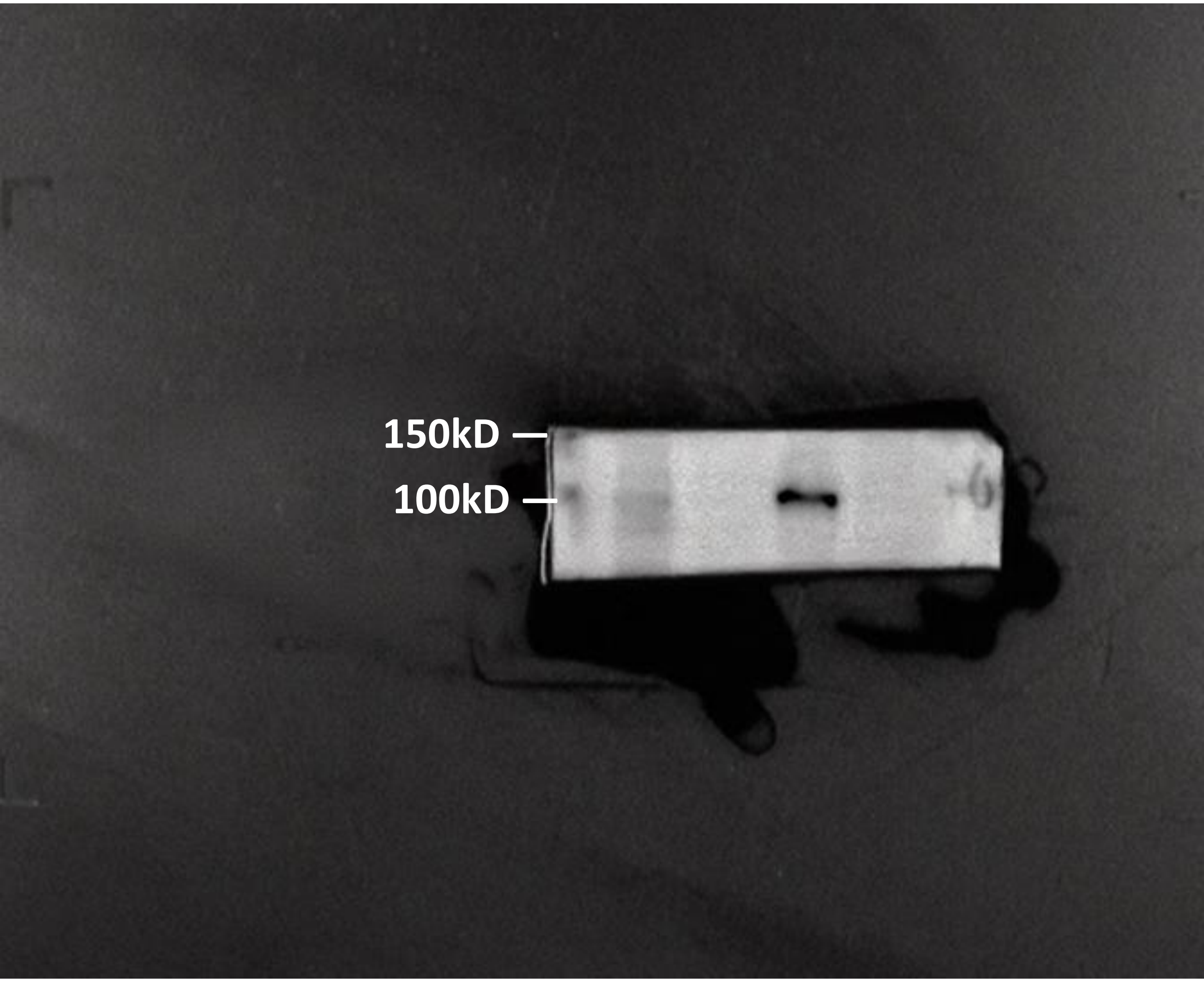

FIG1A

STAT3(88kD); STAT6(100kD)

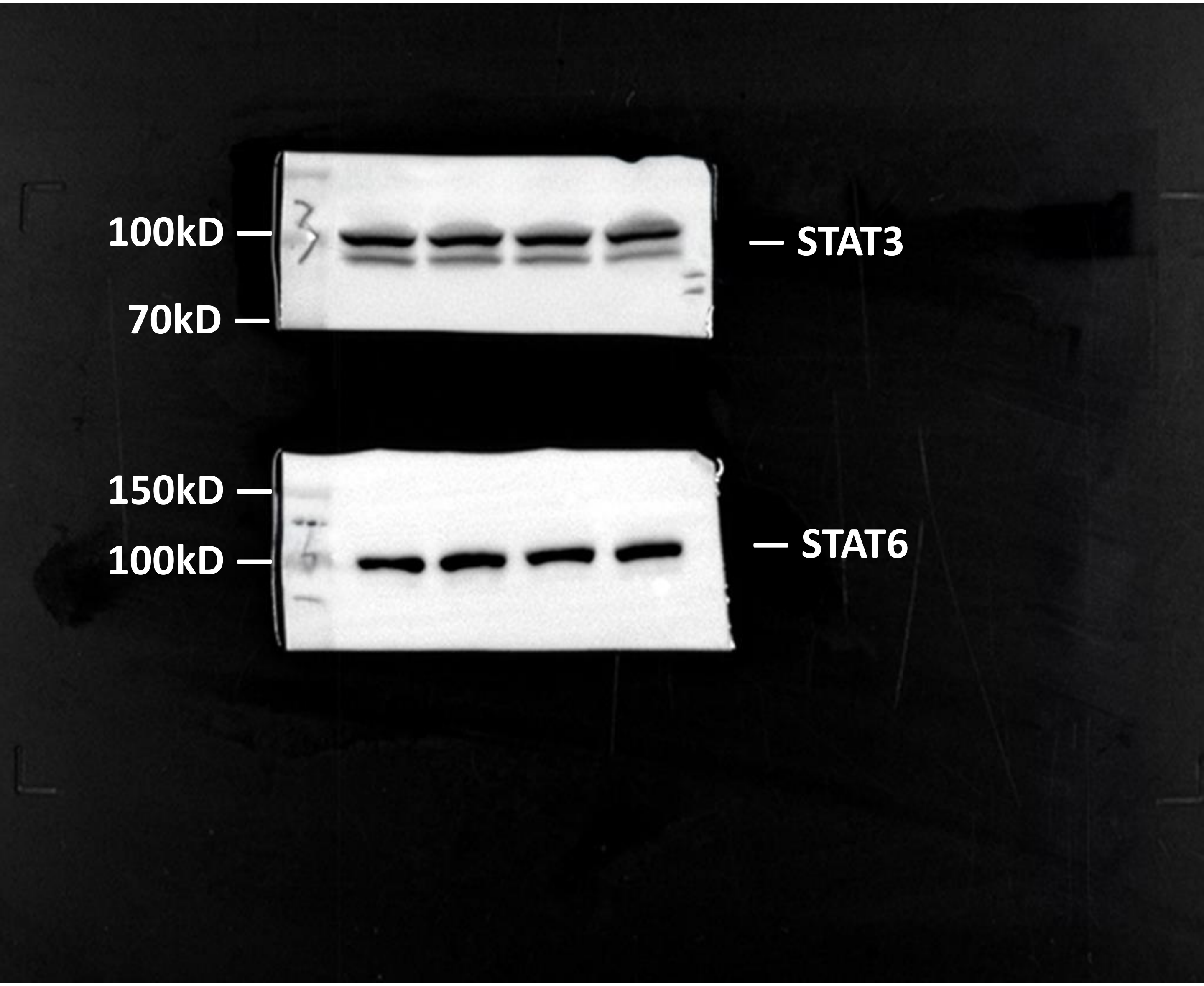

FIG1A

β-Actin(42kD)

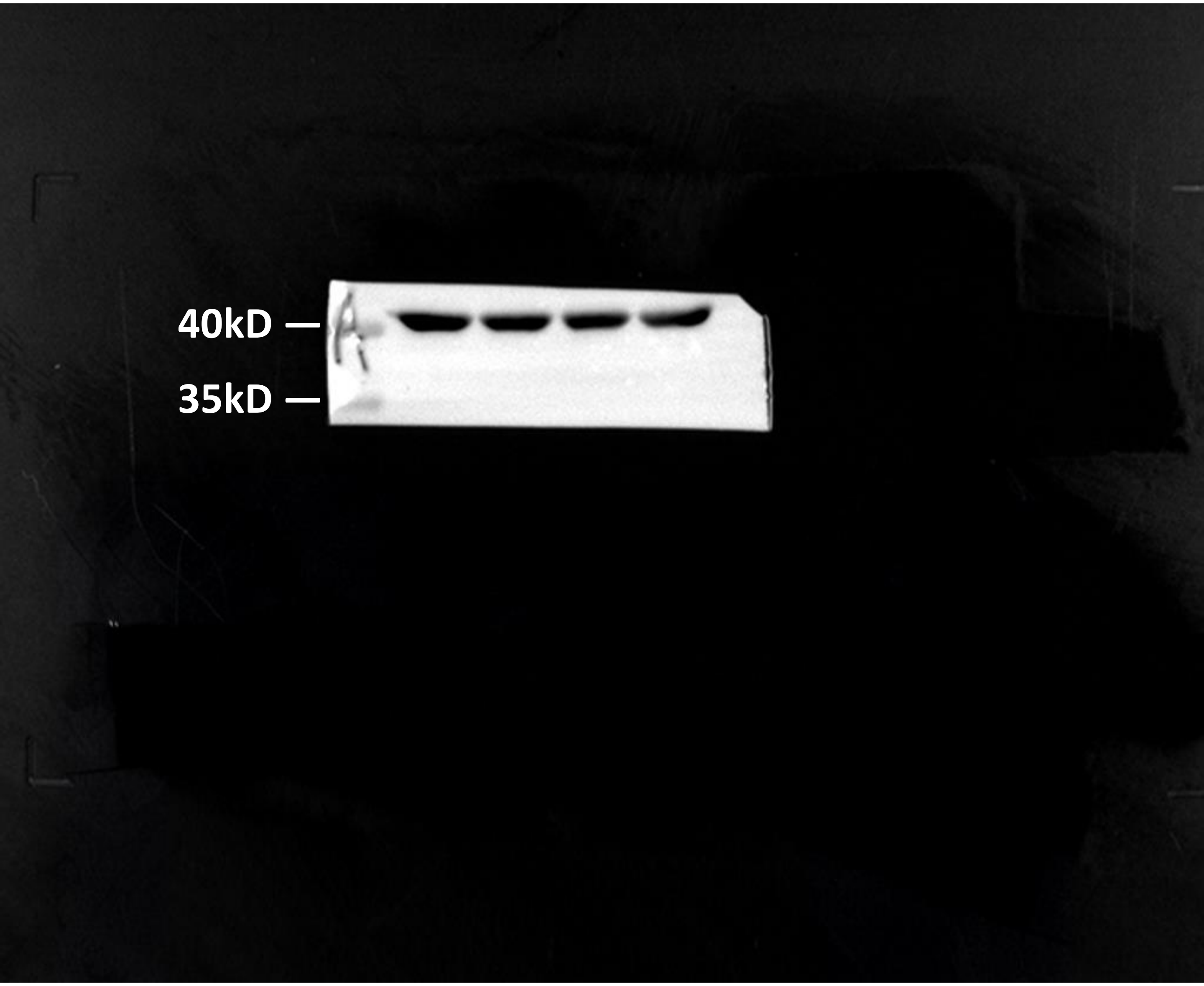

FIG1B

p-STAT1(91kD)

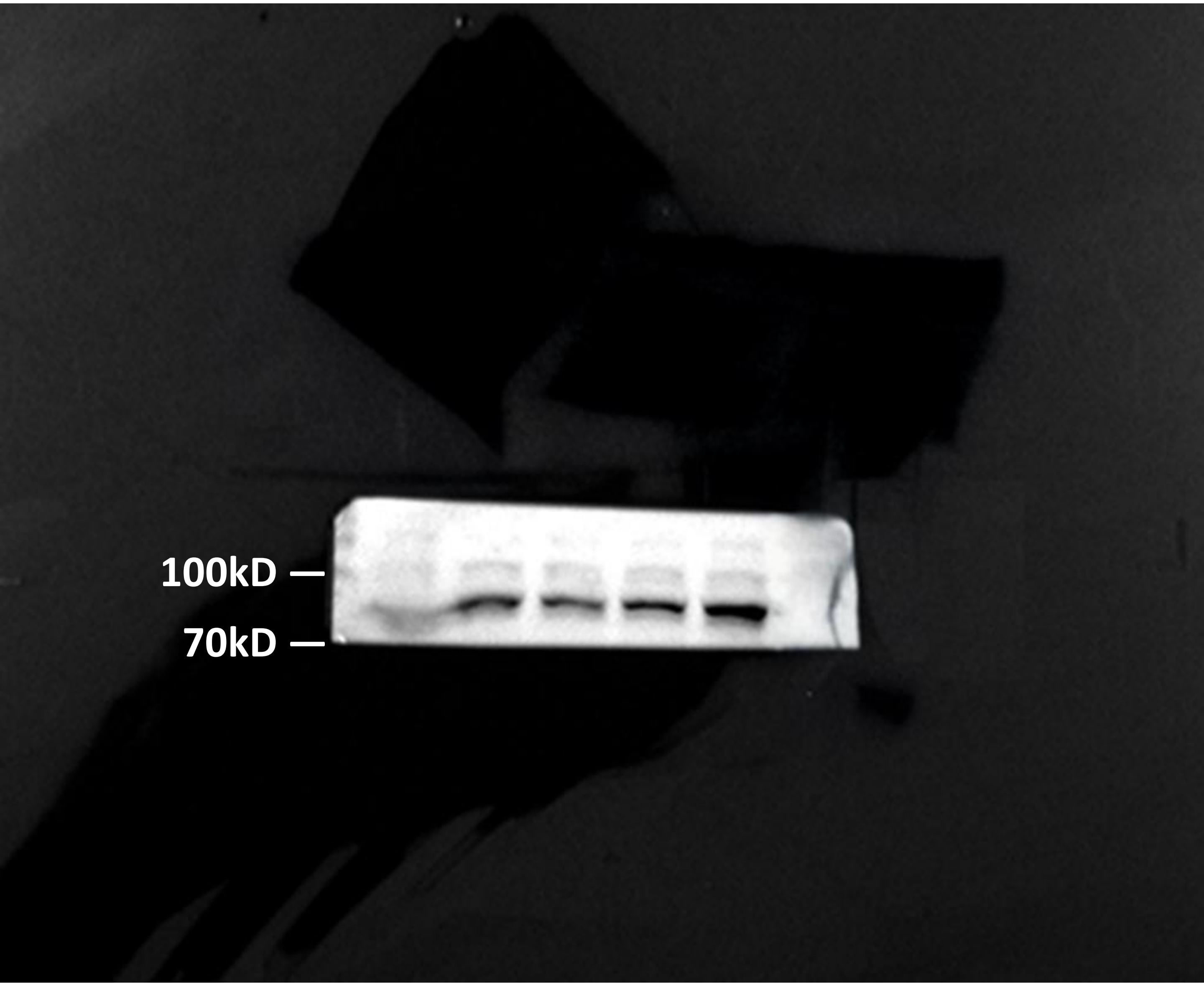

FIG1B

p-STAT3(88kD)

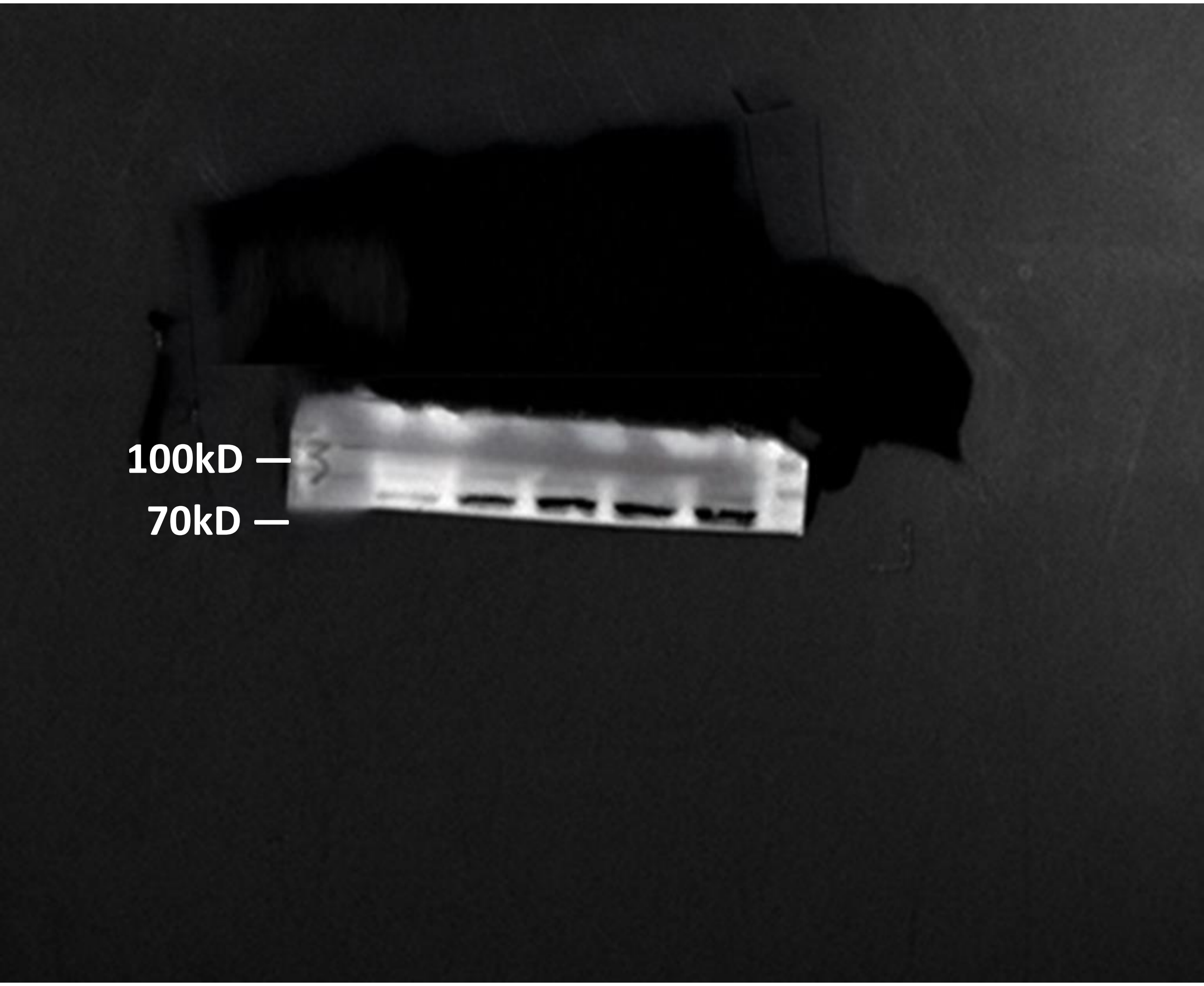

FIG1B

STAT1(91kD);   STAT3(88kD)

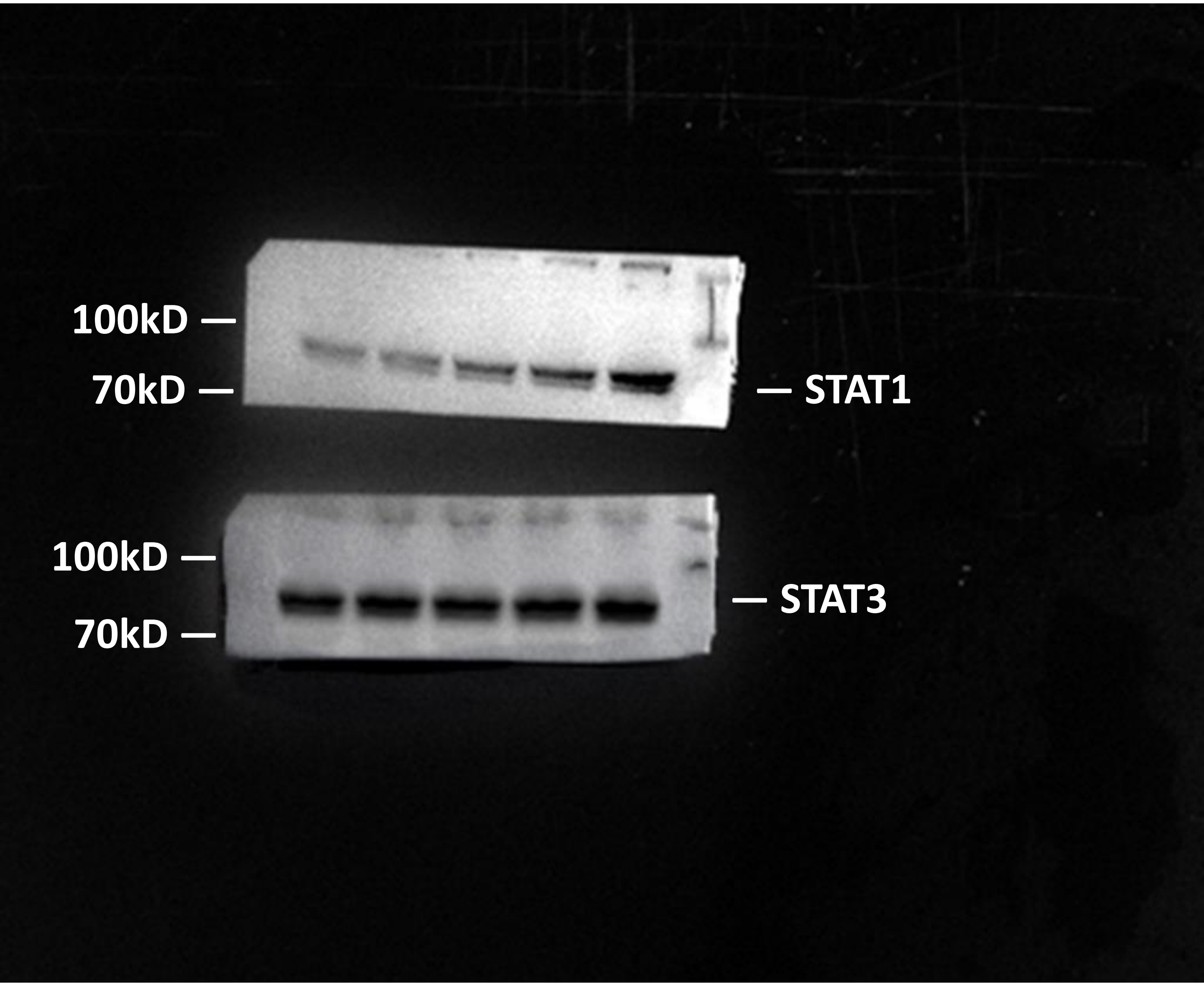

FIG1B
p-STAT2(113kD)

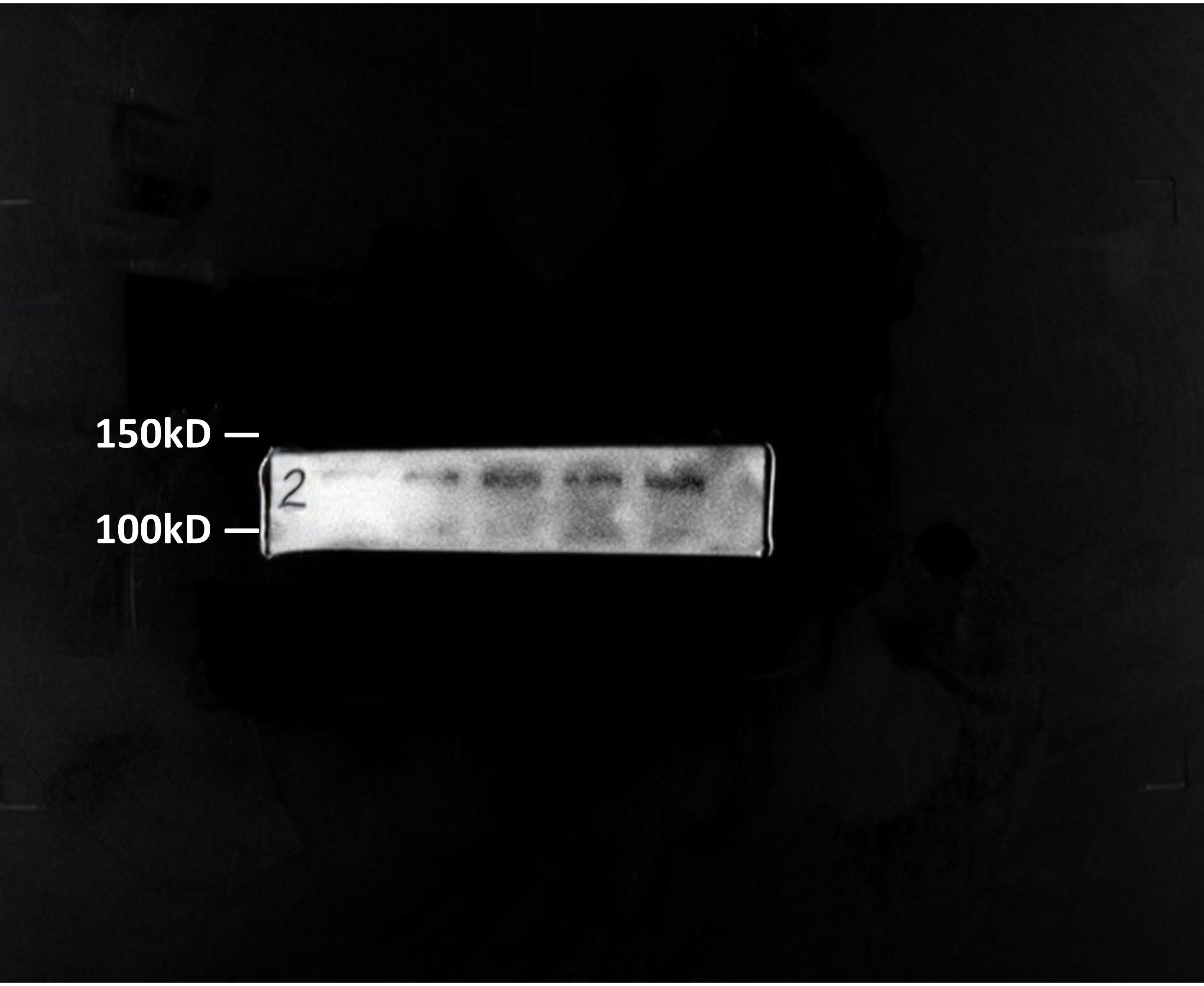

FIG1B

STAT2(113kD)

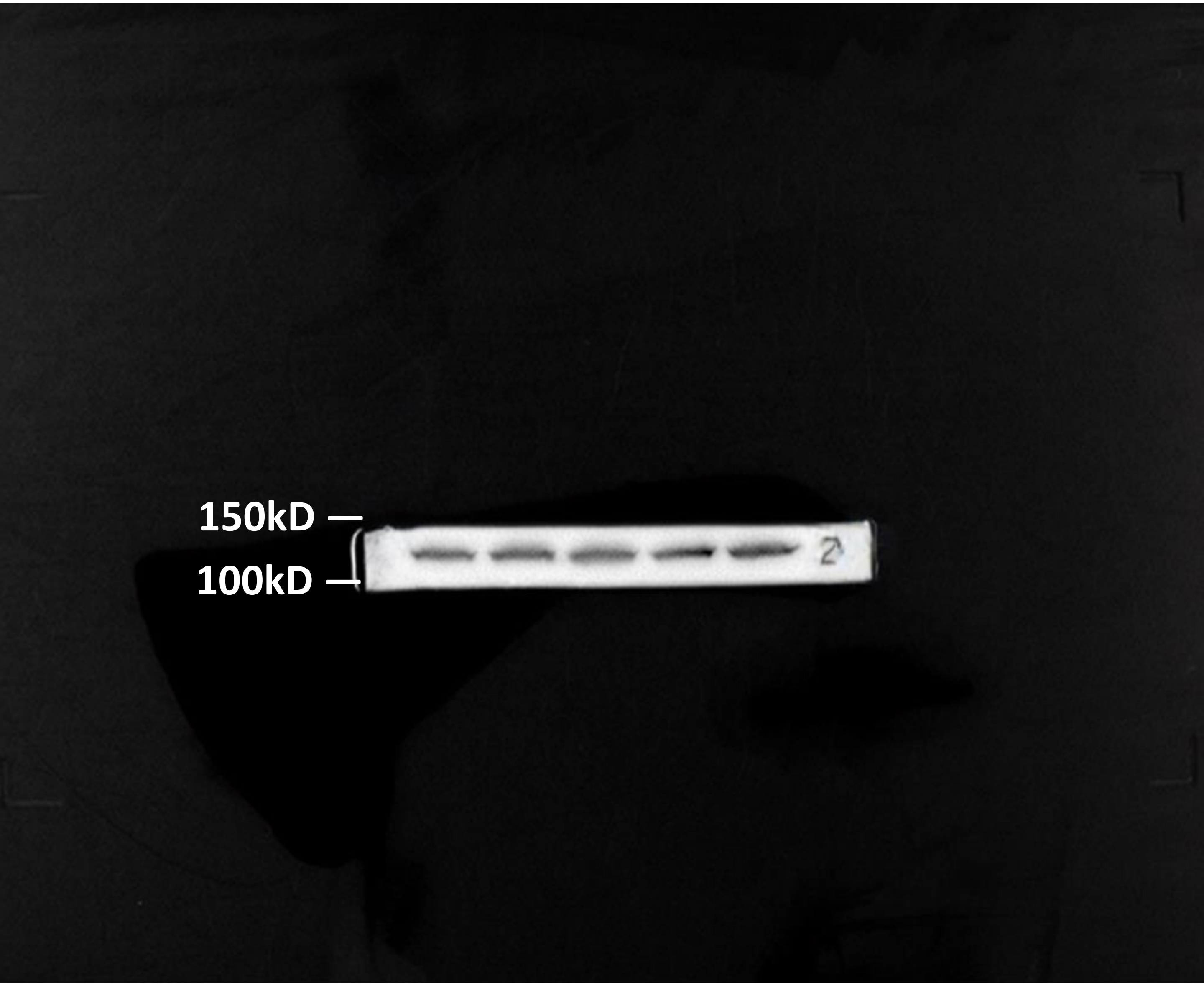

FIG1B

β-Actin(42kD)

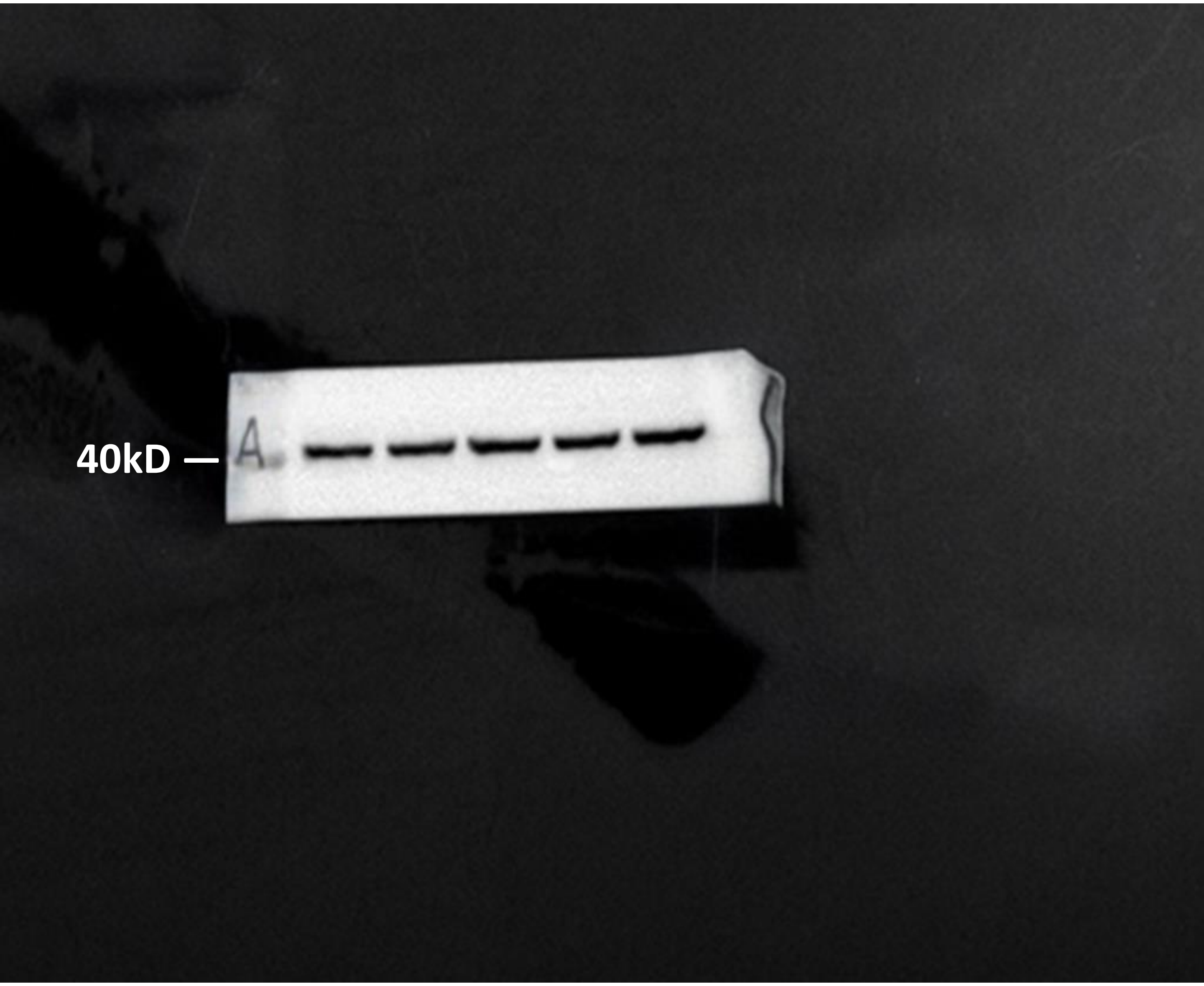

FIG1E

p-STAT3(88kD); p-STAT1(91kD)

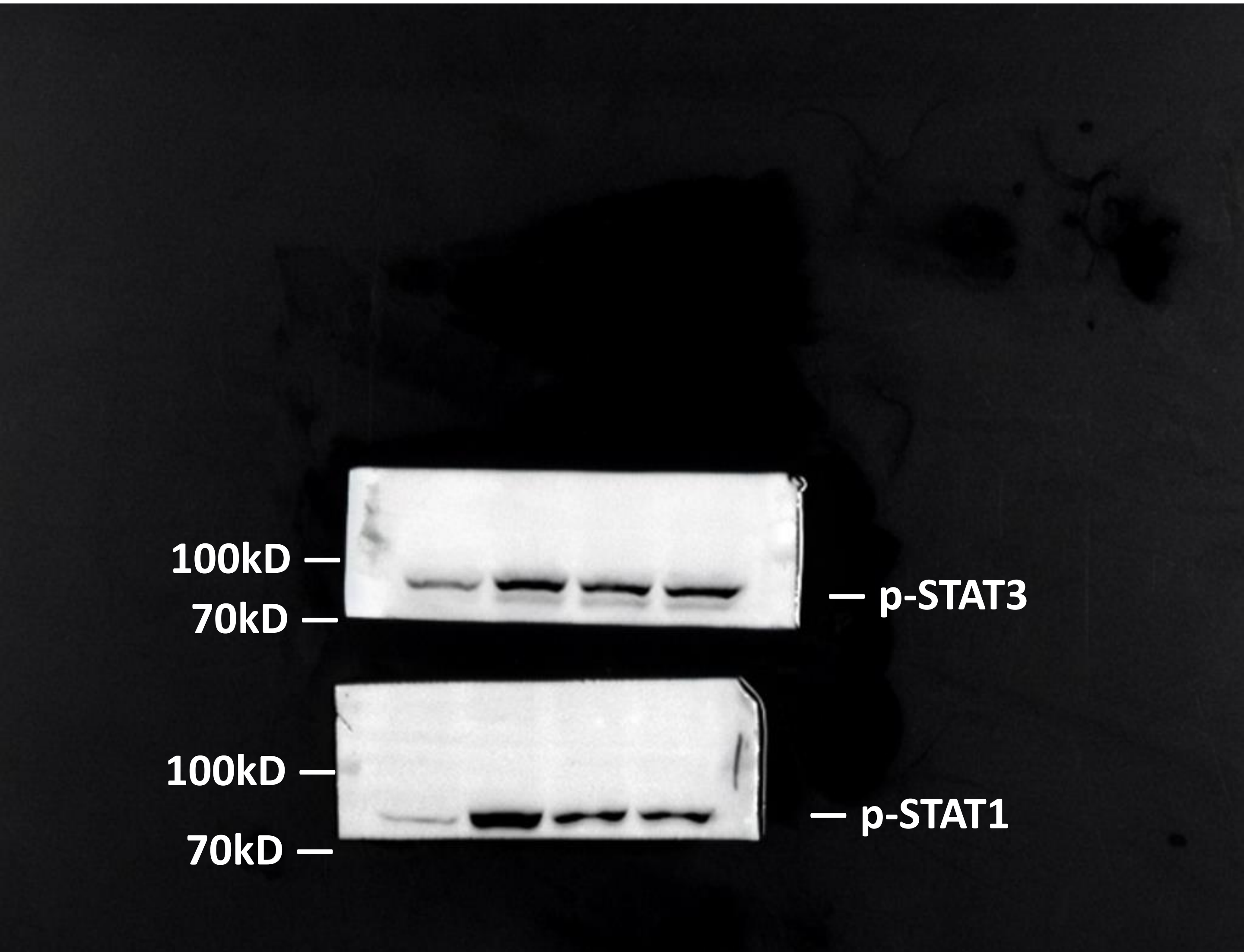

FIG1E
p-STAT2(113kD)

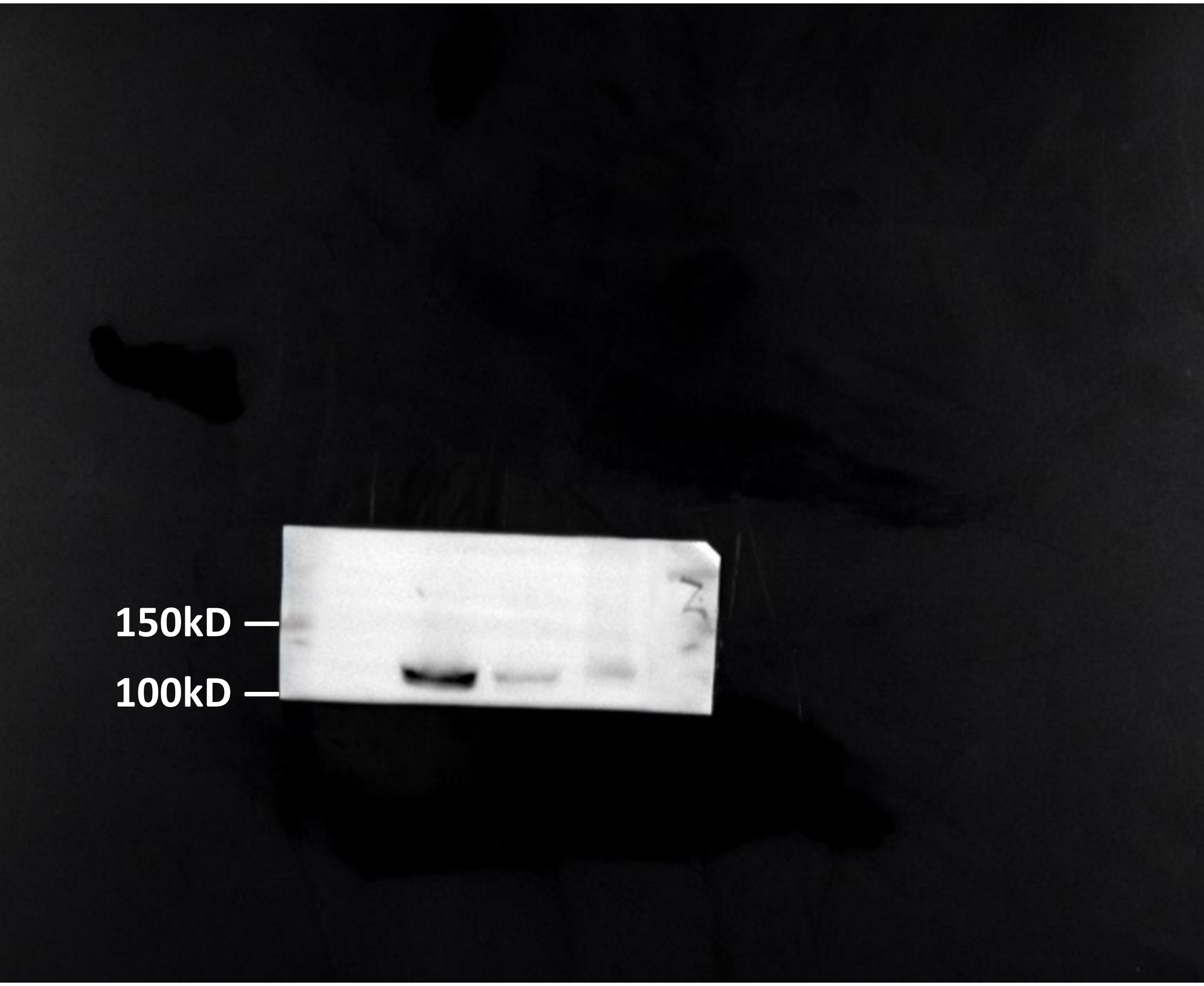

FIG1E

β-Actin(42kD)

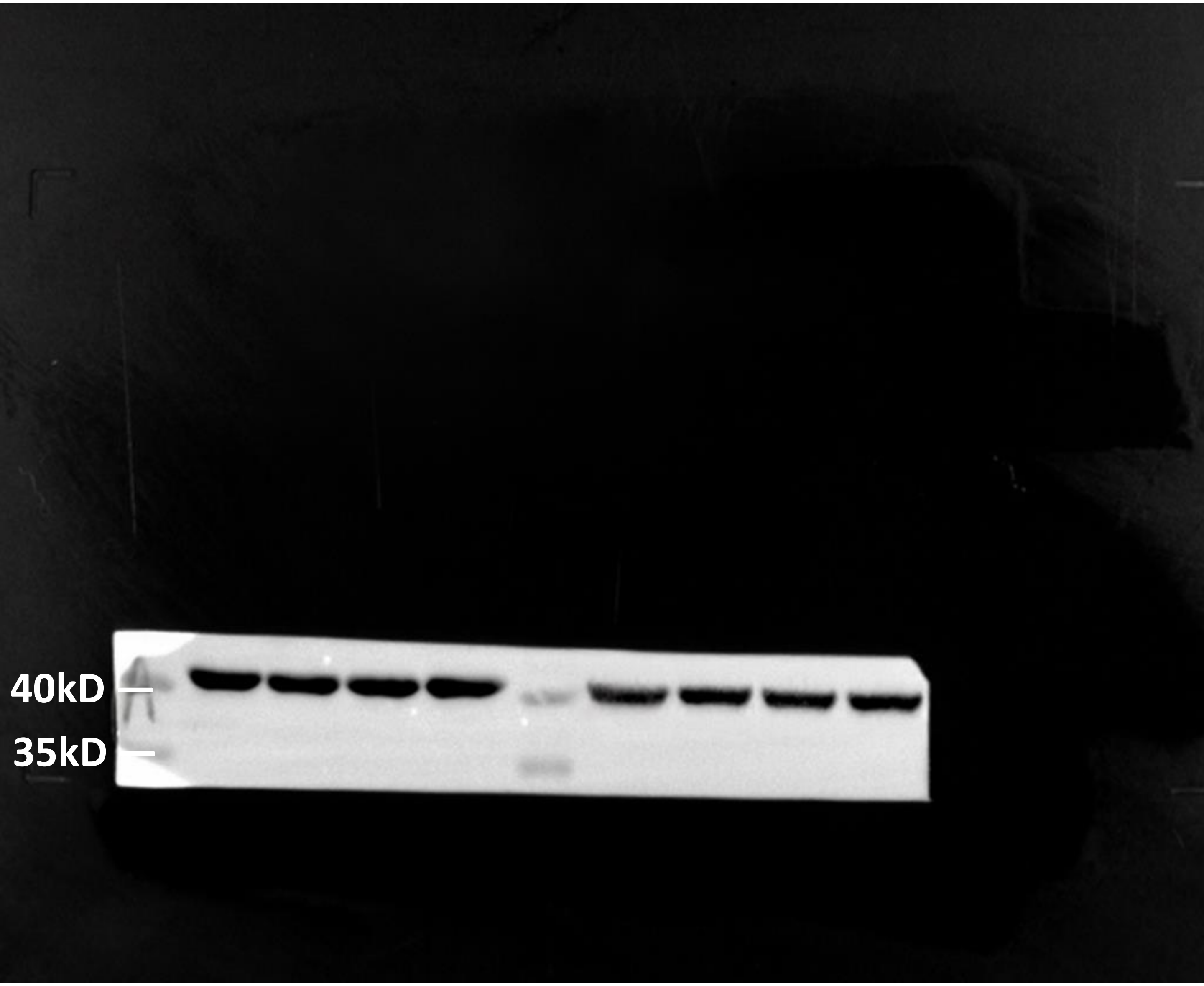

FIG1G

p-STING(41kD); STING(37kD)

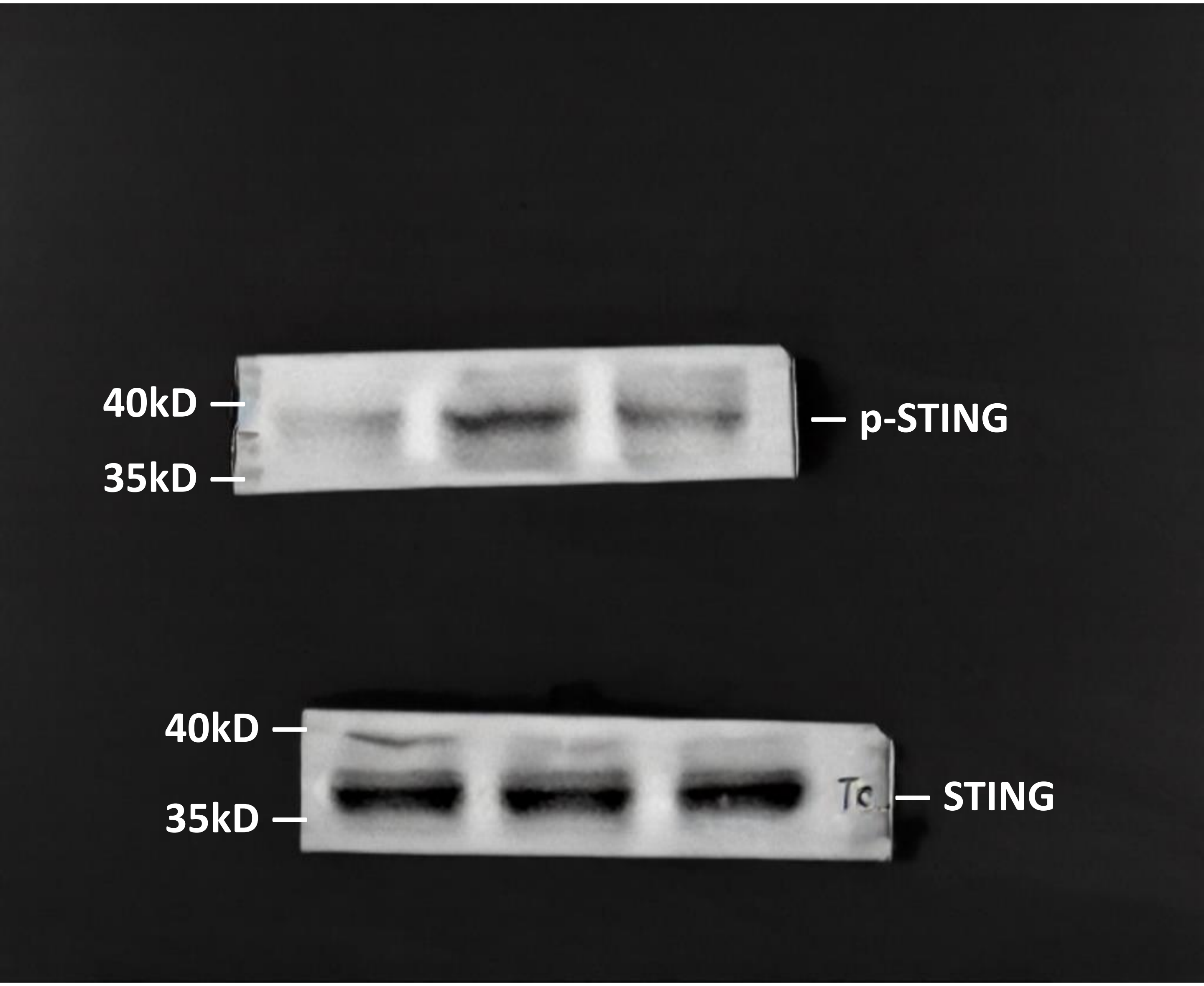

FIG1G

p-IRF3(55kD); IRF3(55kD)

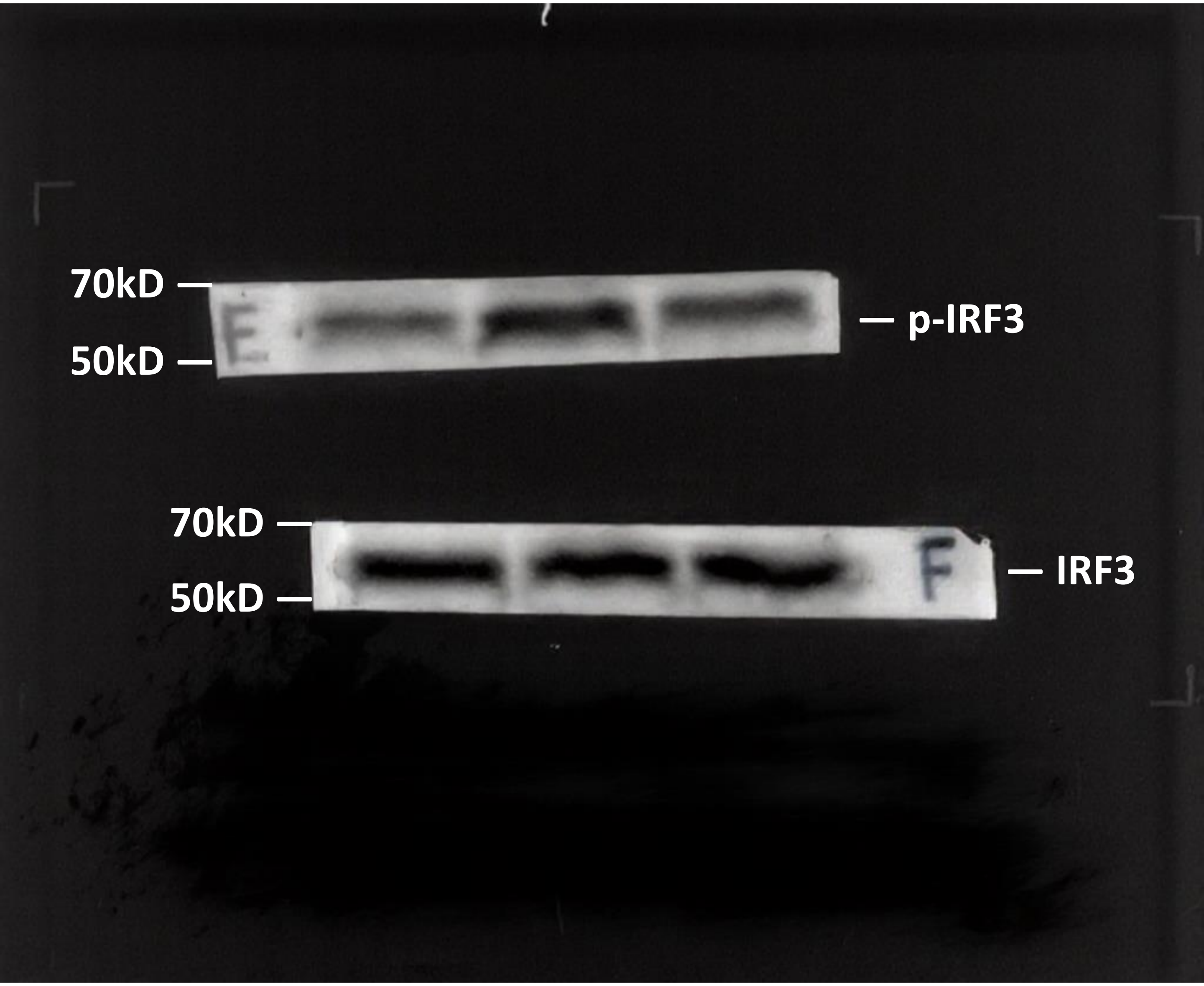

FIG1G

p-STAT1(91kD); STAT1(91kD)

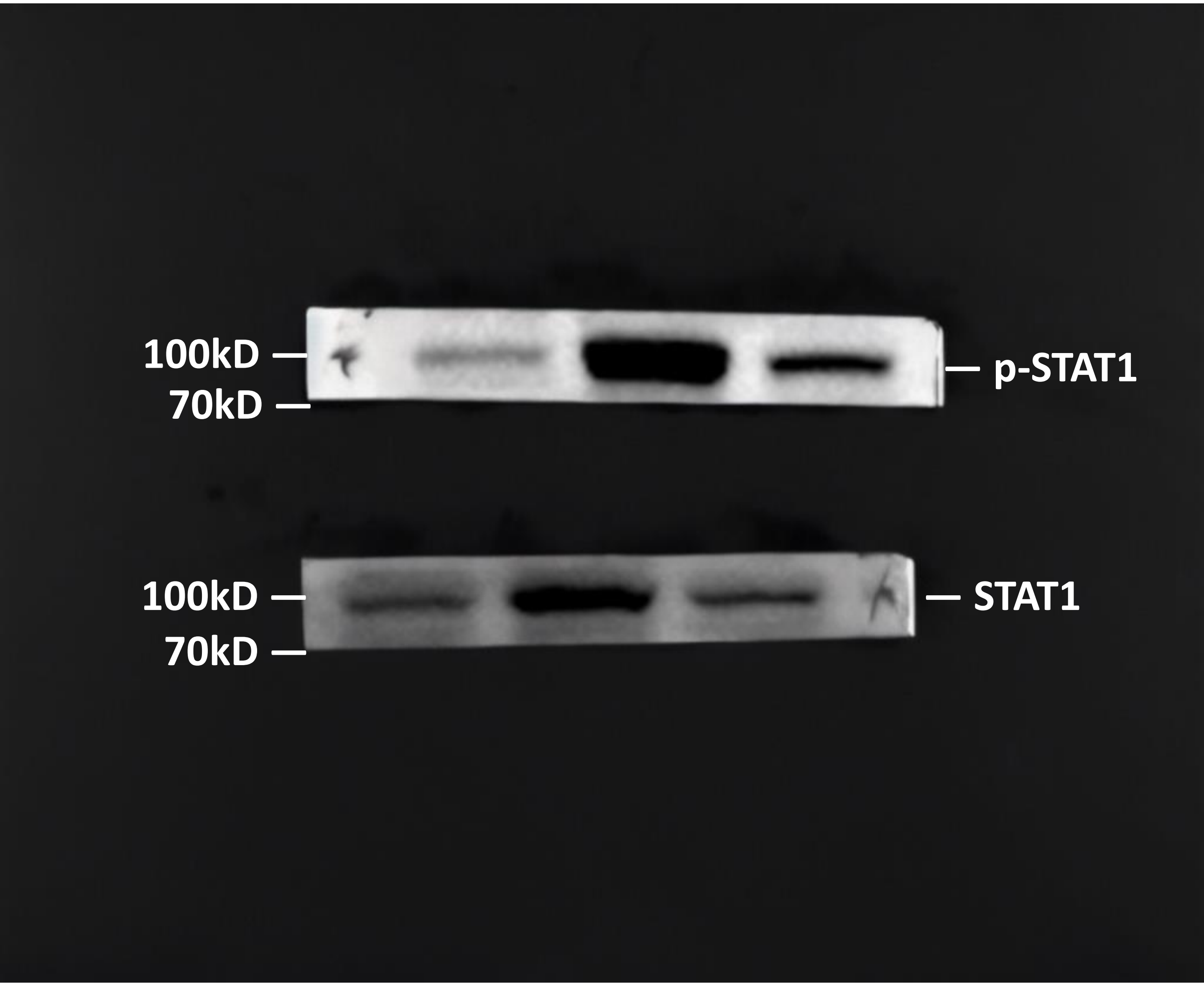

FIG1G

β-Actin(42kD)

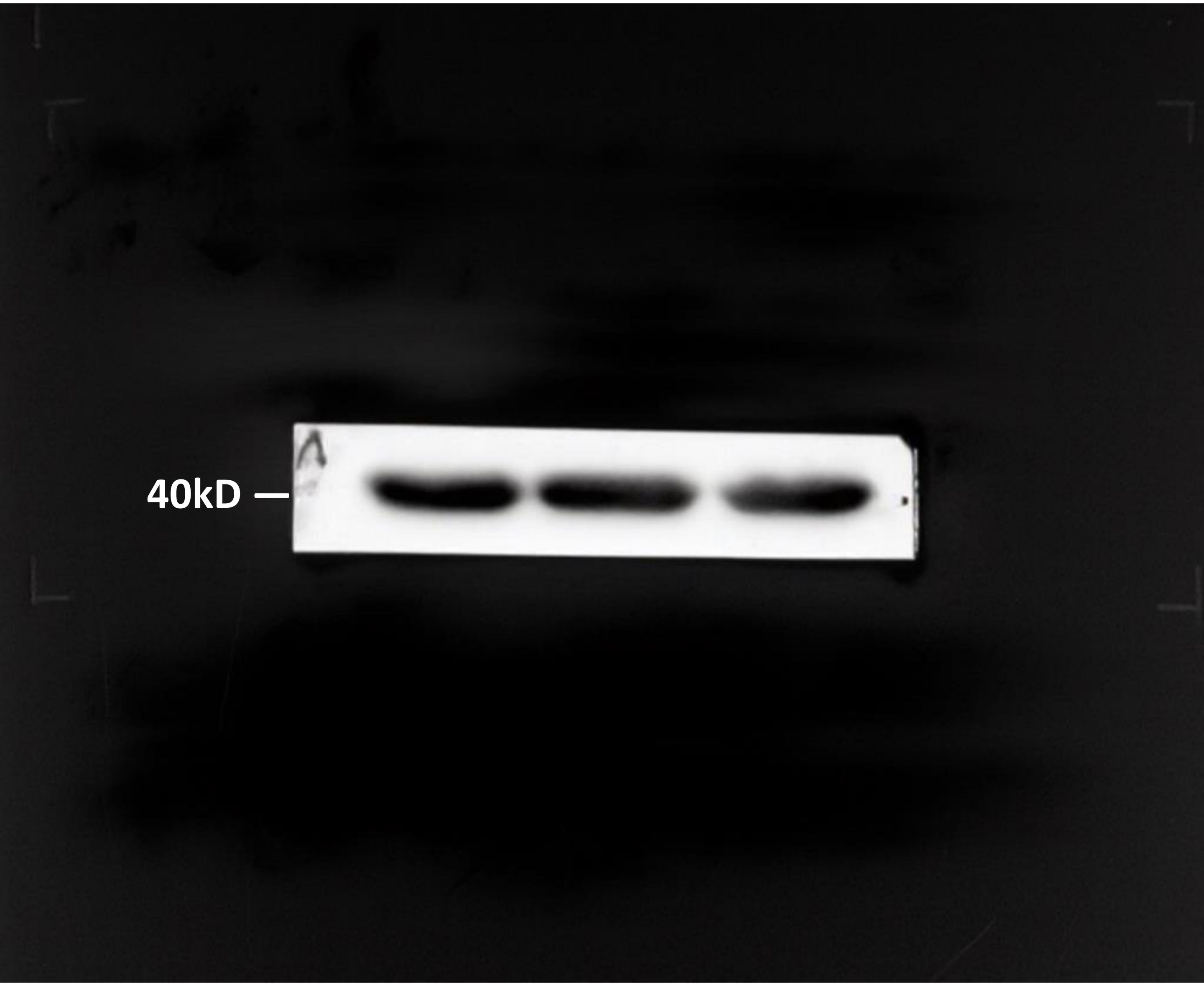

FIG2A

p-STAT1(91kD); STAT1(91kD)

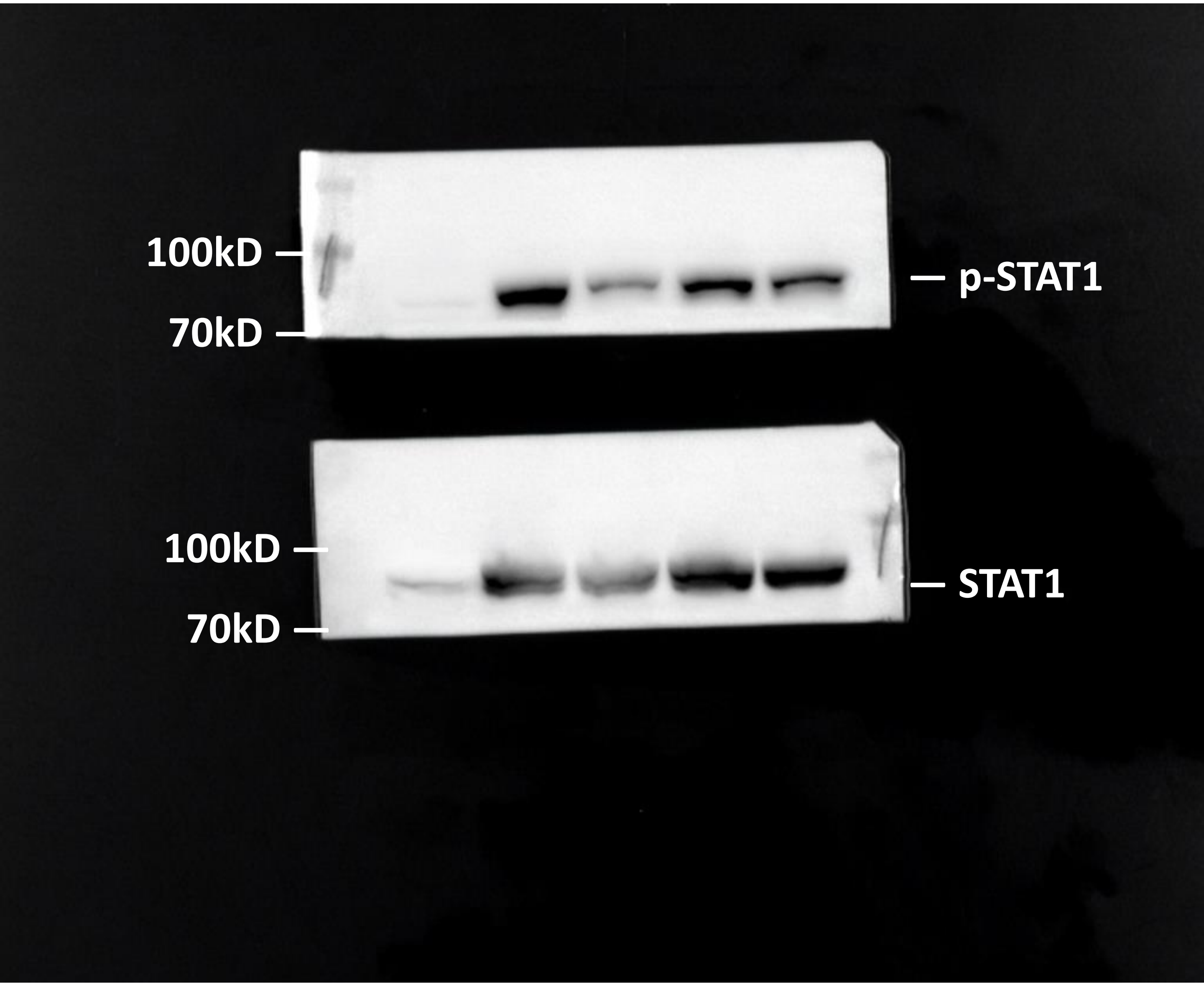

FIG2A

β-Actin(42kD)

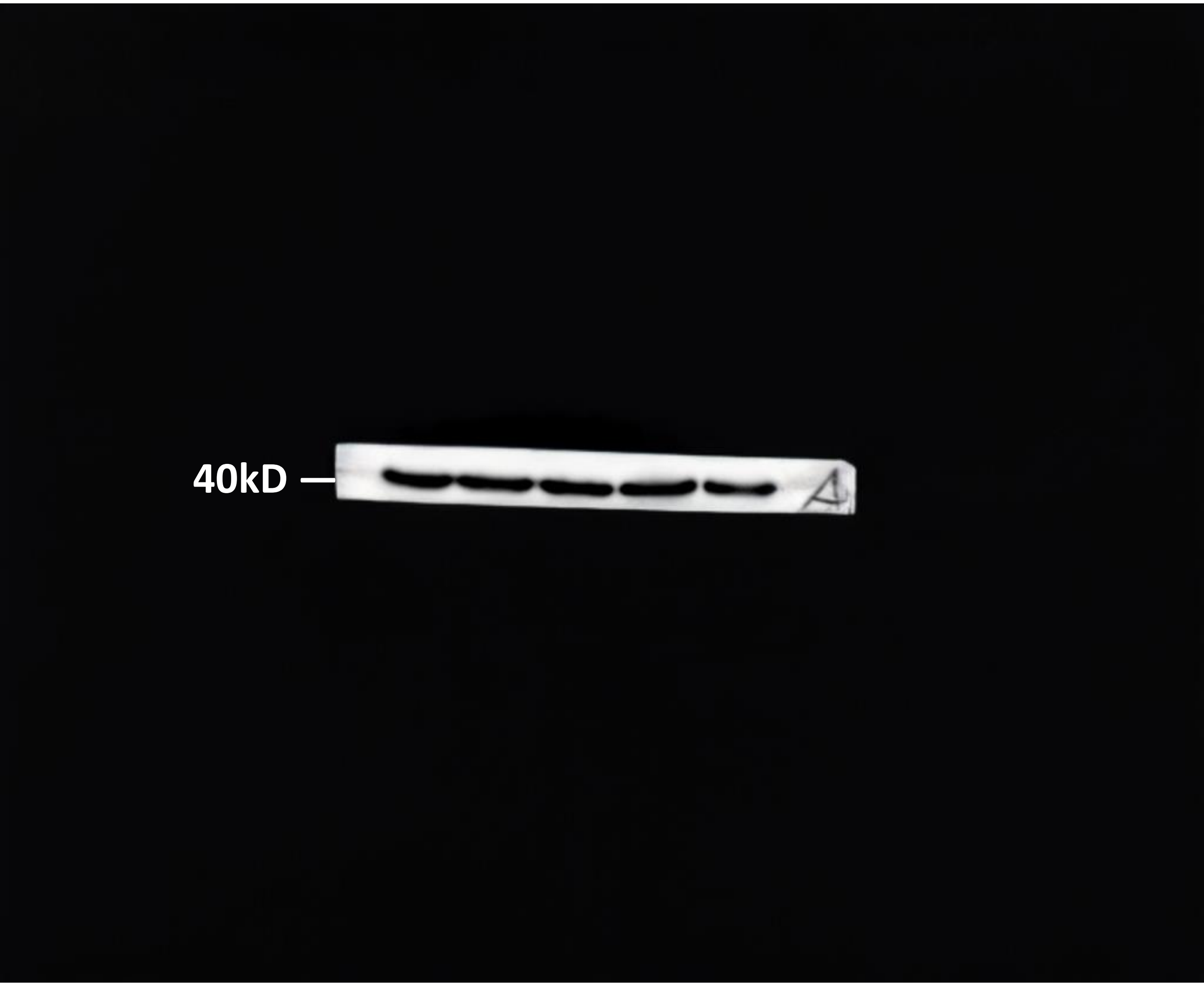

FIG2C
P2RX4(50kD)

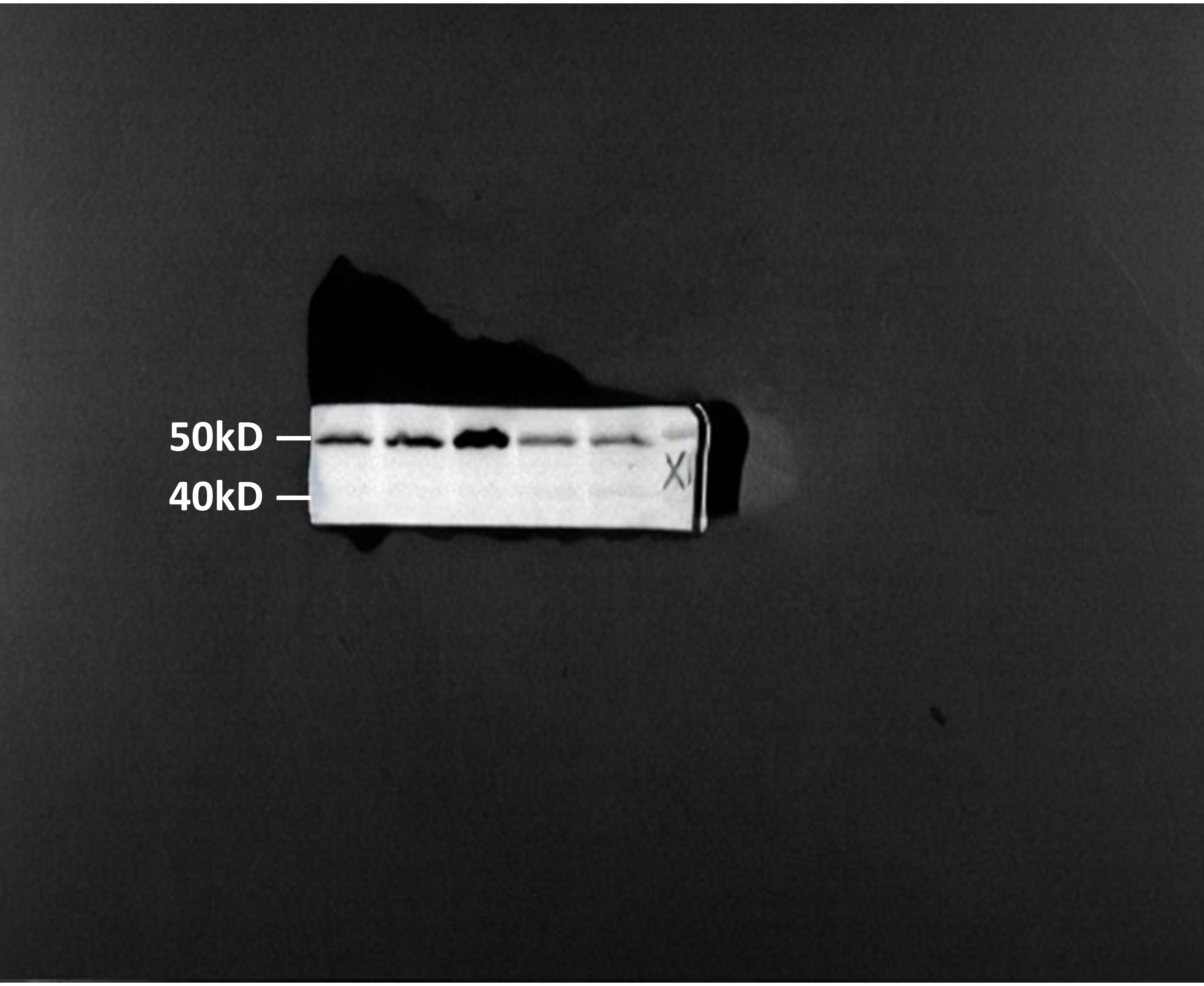

FIG2C

β-Actin(42kD)

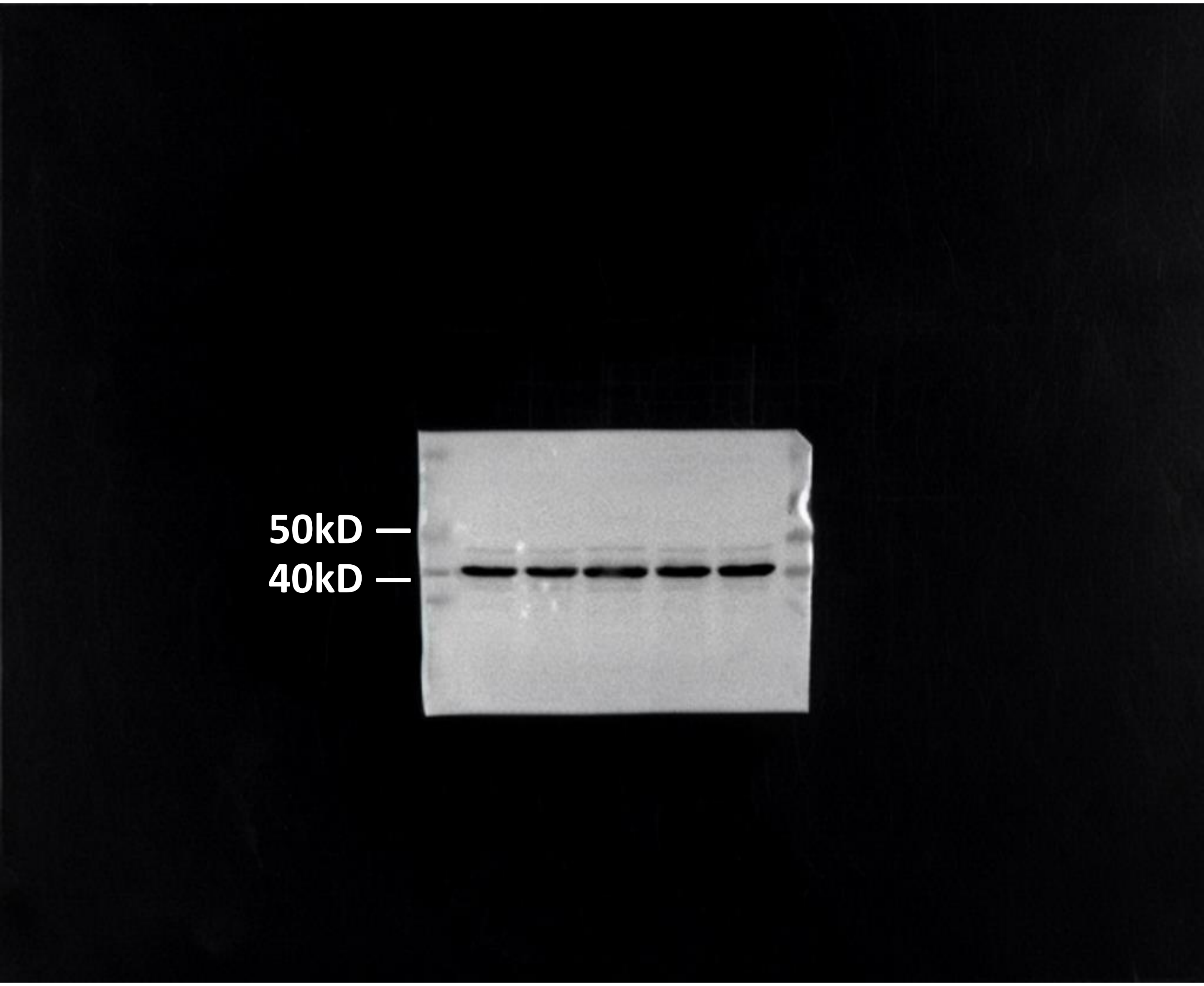

FIG2C

p-STING(41kD); STING(37kD)

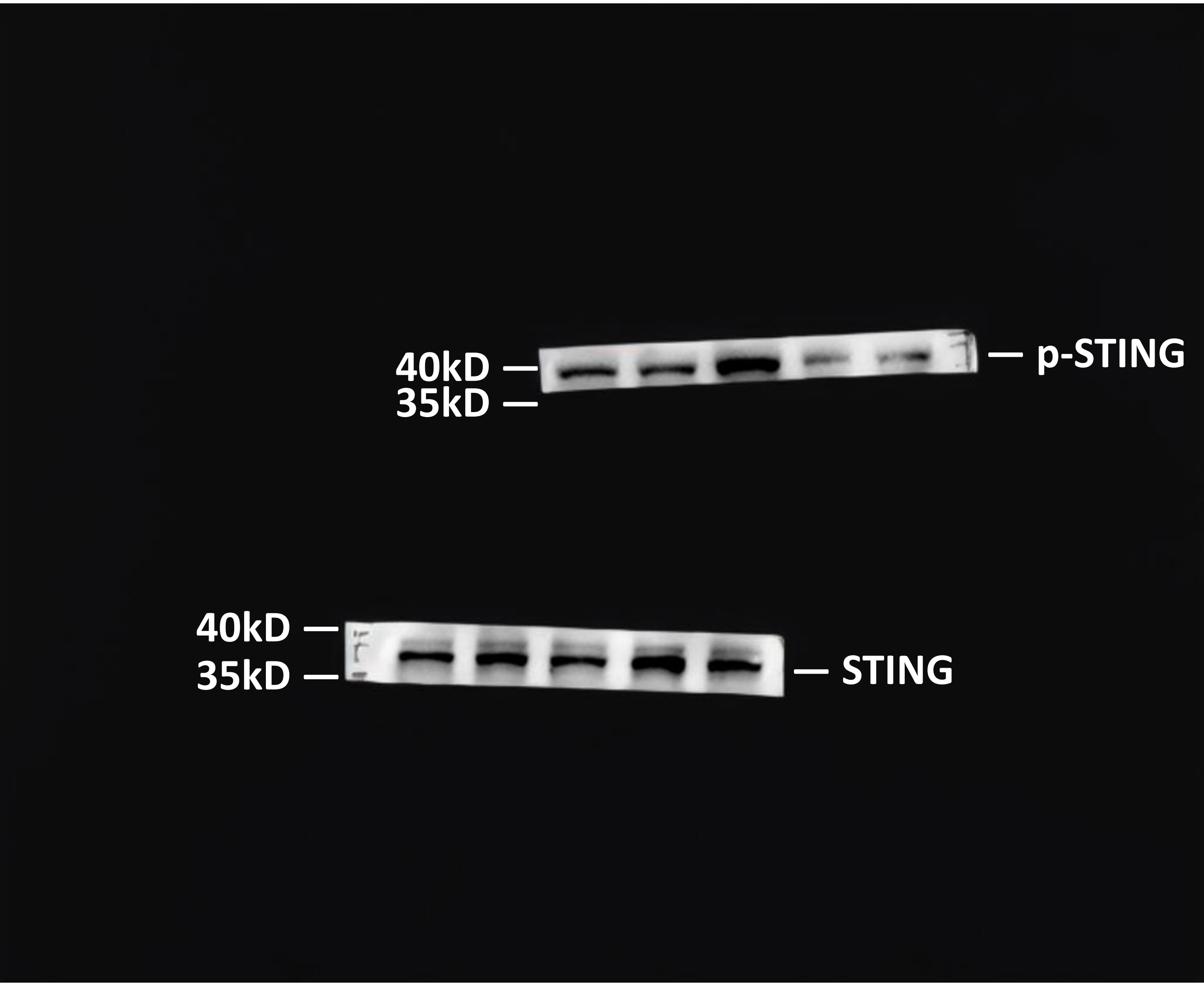

FIG2C

p-IRF3(55kD); IRF3(55kD)

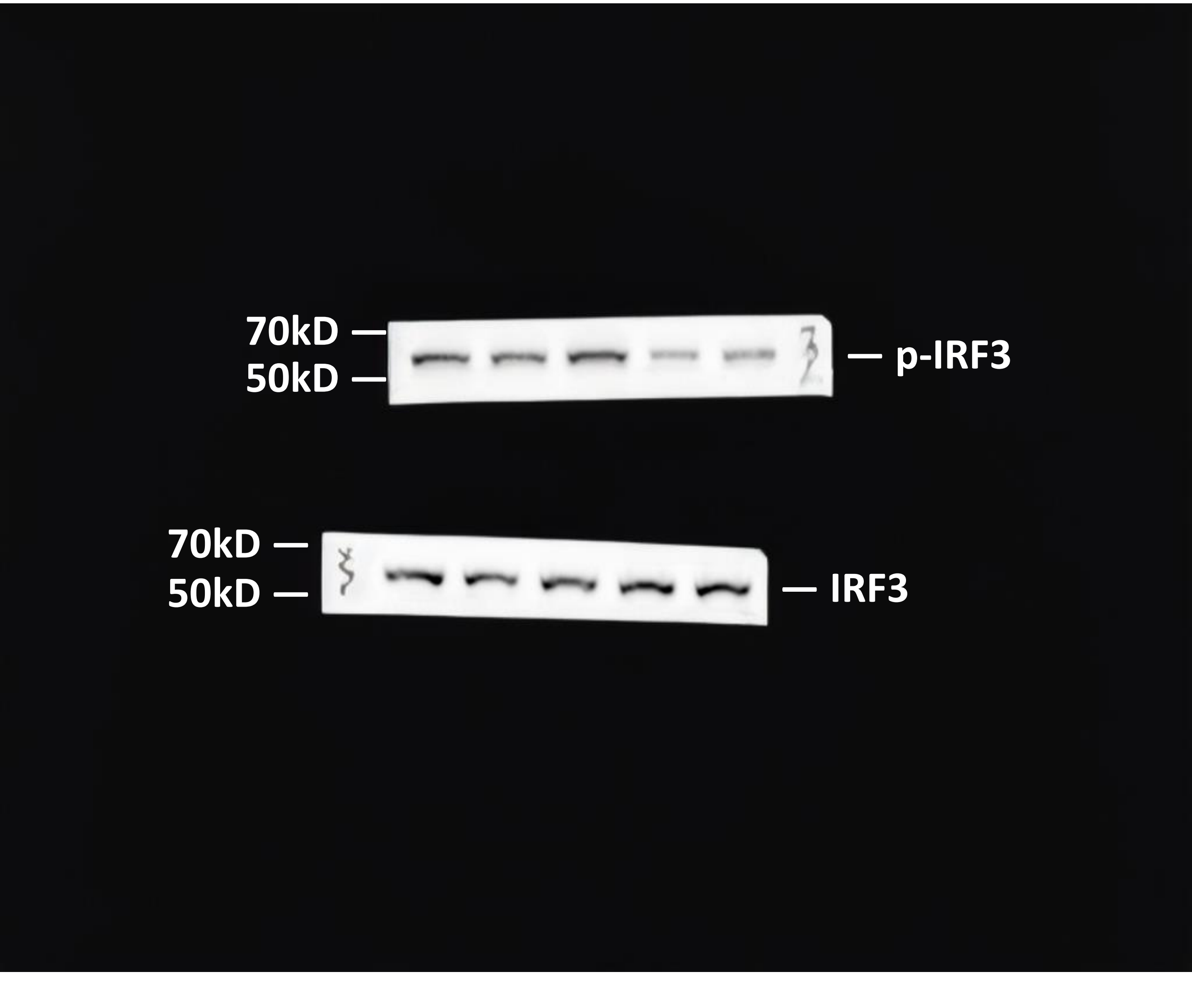

FIG2C

p-STAT1(91kD); STAT1(91kD)

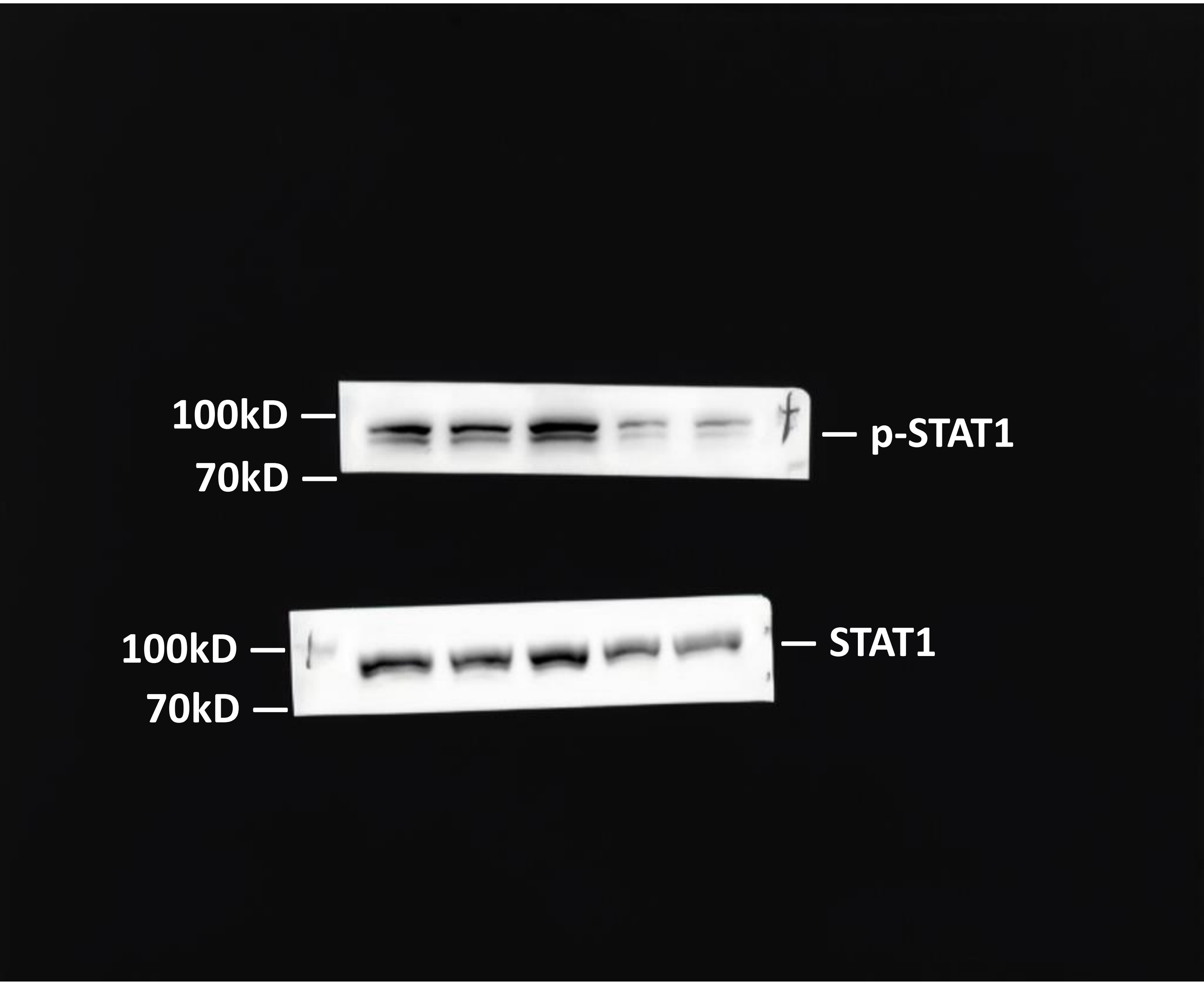

FIG2C

β-Actin(42kD)

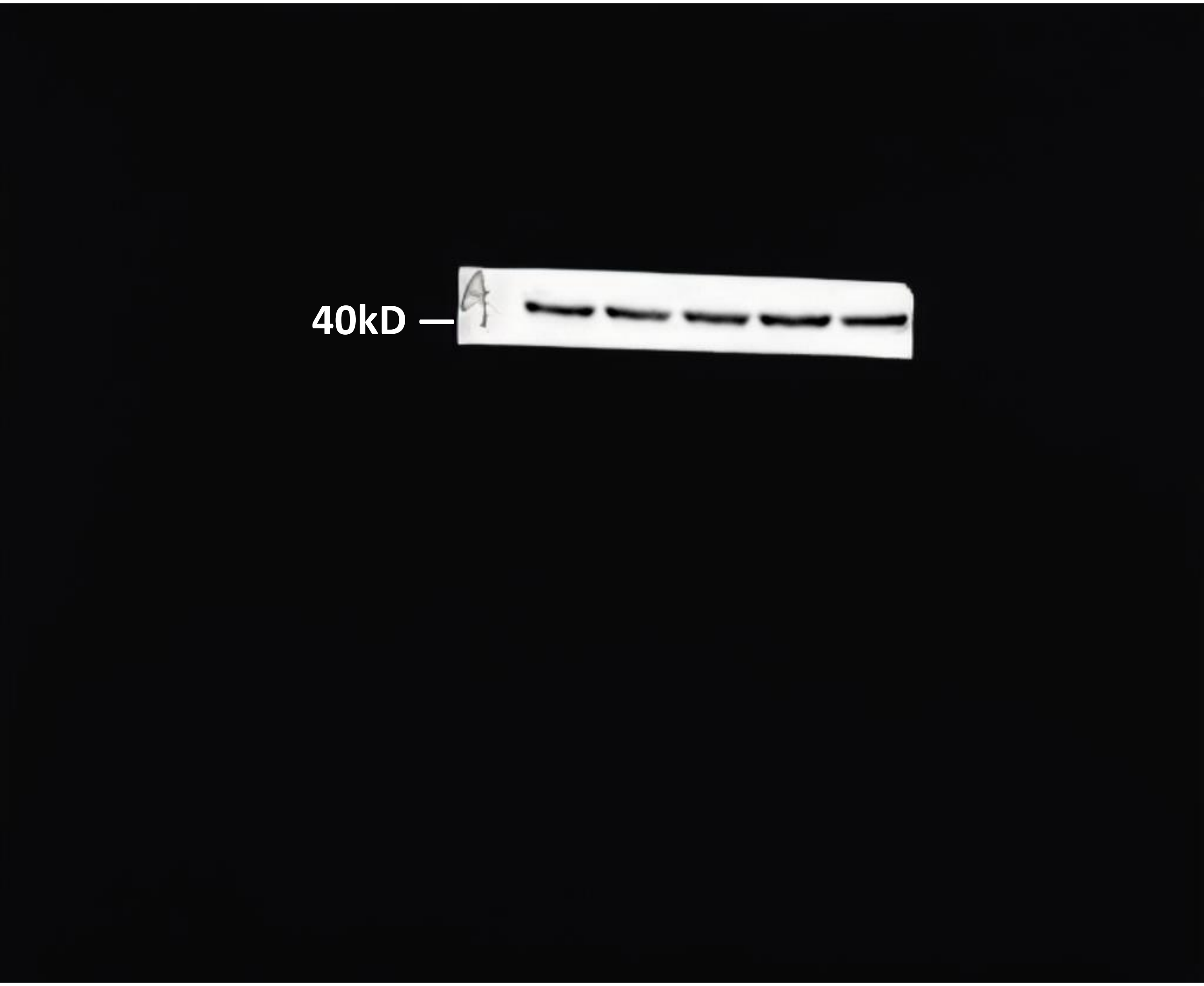

FIG4A
P2RX4(50kD)

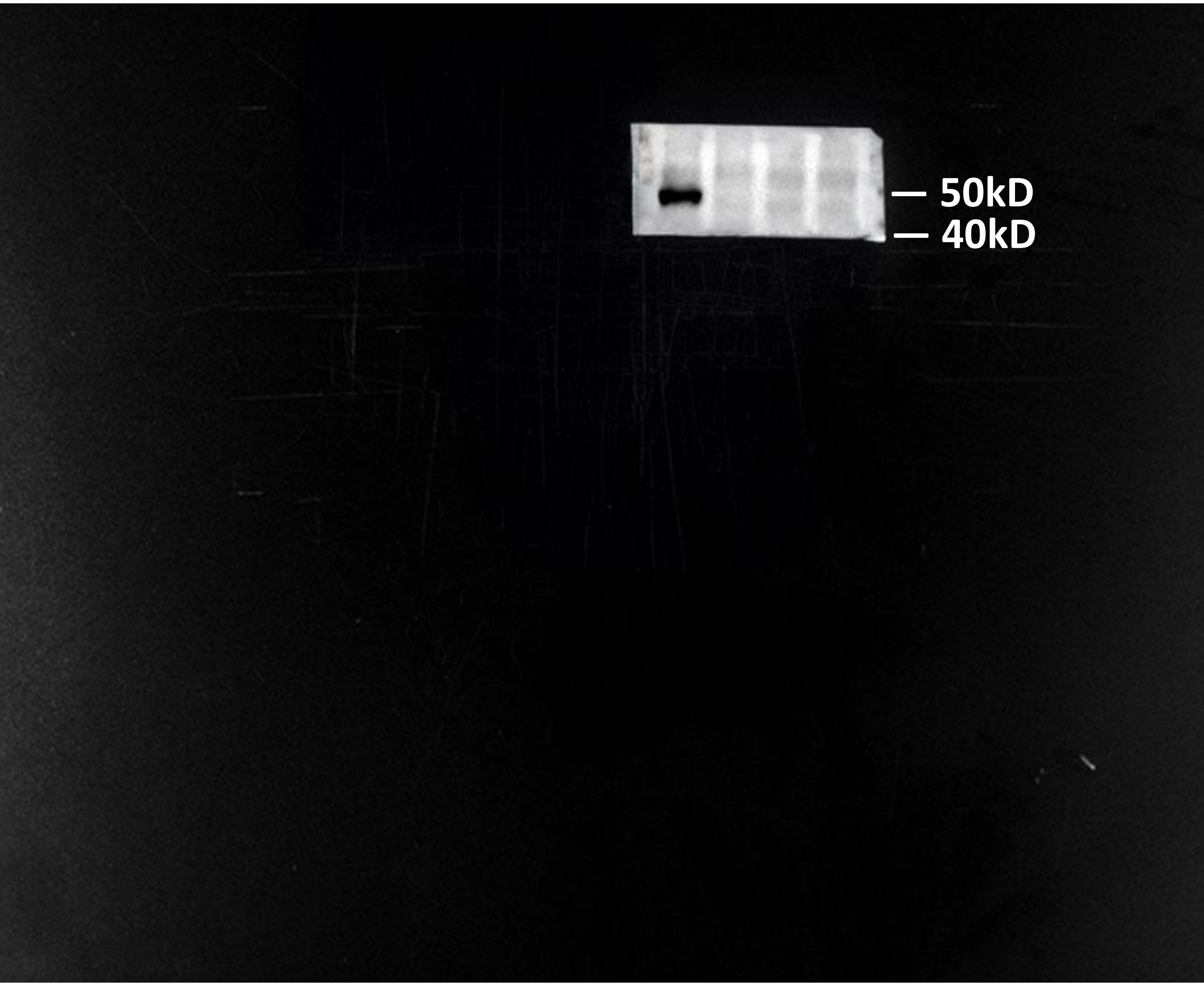

FIG4A

β-Actin(42kD)

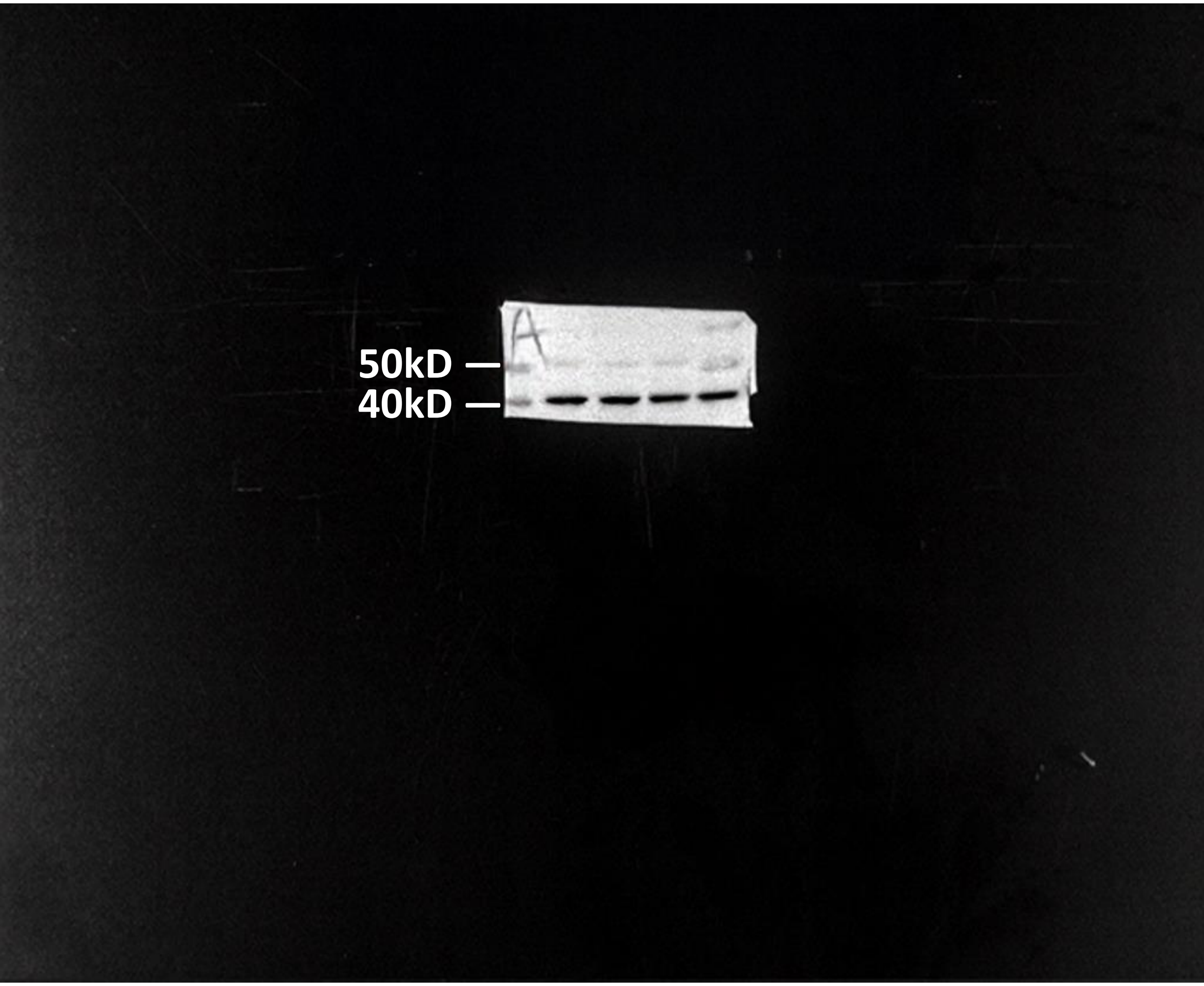

FIG6A

P2RX4(50kD)

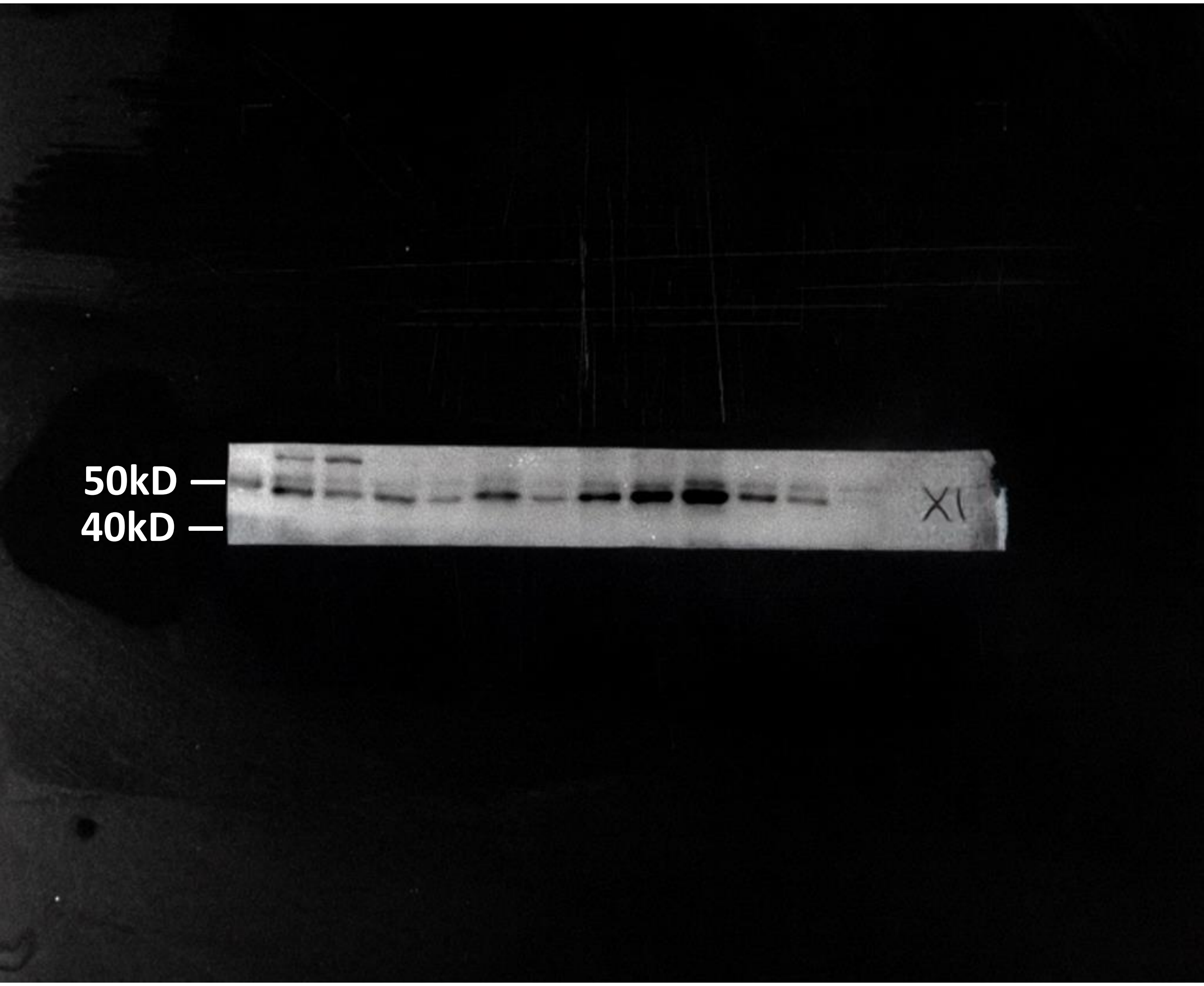

No. 1-6

FIG6A

β-Actin(42kD)

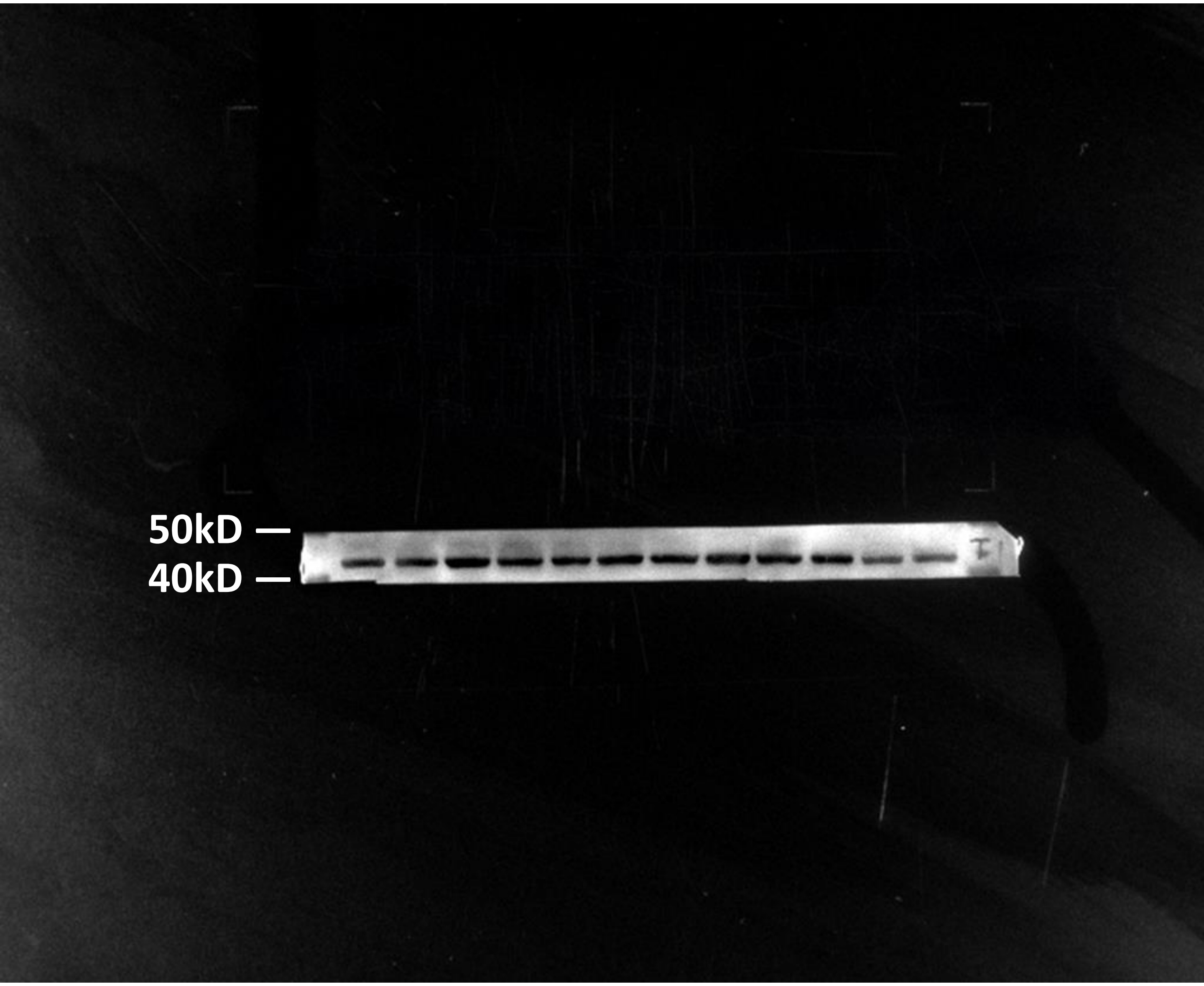

No. 1-6

FIG6A

P2RX4(50kD)

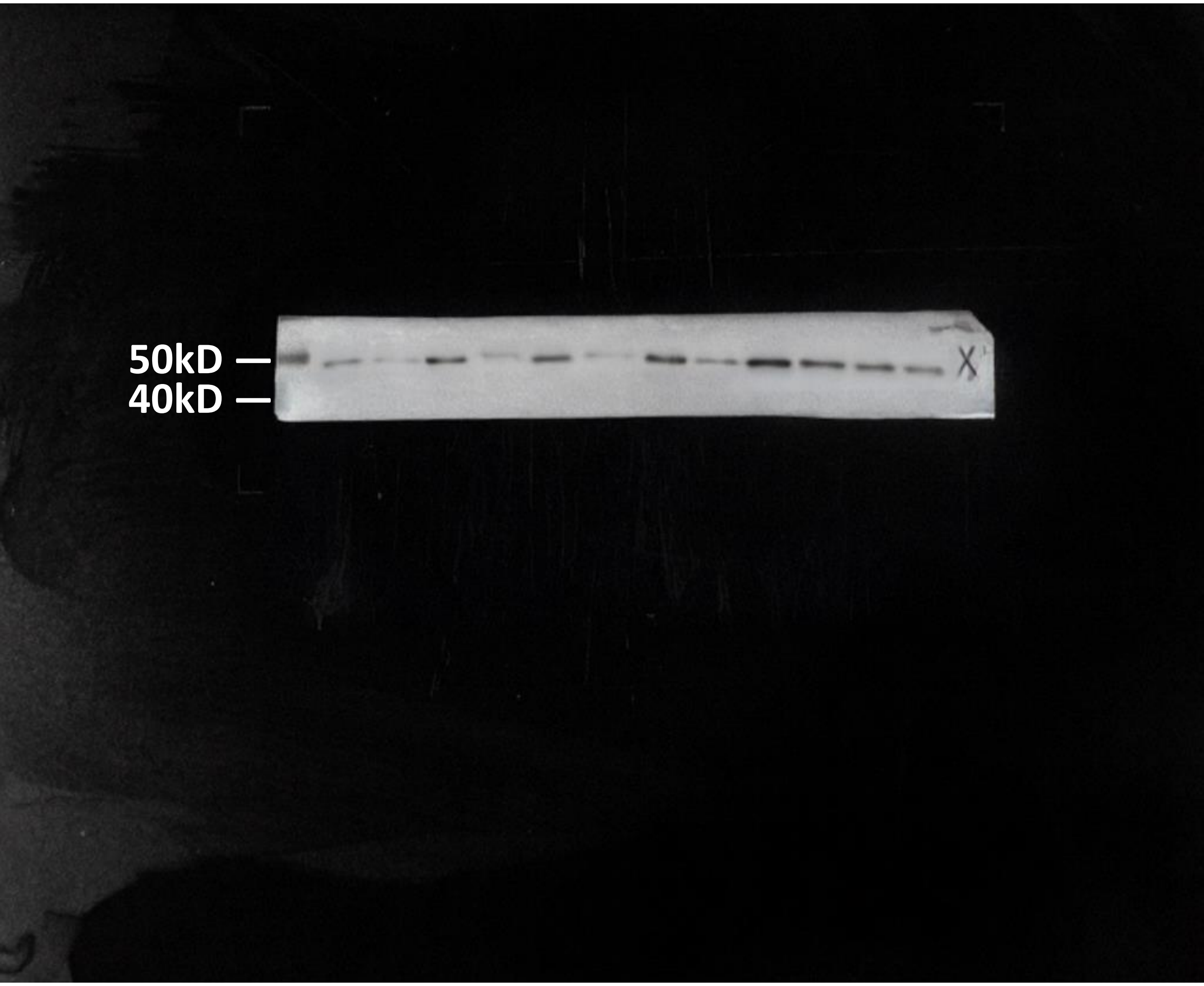

No. 7-12

FIG6A

β-Actin(42kD)

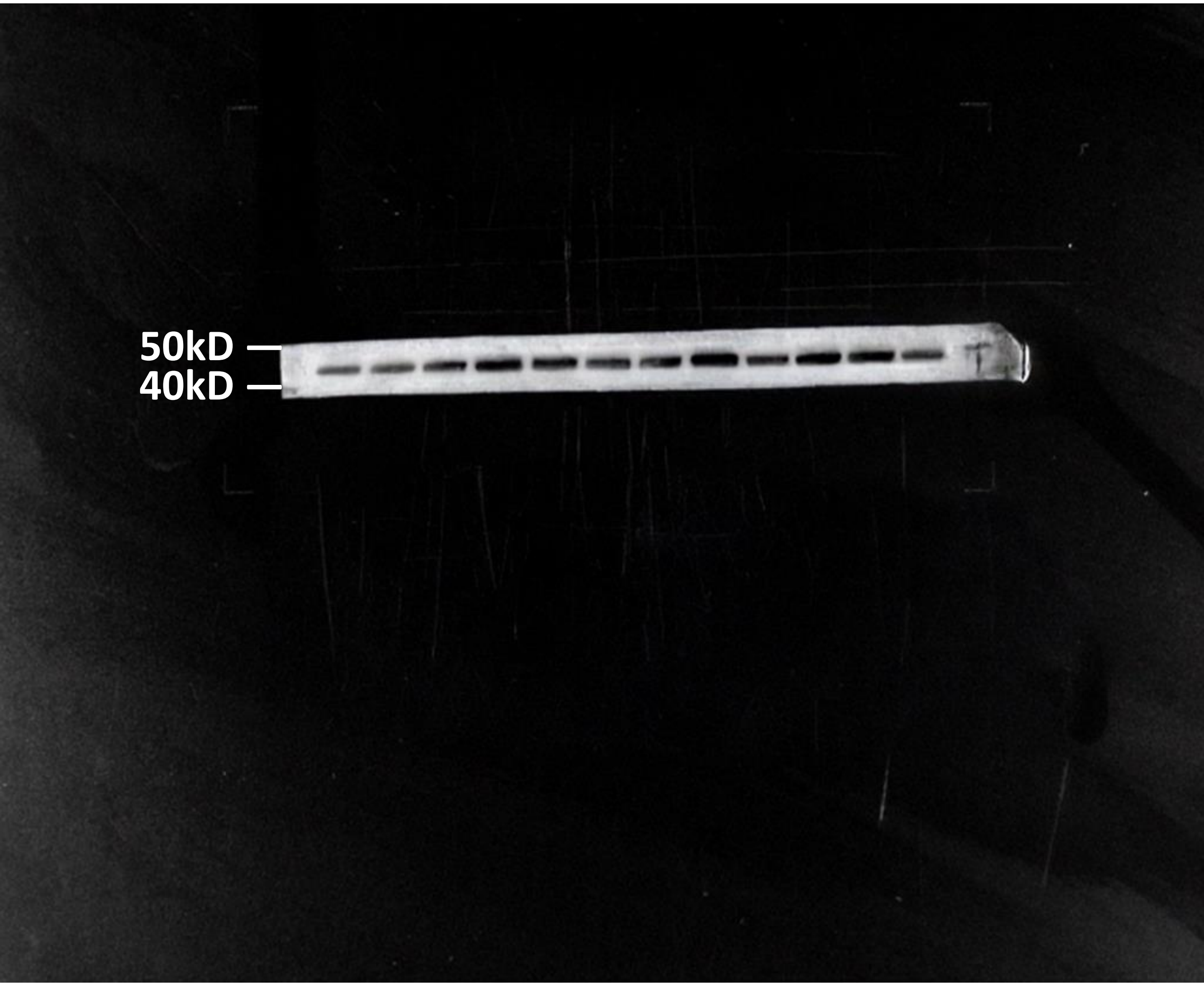

No. 7-12

FIG6A
P2RX4(50kD)

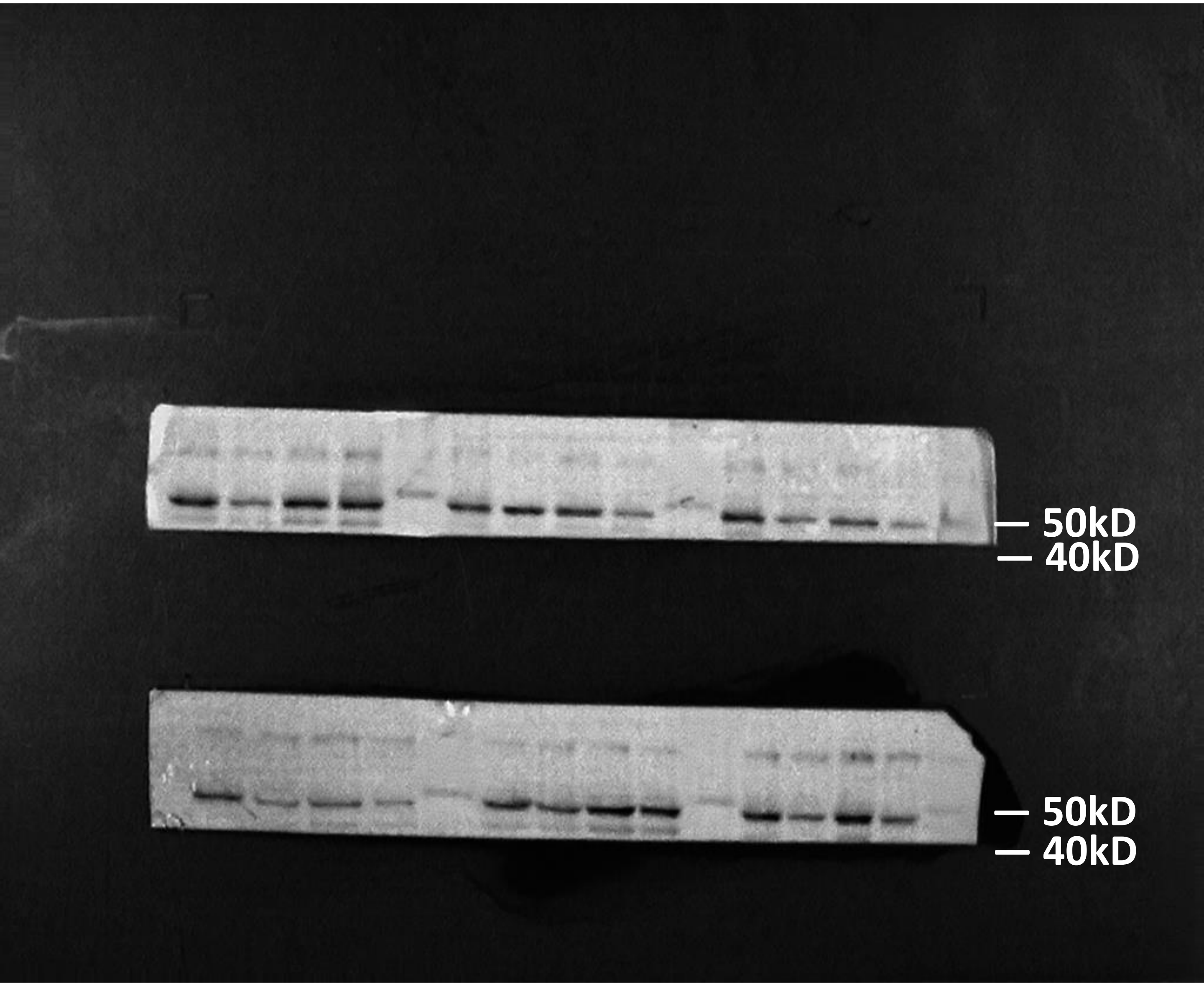

No. 13-24

FIG6A

β-Actin(42kD)

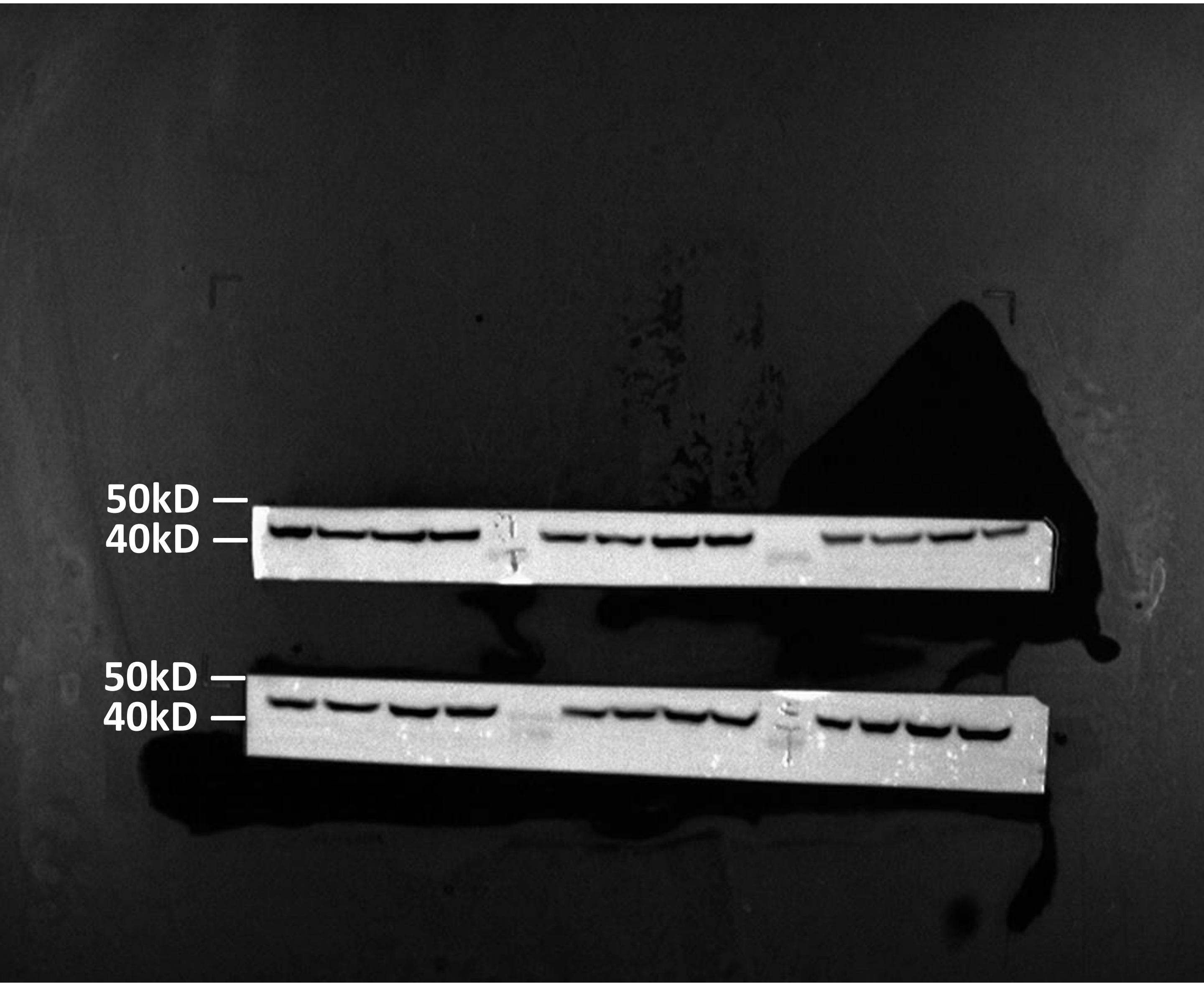

No. 13-24
